# Motivation on Intramolecular Through-Space Charge Transfer for the Realization of Thermally Activated Delayed Fluorescence (TADF)–Thermally Stimulated Delayed Phosphorescence (TSDP) in C^C^N Gold(III) Complexes and Their Applications in Organic Light-Emitting Devices

**DOI:** 10.1021/jacs.5c00121

**Published:** 2025-03-30

**Authors:** Panpan Li, Ziyong Chen, Ming-Yi Leung, Shiu-Lun Lai, Shun-Cheung Cheng, Wing-Kei Kwok, Chi-Chiu Ko, Mei-Yee Chan, Vivian Wing-Wah Yam

**Affiliations:** † Institute of Molecular Functional Materials and Department of Chemistry, The University of Hong Kong, Pokfulam Road, Hong Kong, P. R. China; ‡ Hong Kong Quantum AI Lab Limited, 17 Science Park West Avenue, Pak Shek Kok, Hong Kong, P. R. China; § Department of Chemistry, City University of Hong Kong, Tat Chee Avenue, Kowloon Tong, Hong Kong, P. R. China

## Abstract

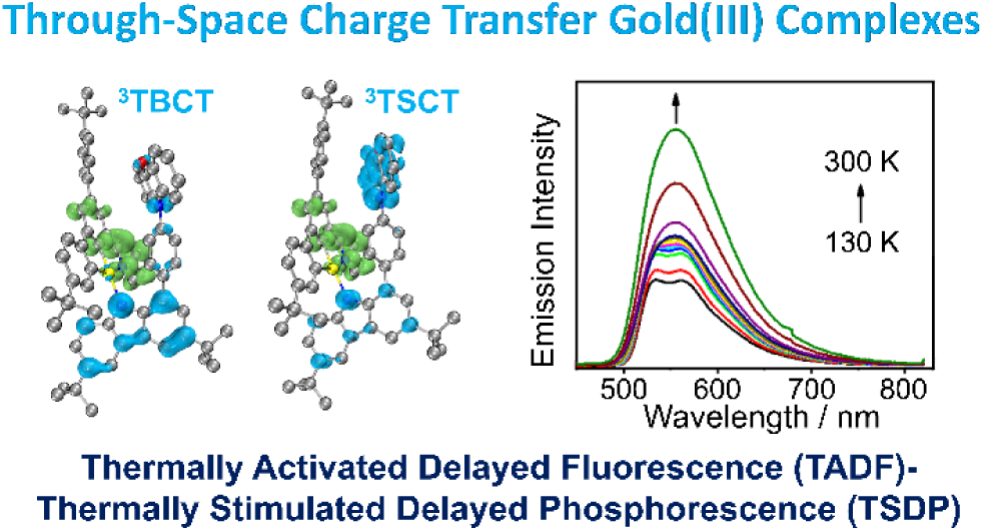

Thermally activated delayed fluorescence (TADF) and the very recently established thermally stimulated delayed phosphorescence (TSDP) are two promising approaches for enhancing the performance of organic light-emitting devices (OLEDs). Here, we have developed a new class of through-space charge transfer (TSCT) carbazolylgold(III) C^C^N complexes with unique TADF–TSDP properties by introducing a rigid arylamine on the carbazolyl auxiliary ligand. The highly twisted conformation between the C^C^N and carbazolyl ligands induces strong through-bond ligand-to-ligand charge transfer (TB-LLCT) character in their lowest singlet and triplet excited states, with small singlet–triplet energy gaps for efficient TADF. Moreover, the close spatial proximity between the cyclometalating ligand and the lateral arylamine enables appreciable intramolecular through-space electronic coupling that allows the generation of relatively low-lying triplet through-space ligand-to-ligand charge transfer (^3^TS-LLCT) excited states. The TADF–TSDP properties are verified by temperature-dependent emission, lifetimes, and ultrafast transient absorption studies. Interestingly, through better alignment with extended planarity and the strengthening of the electron-donating ability of the lateral arylamine, the enhanced through-space electronic coupling can effectively perturb the energies of ^3^TBCT, ^3^TSCT, and intraligand (^3^IL) excited states and thus manipulates the TSDP efficiency. Orange-emitting vacuum-deposited OLEDs made with these gold(III) complexes demonstrate respectable maximum external quantum efficiencies of >10% and long operational half-lifetimes of up to 65,314 h at a luminance of 100 cd m^–2^. This work not only demonstrates the realization of interesting TADF–TSDP and TSCT properties in the gold(III) C^C^N cyclometalated system but also enriches the diversity of molecular design for high-performance TSDP and TSCT emitters.

## Introduction

Organic light-emitting device (OLED) has become one of the leading technologies for full-color displays and lighting systems, significantly benefiting from innovations in both materials design and device engineering.^1^ Of particular significance is the development of transition-metal-based phosphorescent materials, whereby the heavy atom effect-induced strong spin–orbit coupling (SOC) promotes singlet excited states to undergo intersystem crossing (ISC) and allows radiative decay from the triplet excited states/excitons.^2^ Such strategy can fully harvest all excitons for the realization of 100% internal quantum efficiency for OLEDs.^2^ In the past decade, rational molecular design of organic emitters has been carried out to realize thermally activated delayed fluorescence (TADF), involves the upconverting of dark triplet excitons into emissive singlet excitons via reverse intersystem crossing (RISC), providing an alternative approach to fully harvest all the singlet and triplet excitons.^3^

To achieve efficient TADF emitters, the rate of RISC must be large. Reducing the spatial overlap between their highest occupied molecular orbital (HOMO) and lowest unoccupied molecular orbital (LUMO) is essential to minimize energy difference between the lowest-energy singlet (S_1_) and triplet (T_1_) excited states (Δ*E*_S1–T1_), which is one of the prerequisites for efficient RISC.^4^ To this end, a large amount of research have been focused on the design of molecules capable of through-bond charge transfer (TBCT) by constructing twisted donor–acceptor (D–A) molecular frameworks^5^ via introducing steric effect or bridging heteroatoms with opposite resonance effects into the polycyclic aromatic hydrocarbons.^6,7^ However, these approaches for reducing the Δ*E*_S1–T1_ would inevitably decrease the oscillator strength for fluorescence decay.^8^ On the other hand, efforts have been made to optimize performance by using a new strategy involving intramolecular through-space charge transfer (TSCT) architecture with D and A segments positioned in a close proximity in space and orientation. These TSCT molecules have intrinsically small Δ*E*_S1–T1_; more importantly, the spatial π–π interaction-promoted radiative process can be regulated by the distance and orientation between D and A motifs.^9−12^ Significant enhancement in OLED performance has been demonstrated in these TADF emitters with TBCT and TSCT emissive excited states.^13−18^

With a deeper understanding of the excited-state dynamics, TADF has also been unambiguously identified in transition metal complexes with short emission lifetimes down to sub-microseconds,^19^ providing great potentials for development of both efficient and stable high-energy emitters. More importantly, for the first time, the RISC process with activation energy determination in gold(III) complexes has been directly substantiated by temperature-dependent ultrafast transient absorption studies by our group.^20^ The work on gold(III) complexes has been further extended to demonstrate an innovative emission mechanism of thermally stimulated delayed phosphorescence (TSDP), where the T_1_ state can act as a mediator to capture nonradiative excitons from the emissive second lowest-energy triplet (T_1_′) excited state, and at the same time to recycle them by thermally activated upconversion to the T_1_′ state via reverse internal conversion (RIC).^21,22^ Particularly, the presence of the aryl substituent on the triazine-based C^N^C cyclometalated ligand has been shown to play an important role in triggering the TSDP mechanism.^21^ It has been demonstrated that the efficiency of TSDP and the emission energy can be fine-tuned through a deliberate structural design by varying the ligands and the polarity of the surrounding environment.

**Figure 1:**
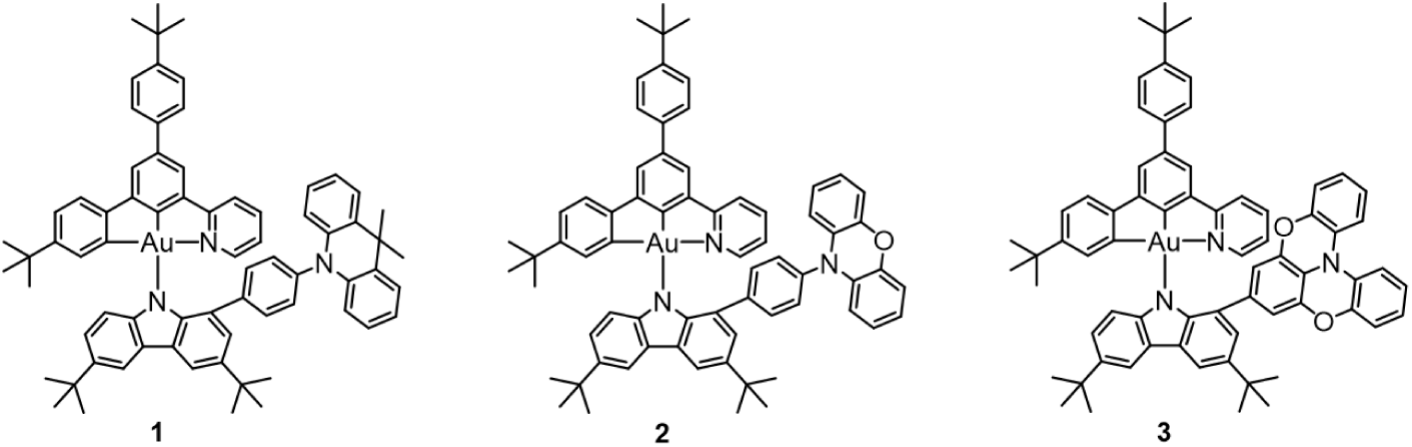
Chemical structures of complexes **1**–**3**.

Based on our expertise in designing various luminescent gold(III) complexes for OLEDs and the promising device performance of TSCT emitters, it would be interesting to introduce the intramolecular TSCT character into gold(III) complexes and investigate its role in mediating TADF and/or TSDP processes. Herein, we report the design and synthesis of a new class of TADF–TSDP C^C^N carbazolylgold(III) complexes by incorporating various strong and rigid lateral arylamine moieties, i.e., 9,9-dimethyl-9,10-dihydroacridine (DMAC), 10*H*-phenoxazine (PXO) and benzo[5,6][1,4]oxazino[2,3,4-*kl*]phenoxazine (DPXO), at the 1-position of the carbazolyl ligand to prepare complexes **1**–**3** respectively (Figure 1). Notably, it is found that both the S_1_ and T_1_ states of this class of gold(III) complexes display a substantial through-bond carbazolyl ligand-to-cyclometalating C^C^N ligand charge transfer (TBCT) character with small Δ*E*_S1–T1_ < 30 meV.^23,24^ The strong electron-donating strength of the lateral arylamine, together with the close proximity to the cyclometalating C^C^N ligand, has been found to induce considerable through-space electronic coupling and thus the TSCT excited states become energetically close-lying to the TBCT excited states. Meanwhile, it is worth noting that the enhanced planarity of the lateral arylamine can effectively strengthen the intramolecular through-space electronic interaction and induce mixing between ^3^TBCT and ^3^TSCT states for enhanced RIC efficiency, as supported by a smaller activation energy determined by temperature-dependent ultrafast transient absorption studies when comparing **2** and **3** to **1**. Introduction of a lateral arylamine moiety would introduce TSCT and would rigidify the molecule due to the locking up of the molecular motion as well as the preferential stacking mode to maximize the D–A CT interaction, leading to excellent thermal stability of the complexes. Orange-emitting vacuum-deposited OLEDs fabricated with this class of complexes are shown to achieve maximum EQEs of 10.9% and small efficiency roll-offs especially at high dopant concentrations. More importantly, respectable high operational stabilities with half-lifetimes (LT_50_) of up to 65,314 h projected at an initial luminance of 100 cd m^–2^ have been realized.

## Results and Discussion

### Synthesis and Characterization

The chlorogold(III) precursor, [Au{4-*^t^*BuC^C(4-*^t^*BuC_6_H_4_)^N}Cl]^25−28^ and the carbazolyl auxiliary ligands L1–L3^19^ were prepared according to the reported literature procedures with modifications. Complexes **1**–**3** (Figure 1) were prepared by reacting the chlorogold(III) precursor with the corresponding deprotonated *N*-carbazolyl ligand in the presence of sodium hydride in anhydrous tetrahydrofuran (Scheme S1). All the complexes have been fully characterized by ^1^H and ^13^C{^1^H} NMR spectroscopy, high-resolution electrospray-ionization (HR-ESI) mass spectrometry and elemental analysis. The identities of complexes **1–3** have further been confirmed by single-crystal X-ray diffraction (XRD) analysis. Moreover, as revealed by the thermogravimetric analysis (TGA) (Figure S1 and Table S1), all the complexes exhibit high robustness with decomposition temperatures (*T*_d_) of 336–406 °C. Particularly, **2** (404 °C) and **3** (406 °C) have been revealed to show significantly higher *T*_d_ than the tridentate C^C^N analogue, [Au{4-*^t^*BuC^C(4-*^t^*BuC_6_H_4_)^N}(Cbz)] (347 °C),^25^ as well as the tetradentate analogue, [Au{4-*^t^*BuC^C(4-*^t^*BuC_6_H_4_)^N_(py}_^N_(Cbz)_}] (354 °C),^29^ illustrating a positive influence of strong through-space electronic interactions between the lateral arylamine and the cyclometalating C^C^N ligand on improving the thermal stability.^19,30^ The presence of through-space D–A charge transfer (CT) will rigidify the molecule due to the locking up of the molecular motion as well as the preferential stacking mode to maximize the D–A CT interaction. Also, the increased π-surface of the lateral group will deter the rotation of the lateral moiety and the structure will be stabilized by the increase in π-surface for the D–A/π–π interaction of the lateral pendant group with the pincer ligand, further contributing to the superior thermal stability as well as the enhanced photoluminescence performance of complexes **2** and **3**.

### X-ray Crystal Structures

**Figure 2:**
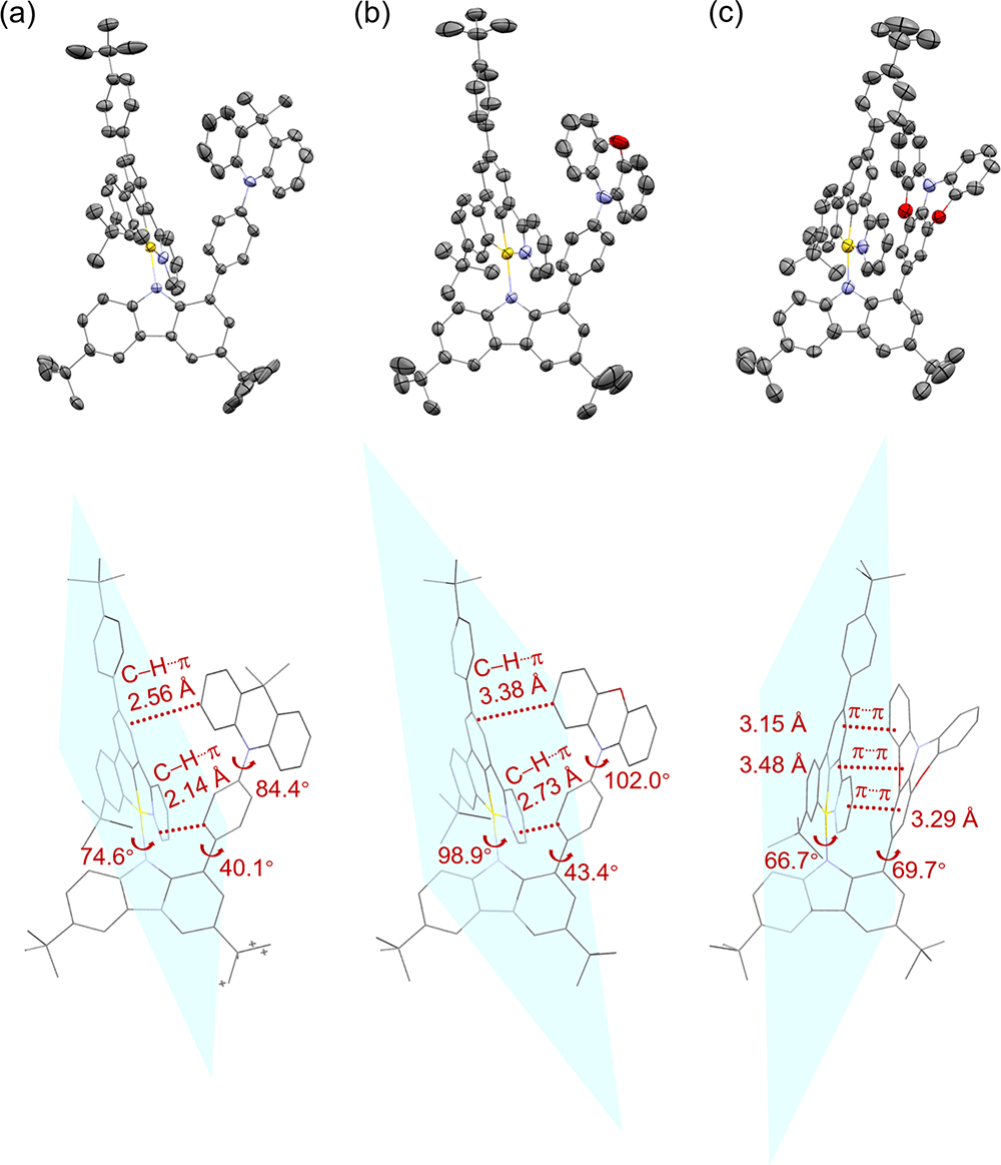
Perspective view of the crystal structures with short contacts of complexes (a) **1**, (b) **2** and (c) **3**. Thermal ellipsoids are drawn at the 50% probability level. The hydrogen atoms and the solvent molecules are omitted for clarity.

Single crystals of **1**–**3** have been obtained via slow solvent diffusion at room temperature to investigate the molecular alignments of different lateral arylamines with respect to the cyclometalating ligand (see Tables S2–S4). Bite angles of the C^C^N tridentate ligand are found to be in the range of 79.3–80.9° in **1**–**3**, suggesting a distorted square-planar geometry adopted by the gold(III) center in all complexes (Table S5). Specifically, for complex **1** with a lateral *N*-phenyl DMAC, the phenyl ring is tilted from its adjacent DMAC and carbazole by *ca*. 84.4° and 40.1°, respectively, due to the presence of steric H···H repulsion (Figure 2a). Meanwhile, a torsion angle of *ca*. 74.6° between planes of the cyclometalating ligand and the carbazolyl moiety has been determined. All of these angles give rise to a face-to-face alignment between the DMAC unit and the cyclometalating ligand with short C–H···π distances of 2.14–2.56 Å. Upon the replacement of DMAC unit in **1** by PXO unit, an almost perpendicular arrangement between the PXO unit and its adjacent phenyl ring (102.0°) as well as the cyclometalating and the carbazolyl ligands (98.9°) are found, suggesting the preference for a preorganized cofacial alignment between the PXO unit and the cyclometalating ligand in **2** (Figure 2b). In the context of **3**, both the lateral DPXO and the cyclometalating ligand are highly twisted from the carbazolyl ligand with torsion angles of *ca*. 69.7° and 66.7°, respectively, and close π···π distances of *ca*. 3.15–3.48 Å between DPXO and the cyclometalating ligand are revealed, resulting in a more compact cofacial arrangement (Figure 2c). Such a large spatial overlap and short π···π distance between DPXO and the cyclometalating ligand would facilitate a strong mutual electronic coupling and thus the formation of low-lying TSCT excited states.^31^ Additionally, not only is the presence of multiple noncovalent interactions between the lateral arylamine and the cyclometalating ligand for all the complexes believed to rigidify the complex molecule, but also the lateral arylamine provides steric protection about the metal center, suppressing intermolecular interactions and thus excimeric emission from this class of carbazolylgold(III) systems.

### Photophysical Properties

**Figure 3:**
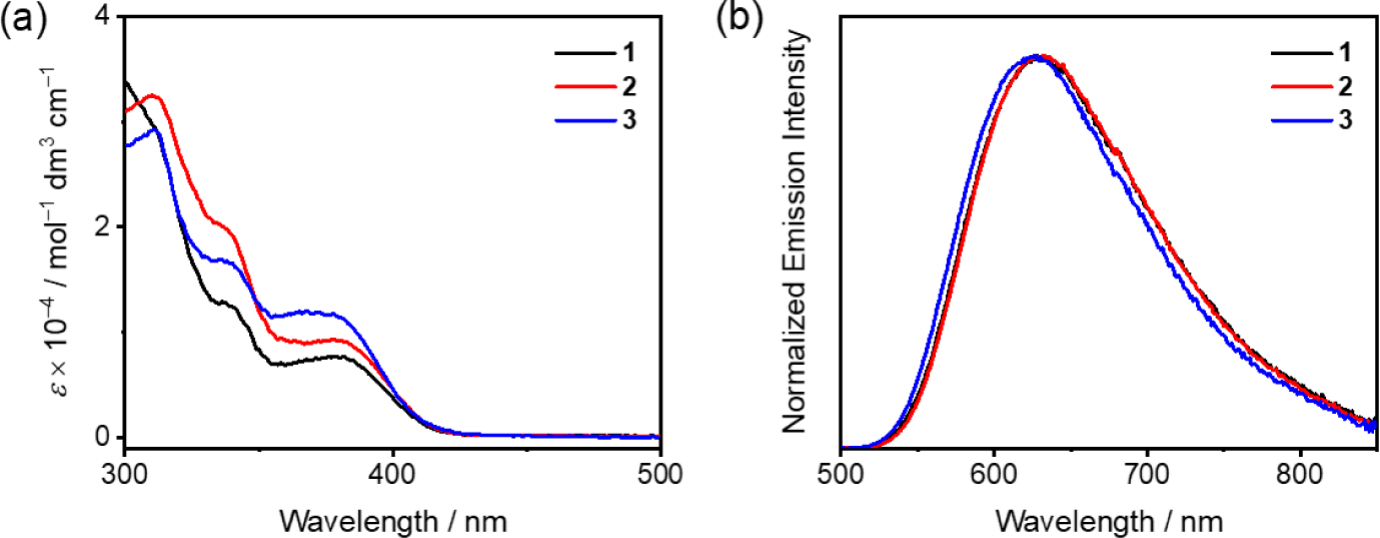
(a) UV–Visible absorption spectra and (b) normalized emission spectra of **1**–**3** in toluene at 298 K.

Steady-state photophysical properties of these gold(III) complexes in toluene solutions (10^–5^ M) and in doped 1,3-bis(carbazol-9-yl)benzene (mCP) thin films at 298 K have been investigated (Figures 3 and S2–S5, Table 1). In toluene solution, **1**–**3** generally show similar absorption profiles with high-energy vibronic absorption bands at *ca*. 300–370 nm and a low-energy structureless absorption band at *ca*. 370–410 nm, regardless of the identity of the lateral arylamine on the carbazolyl ligand (Figure 3a). With reference to the electronic absorption spectra of L1–L3 (Figure S2) and structurally related gold(III) complexes,^25−27^ the high-energy absorption bands are assigned as IL [π→π*(C^C^N)], [π→π*(carbazolyl ligand)] and [π→π*(lateral arylamine)] transitions, while the low-energy absorption band is mainly attributed to the metal-perturbed IL [π→π*(C^C^N)] transitions mixed with some IL charge transfer (ILCT) [π(aryl)→π*(pyridine)] as well as through-bond ligand-to-ligand charge transfer (TB-LLCT) [π(carbazolyl ligand)→π*(C^C^N) and through-space ligand-to-ligand charge transfer (TS-LLCT) [π(lateral arylamine)→π*(C^C^N)] characters. Such assignments are well supported by computational studies (*vide infra*).

**Table 1:** Photophysical Data of Complexes **1**–**3**

Complex	Medium (*T*/K)	Absorption λ_max_/nm (*λ*_max_/dm^3^ mol^–1^ cm^–1^)	Emission λ_max_/nm (τ_0_/μs)	Φ_soln_^a^	Φ_film_^b^	*k*_r_^c^/s^–1^
**1**	Toluene (298)	312 (29060), 337 (12770), 380 (7690)	633 (0.48)	0.037		7.71 × 10^4^
	Glass (77)^d^		547 (4.4, 9.2)			
	Thin film (298)					
	5 wt % in mCP		558 (2.6, 5.5)		0.62	2.37 × 10^5^
	10 wt % in mCP		563 (2.7, 5.6)		0.51	1.91 × 10^5^
	15 wt % in mCP		569 (2.0, 4.0)		0.47	2.31 × 10^5^
	20 wt % in mCP		572 (2.0, 3.9)		0.49	2.48 × 10^5^
	Solid (298)		573 (4.2)			
	Solid (77)		550, 588 (6.6, 12.9)			
**2**	Toluene (298)	310 (32480), 335 (20340), 378 (9350)	633 (0.53)	0.038		7.17 × 10^4^
	Glass (77)^d^		549 (4.8, 9.3)			
	Thin film (298)					
	5 wt % in mCP		566 (2.3, 5.4)		0.40	1.72 × 10^5^
	10 wt % in mCP		567 (2.2, 4.7)		0.43	1.94 × 10^5^
	15 wt % in mCP		579 (1.9, 4.1)		0.33	1.73 × 10^5^
	20 wt % in mCP		581 (1.7, 3.6)		0.34	1.96 × 10^5^
	Solid (298)		555 (3.2, 5.4)			
	Solid (77)		532, 564 (6.2, 13.6)			
**3**	Toluene (298)	311 (29230), 335 (16820), 368 (11900)	626 (0.63)	0.040		6.35 × 10^4^
	Glass (77)^d^		549 (16.4, 62.6)			
	Thin film (298)					
	5 wt % in mCP		562 (4.3, 10.7)		0.51	1.20 × 10^5^
	10 wt % in mCP		563 (3.6, 8.0)		0.45	1.26 × 10^5^
	15 wt % in mCP		564 (3.3, 7.1)		0.42	1.28 × 10^5^
	20 wt % in mCP		566 (3.0, 6.2)		0.43	1.43 × 10^5^
	Solid (298)		549 (0.3, 1.1)			
	Solid (77)		523, 557 (3.6, 8.6)			

a The luminescence quantum yield, measured at room temperature using quinine sulfate in 0.5 M H_2_SO_4_ as the reference (excitation wavelength = 365 nm, Φ_lum_ = 0.546).

b Absolute luminescence quantum yield of thin films was measured with 320 nm excitation.

c Radiative decay rate *k*_r_ = Φ/τ.

d Measured in toluene matrix.

Upon excitation at λ ≥ 340 nm, **1**–**3** in toluene solution exhibit structureless emission bands, suggestive of a LLCT excited state origin (Figure 3b). Similar to the absorption energies, the emission energies of **1**–**3** are not very sensitive to the electronic nature of the lateral arylamine moiety on the carbazolyl ligand. It is worth noting that upon comparing the emission spectra in the solution state to those in the thin film solid state, blue shifts in the emission bands and considerably improved photoluminescence quantum yields (PLQYs) from 0.037–0.040 to 0.33–0.62 are found for all the complexes, which could be explained by luminescence rigidochromism^32^ arising from effectively suppressed intramolecular motions in the rigid matrix^33^ (Figures S3–S5 and Table 1). Notably, small emission spectral red shifts of 438 cm^–1^ for **1**, 456 cm^–1^ for **2** and 126 cm^–1^ for **3** are observed upon increasing the dopant concentration from 5 wt % to 20 wt %. These findings suggest that the introduction of a highly twisted donor moiety is a promising approach for suppressing intermolecular interactions and thus excimeric emissions.^34^ Unlike a single exponential emission lifetime value measured in the solution state, the photoluminescence (PL) decay characteristics of **1**–**3** in solid-state thin film at various dopant concentrations are found to be well described by a biexponential decay model, in which two distinct emission lifetimes in the microsecond regime can be clearly resolved (Table 1). It is found that the percentage contributions by the two microsecond lifetimes are dependent on the dopant concentration (Table S6), in which the percentage contribution of τ_1_ generally increases whereas that of τ_2_ generally decreases upon increasing the dopant concentration. Such observations may be reminiscent of the previously reported polarity-dependent TSDP gold(III) complexes, with an enhanced TSDP efficiency via RIC from the T_1_ (^3^LLCT) state to the T_1_′ (^3^IL) state by lowering the environmental polarity.^22^ Considering the larger dipole moments of **1**–**3** than mCP together with the stronger charge transfer character of T_1_ than that of T_1_′ (^3^IL) (see Computational Studies), the increased dopant concentration would enlarge the energy difference between T_1_ and T_1_′ (^3^IL), being unfavorable for the RIC process from the T_1_ state to the T_1_′ (^3^IL) state and thus the percentage contribution of τ_2_ decreases with increasing the dopant concentration. Such a TSDP emission hypothesis has been further supported by variable-temperature emission studies, time-resolved emission studies, transient absorption studies as well as computational studies.

### Variable-Temperature Emission Studies

**Figure 4:**
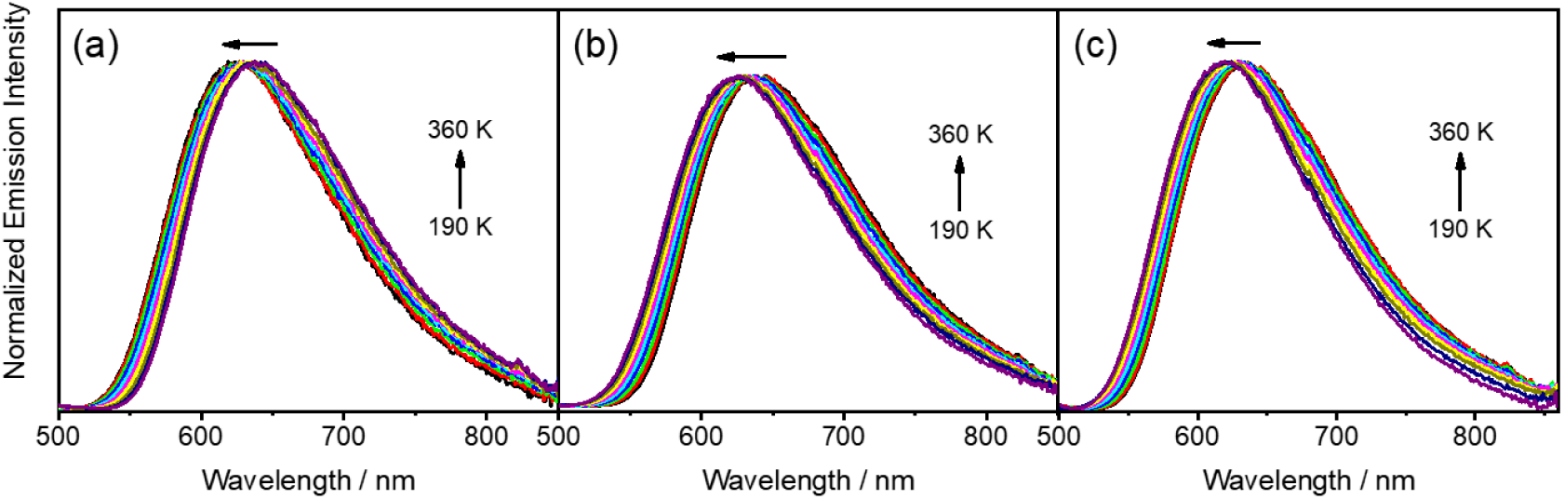
Normalized emission spectra of (a) **1**, (b) **2** and (c) **3** in degassed toluene solution showing a blue shift upon increasing temperature.

**Figure 5:**
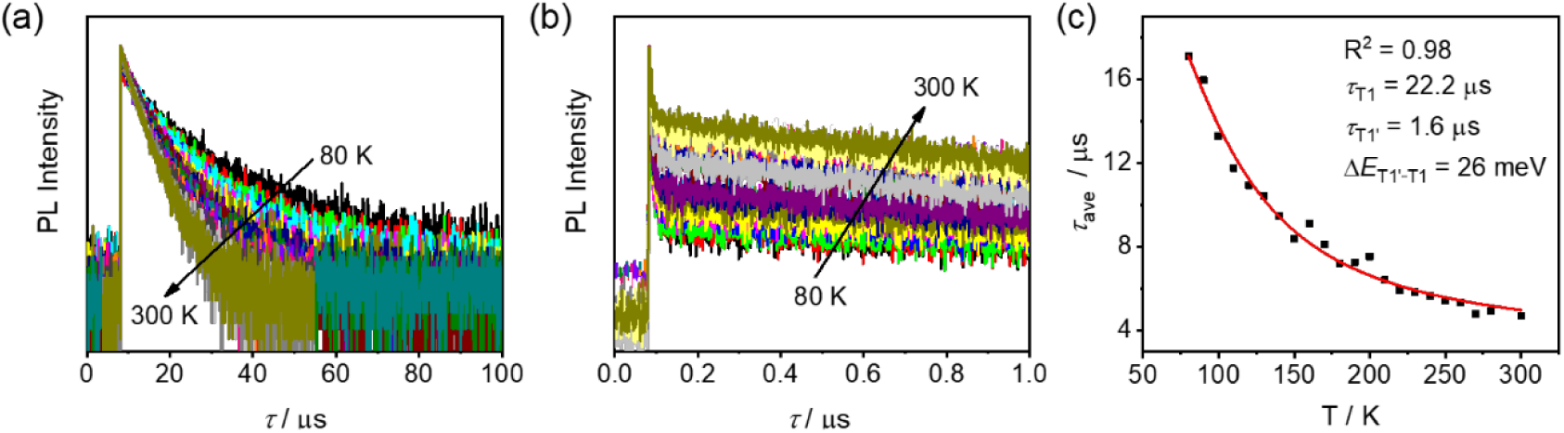
(a, b) PL time decay profiles of 20 wt % doped mCP thin film for **3** at 80–300 K and (c) the corresponding plot of average lifetime (τ_ave_) against temperature (excitation wavelength at 340 nm).

To further investigate the emission properties of this class of complexes, variable-temperature emission studies have been performed on **1**–**3** in toluene solution, the solid state and solid-state thin film (Figures 4, 5 and S6–S13). Upon heating from 190 to 360 K in degassed toluene solutions, the emission spectra of **1**–**3** show gradual blue shifts of 501 cm^–1^ (60 meV), 472 cm^–1^ (60 meV) and 332 cm^–1^ (40 meV), respectively (Figure 4). The emission intensities of **1** and **2** are found to decrease with increasing temperature, whereas an emission enhancement is observed for **3** upon increasing the temperature from 220 to 300 K. The blue shift indicates the possible occurrence of a thermally activated process.^21,22,26,27,35,36^ The reduced emission intensities for **1** and **2** could mainly be ascribed to the enhanced nonradiative deactivation at higher temperature (Figures S6 and S7). In contrast, the emission intensity growth for complex **3** can be rationalized by the strong π–π interactions between the cyclometalating C^C^N ligand and the lateral DPXO moiety, which restricts the intramolecular motions and hence greatly suppresses the nonradiative decay even with an increase in temperature (Figure S8).^34^ On the contrary, emission enhancements are observed for all three complexes in the more rigid solid state and the solid-state thin film upon increasing temperatures (Figures S9 and S10). In the solid state, blue shifts of 730 cm^–1^ (90 meV), 779 cm^–1^ (100 meV) and 906 cm^–1^ (110 meV) are observed for **1**–**3**, respectively, upon heating from 77 to 298 K. Such small blue shifts in emission maxima upon increasing temperature in both the solution and solid state are likely attributed to a thermally activated process between two close-lying excited states.

Variable-temperature time-resolved PL decay traces of **1**–**3** in rigid matrix from 80 to 300 K have also been acquired to investigate the emission origin. As illustrated in Figures 5a, S11a and S12a, the microsecond-scale decay components of 20 wt % **1**–**3** doped mCP thin films in the time range of 100 μs generally decrease with increasing temperature, which is typical of phosphorescence.^37,38^ By fitting the PL decay curves, two distinctive microsecond-scale lifetimes at various temperatures for doped thin films of **1**–**3** can be well resolved. The plot of averaged lifetime (τ_ave_)^39^ as a function of temperature can be well-fitted using a modified three-state Boltzmann equation for TSDP emitters (see Supporting Information).^21,40,41^ The lifetimes of the two triplet states estimated from the fitting (τ_T1_ and τ_T1′_) are in microsecond range, with small Δ*E*_T1′–T1_ of around 26 meV for all complexes (Figures 5c, S11c and S12c), suggesting that the thermally activated emission is possibly TSDP.^21,22^ Upon closer inspection of the PL decay curves in a shorter time range of 1 μs (Figures 5b, S11b and S12b), a nanosecond-scale time constant and a microsecond-scale time constant can be found (Tables S7a, S8 and S9). The nanosecond-scale decay component can be attributed to the prompt fluorescence from the S_1_ state. In addition, it is found that the contribution of the microsecond-scale decay component in this time range gradually increases with increasing temperature, possibly due to the activation of another thermal upconversion process with sufficient thermal energy, apart from TSDP observed in the longer time range of 100 μs.^21,22,42^ With reference to the fast ISC rates (10^10^–10^12^ s^–1^) in gold(III) complexes,^43^ the microsecond-scale decay component is tentatively assigned as TADF. With these results, the temperature-dependent emission mechanism for **1**–**3** is proposed to be a mixture of TADF and TSDP.

### Time-Resolved Emission Studies

**Figure 6:**
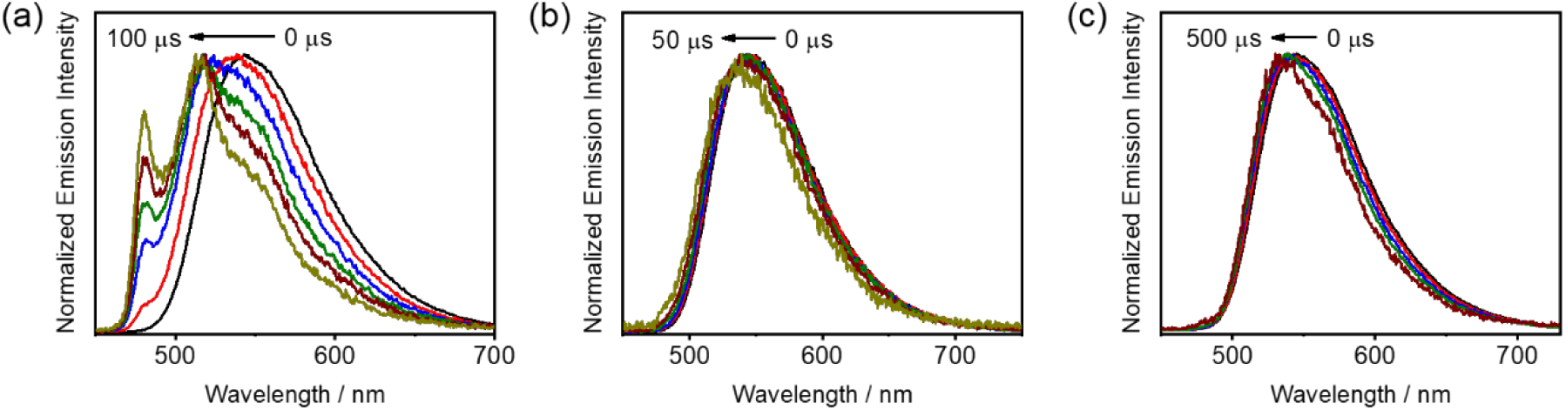
Time-resolved emission spectra of (a) **1**, (b) **2** and (c) **3** in toluene matrix at 77 K.

The presence of energetically close-lying triplet excited states is further supported by time-resolved emission studies in toluene matrix at 77 K (Figure 6). Upon increasing the delay time, the emission band shape changes from structureless to vibronic-structured for **1** as well as for **2** and **3** to a smaller extent (Figure 6), indicating a charge transfer character of the low-lying emissive triplet excited states in **1**–**3** and a more pronounced involvement of ^3^IL character of the emissive triplet excited state in **1** than those of **2** and **3**. Small blue spectral shifts of *ca*. 923 cm^–1^ (110 meV) for **1** at a delay time from 0 to 100 μs, *ca*. 328 cm^–1^ (40 meV) for **2** at a delay time from 0 to 50 μs and *ca*. 482 cm^–1^ (60 meV) for **3** at a delay time from 0 to 500 μs are found. The energy gap obtained is generally comparable to that obtained in variable-temperature emission and lifetime studies, supporting the TSDP properties in this class of gold(III) complexes.

### Transient Absorption Studies

**Figure 7:**
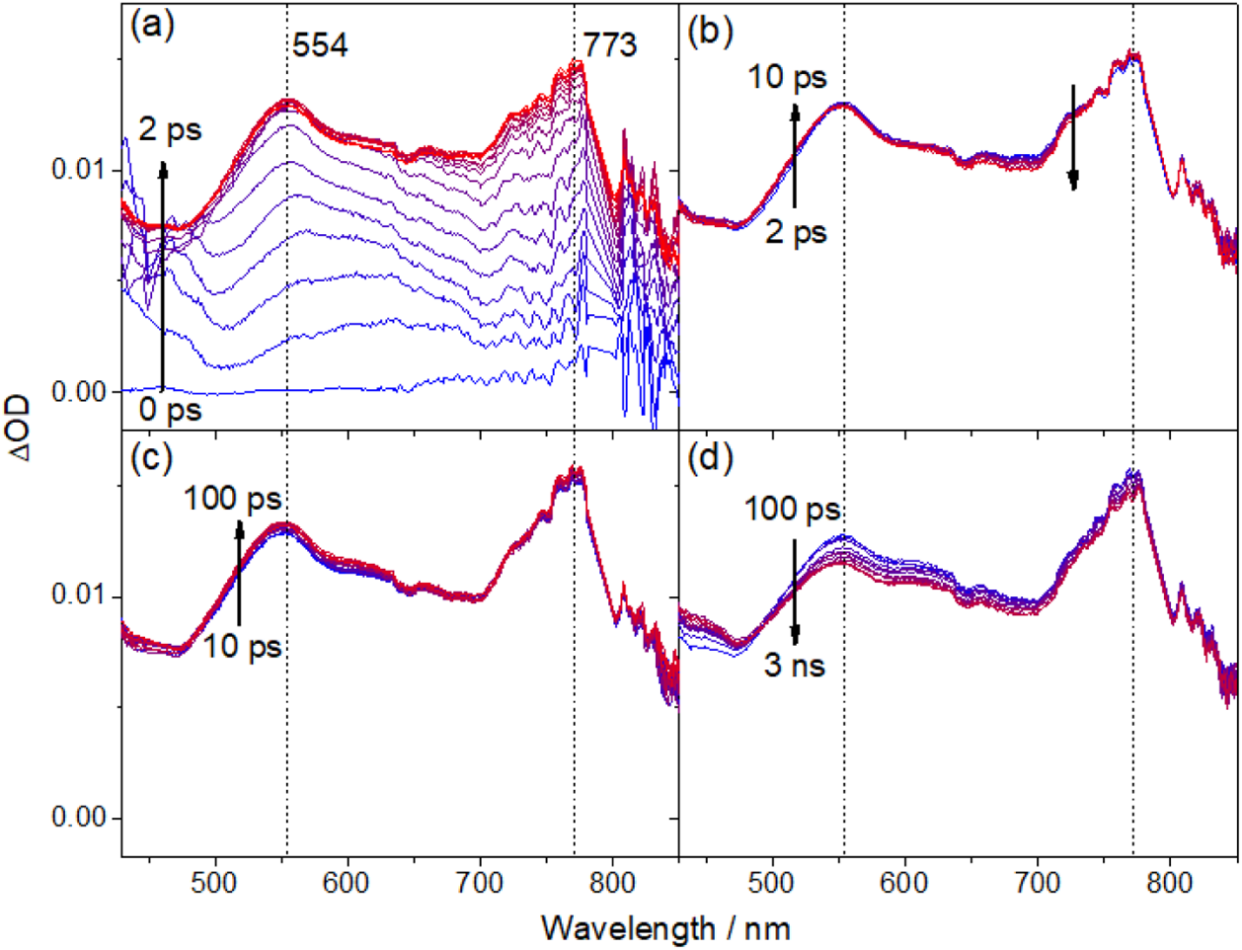
Selected fs-TA spectra of **1** in toluene solution at 298 K recorded at (a) 0–2, (b) 2–10, (c) 10–100 and (d) 100–3000 ps after 400 nm laser excitation.

To further elucidate the excited-state dynamics of this class of gold(III) complexes, femtosecond transient absorption (fs-TA) studies on the complexes in toluene solution at 293 K have been performed using a 400 nm fs laser pulse, and the fs-TA difference spectra measured at different pump–probe delay times for complexes **1**, **2** and **3** are illustrated in Figures 7, S14 and S15, respectively. Detailed analysis of the TA signals and their kinetics in the picosecond regime shows that the TA spectral changes of these complexes are similar, comprising four to five exponential components. To illustrate the excited-state dynamics associated with each component, selected TA spectra showing the major spectral changes associated with each component are shown in panels (a)–(d) of Figures 7, S14 and S15. Taking **1** as an example, the initial rise (τ_1_ ∼ 400 fs) shows broad absorption features peaking at *ca*. 556 and *ca*. 780 nm, which is partly masked by the laser peak at 800 nm (Figure 7a). The subsequent changes of the spectral features across different regions exhibit different kinetic profiles. As these absorption features are broad and tend to overlap, their kinetic analyses were conducted by selecting the far end of the spectral changes to minimize any interference from spectral changes with different kinetics. With this kinetic analysis approach, the intensity is found to decrease in the region of approximately 620–720 nm and increase in the 490–520 nm region, with time constants τ_2_ ≈ 4.0 ps and τ_3_ ≈ 7.0 ps, respectively. Such variation in the kinetic profile suggests that different photophysical processes occur simultaneously. A slower decay in the absorption feature at 550–710 nm (τ_4_, 39.4 ps) is subsequently observed with a minor spectral shift of the absorption band at 560 nm. Finally, these signals are found to decay with a time constant beyond the instrument limit (τ_5_ > 20 ns). Similar spectroscopic properties are observed in **2** and **3** (see Supporting Information). The initial rise can be ascribed to the ISC from an initially populated singlet excited state to a triplet state.^44,45^ The broad TA absorption at *ca*. 500–780 nm is similar to the absorptions of the radical anion of the cyclometalating C^C^N ligand and the radical cations of carbazole and PXO,^25,44^ suggesting the population of the LLCT excited state.

**Figure 8:**
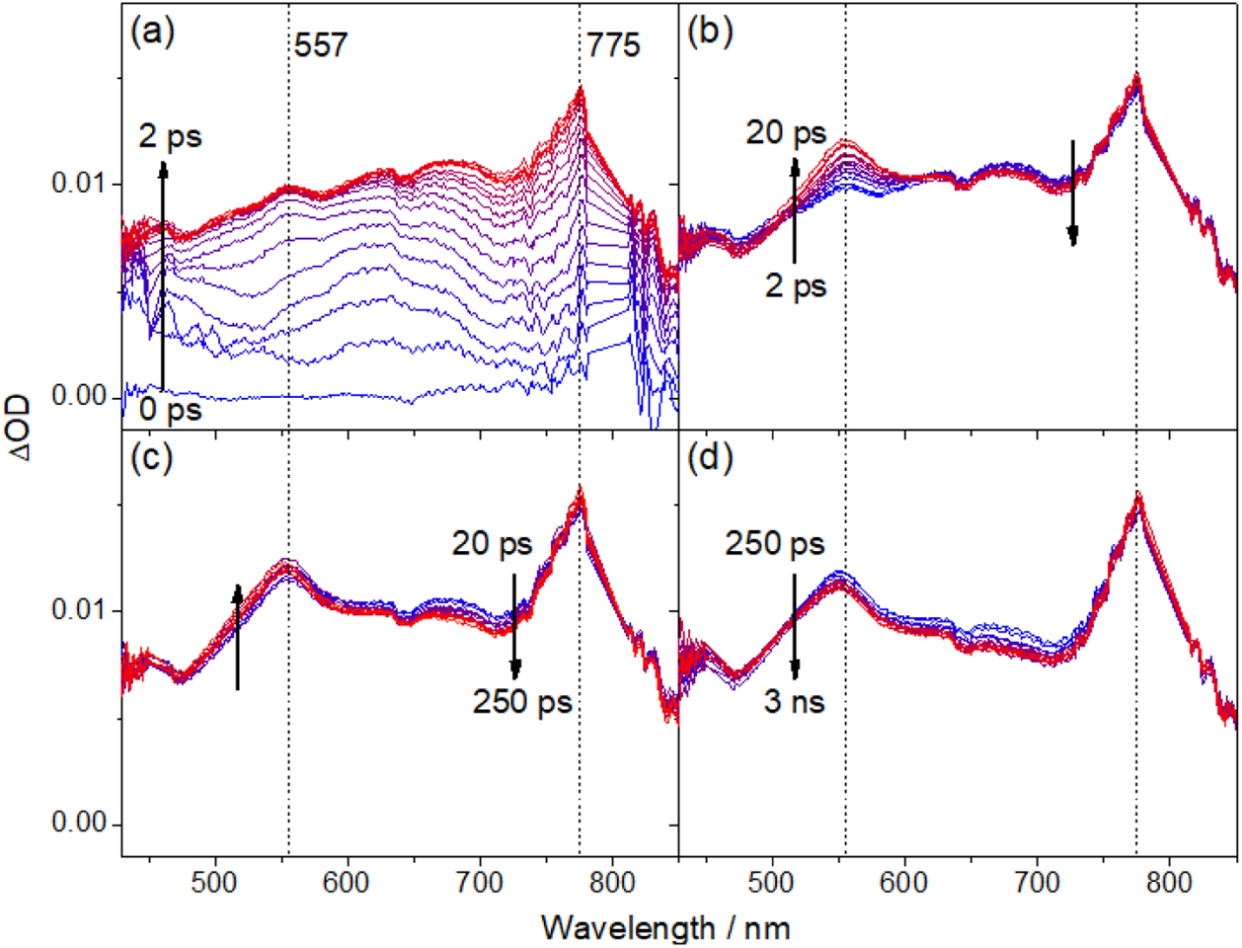
Selected fs-TA spectra of **1** in toluene solution at 188 K recorded at (a) 0–2, (b) 2–20, (c) 20–250, and (d) 250–3000 ps after 400 nm laser excitation.

**Figure 9:**
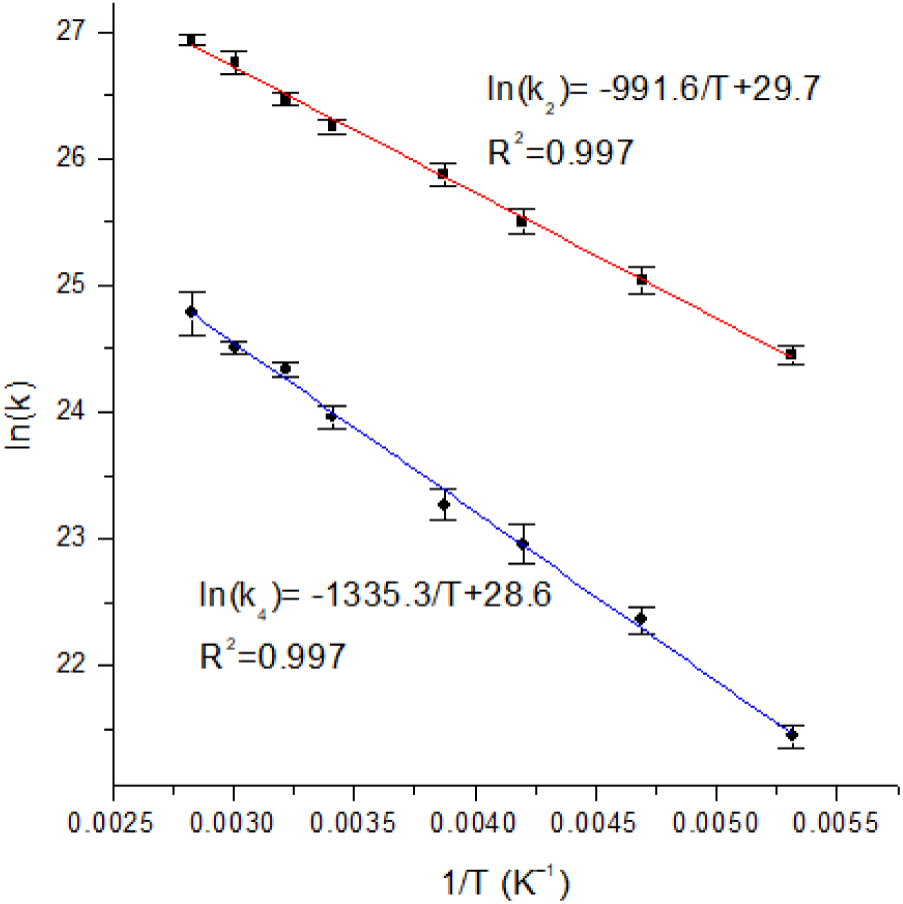
Arrhenius plots of *k*_2_ (τ_2_) and *k*_4_ (τ_4_) from the kinetic analysis of the transient absorption spectra of **1**. Among the processes observed in the ps regime, the processes associated with τ_2_ and τ_4_ show temperature dependence, and the calculated *E*_a_ for the processes are +8.2 ± 0.2 kJ mol^–1^ (85 ± 2 meV) and +11.1 ± 0.2 kJ mol^–1^ (115 ± 2 meV).

**Figure 10:**
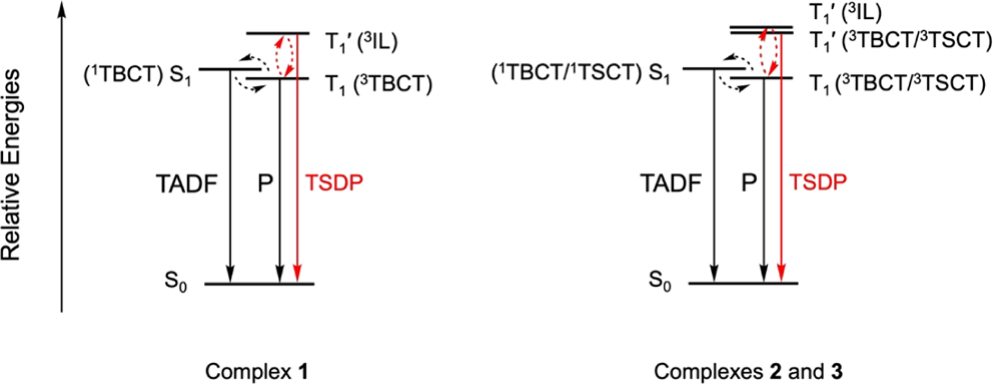
Plausible schematic diagram for emission processes of **1**–**3** in the solid state and the solid-state thin film. Here, P represents phosphorescence process.

To gain insights into the processes with τ_2_–τ_4_ subsequent to the initial ISC (τ_1_), the fs-TA spectra of **1**–**3** (Figures 7, 8 and S16–S35) at temperatures from 188 to 354 K have been studied. Similar to the variable-temperature TA spectroscopic studies of other gold(III) complexes,^20^ the ISC (τ_1_) is found to be rather insensitive to the change in temperature. By comparing the TA spectra of complex **1** at high and low temperatures (Figures 7, 8 and S16–S21), the kinetic profiles and distinctive absorption spectral changes associated with τ_2_ and τ_3_ become well-resolved. This is because τ_2_ (2.0–23.9 ps) increases with decreasing temperature, while τ_3_ (6.2–7.0 ps) is insensitive to temperature. In addition, the kinetics of the fourth process (τ_4_ = 17.3–487.4 ps) show a strong sensitivity to the change in temperature (Table S10). At a low temperature of 188 K, the differences in the time constants τ_1_–τ_4_ are more pronounced, allowing for clearer identification of the spectral changes in each component. Notably, the growth of the spectral feature peaking at about 550 nm, associated with the τ_2_ process, which is well-mixed with the spectral changes of τ_1_ and τ_3_ at room temperature, becomes distinguishable (Figure 8b). The relatively temperature-insensitive third process is tentatively assigned to vibrational cooling as it shows a time constant τ_3_ comparable to those reported for other gold(III) complexes.^20,44−47^ This assignment is consistent with the relative insensitivity of vibrational relaxation to temperature changes.^48−50^ The rate constants for the second (*k*_2_) and fourth (*k*_4_) processes obtained at different temperatures are used to construct the Arrhenius plots, and the activation energies [*E*_a(*k*2)_ and *E*_a(*k*4)_] are determined to be +8.2 kJ mol^–1^ (85 meV) and +11.1 kJ mol^–1^ (115 meV), respectively (Figure 9). However, it is important to note that the activation energy (*E*_a_) corresponding to the second rate constant (*k*_2_) cannot be accurately determined due to the overlap of two processes within similar time range and spectral region. Despite efforts to minimize the influence of the other component by determining the time constants at the extreme ends of the spectral features, precise determination of *k*_2_, and thus *E*_a(_*_k_*_2)_ remains challenging. Nevertheless, the positive *E*_a_ values indicate that they are thermally activated processes. As discussed above on the TADF–TSDP properties (Table 1), the photoluminescence of these complexes are possibly derived from a mixture of TADF and TSDP (Figure 10). Given the closer-lying energies between S_1_ and T_1_ and the rapid ISC, the thermally activated processes with rate constants of *k*_2_ and *k*_4_ are assigned to be RISC and RIC, respectively. The large absorption spectral changes associated with *k*_2_ process are consistent with the change of the spin multiplicity of the excited species in RISC and are previously observed in the transient absorption spectroscopic studies of related complexes.^20,45−47,51^ The good agreement between *E*_a(_*_k_*_4)_ and T_1_′–T_1_ energy gaps obtained from variable-temperature emission and lifetime studies, as well as time-resolved emission studies further support the assignment.

Similar TA spectral changes are observed for **2** and **3** as depicted in Figures S22–S35. However, these complexes exhibit slightly different kinetic profiles (Table S10), especially for the rapid processes following the initial rise corresponding to ISC. Unlike **1**, the temperature-independent vibrational cooling in fs-TA spectra cannot be identified in **2** and **3**. This is likely due to the overlapping of the spectral changes which is highly similar to the rates of temperature-dependent process (τ_2_: 2.2–17.9 ps). Apart from this, the overall similarity in the TA spectral changes and temperature-dependent kinetic profiles among these three complexes suggest that they undergo similar excited-state dynamics. The activation energies [*E*_a(*k*2)_ and *E*_a(*k*3)_], corresponding to RISC and RIC, are determined from the Arrhenius plots. For **2**, the *E*_a(*k*2)_ and *E*_a(*k*3)_ are found to be 7.1 kJ mol^–1^ (74 meV) and 9.3 kJ mol^–1^ (96 meV) while they are 5.8 kJ mol^–1^ (60 meV) and 10.2 kJ mol^–1^ (106 meV) for **3**, respectively (Figures S60 and S61). It is important to mention that the apparent rate constants *k*_2_ determined for **2** and **3** may not accurately reflect the actual temperature-dependent process because they are likely affected by the mixing of the spectral changes by the temperature-insensitive processes. Consequently, the *E*_a(*k*2)_ determined for **2** and **3** might deviate from the actual energy gap (Δ*E*_S1–T1_) in these complexes. In contrast, the lifetimes τ_3_ of **2** and **3** can be determined with good certainty, as they are much longer than the initial rapid processes and notably shorter than the longest process observed in the picosecond to sub-nanosecond regime. The *E*_a(*k*3)_ values for **2** and **3** align well with the T_1_′–T_1_ energy gaps estimated from variable-temperature emission and lifetime studies, as well as their time-resolved emission properties.

### Electrochemical Studies

The electrochemical properties of **1**–**3** have been investigated by cyclic voltammetry in tetrahydrofuran solution. The cyclic voltammograms are shown in Figure S63 and the corresponding electrochemical data are summarized in Table S11. Generally, all the complexes show one irreversible reduction couple and one or two quasi-reversible oxidation couples. With reference to the previously reported structurally related C^C^N tridentate gold(III) complexes,^25−27^ the irreversible reduction couple at −1.55 V to −1.61 V vs saturated calomel electrode (SCE) for **1**–**3** has been attributed to one electron reduction of the C^C^N tridentate ligand. The cathodic shift in the reduction potential from −1.55 V for **1** to −1.57 V for **2** and −1.61 V for **3** is likely due to stronger D–A CT and π–π interactions between the cyclometalating C^C^N pincer ligand and the lateral arylamine moiety on the carbazolyl ligand, such that the π*(C^C^N) orbital becomes destabilized in the order of **3** > **2** > **1**.^17^ For the oxidation, the potential for the first quasi-reversible oxidation is less sensitive to the increase in electron-donating strength of the lateral arylamine moiety on the carbazolyl ligand from acridine (DMAC, +0.85 V vs SCE) to phenoxazine (PXZ, +0.84 V vs SCE) to double oxygen-bridged triphenylamine (DPXO, +0.80 V vs SCE). Therefore, the first quasi-reversible oxidation couple has been reasonably assigned as one-electron oxidation of the carbazolyl ligand. The rather low sensitivity of the potential for oxidation to the arylamine moiety is ascribed to the rather twisted dihedral angle between the arylamine moiety and the carbazolyl ligand, such that the π(carbazolyl) orbital is only slightly perturbed.

### Computational Studies

**Figure 11:**
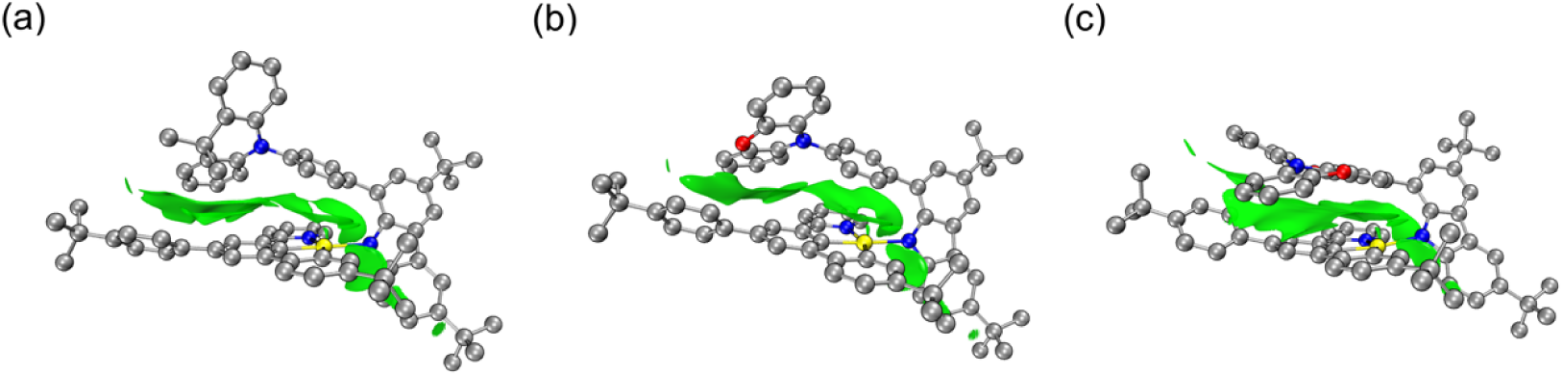
Isosurfaces of the noncovalent interaction for (a) **1**, (b) **2** and (c) **3** in toluene solution. Hydrogen atoms are removed for clarity.

To gain deeper insights into the electronic structures and the nature of absorption and emission origins, density functional theory (DFT) and time-dependent DFT (TDDFT) calculations have been performed on **1**–**3**. The ground state (S_0_) geometries and isosurfaces of noncovalent interactions (NCIs)^52^ are provided in Figure 11. The almost planar DMAC in **1** in the bulk crystal state (Figure S64a) rearranges to an open-book structure in toluene solution with a dihedral angle of 145.5° (Figure S64b). The distortion of DMAC might be driven by the folding of the phenyl ring toward the C^C^N ligand, thereby achieving a better alignment with the C^C^N ligand. The orientation of the carbazolyl ligand and the lateral bridging phenyl ring also exhibits a more parallel alignment. Consequently, results from the NCI analysis suggest that the C–H···π interaction-dominated weak interactions between the DMAC and C^C^N ligand in the crystal state (Figure S65a) are switched to π–π interactions in toluene solution (Figure 11a). The transition of weak interactions from C–H···π interactions in the crystal state (Figure S65b) to π–π interactions in toluene solution (Figure 11b) has been also observed between the DPXO and the cyclometalating C^C^N ligand in complex **2**. NCI isosurface of **3** is smooth and continuous, while discontinuity of the isosurfaces is found in **1** and **2**, which is in line with the extent of parallel stacking between the lateral arylamine and the cyclometalating C^C^N acceptor in an order of **3** > **2** > **1** (Figure 11). The NCI analysis also displays C–H···π interaction between the cyclometalating C^C^N and carbazolyl auxiliary ligands for all the complexes studied.

**Figure 12:**
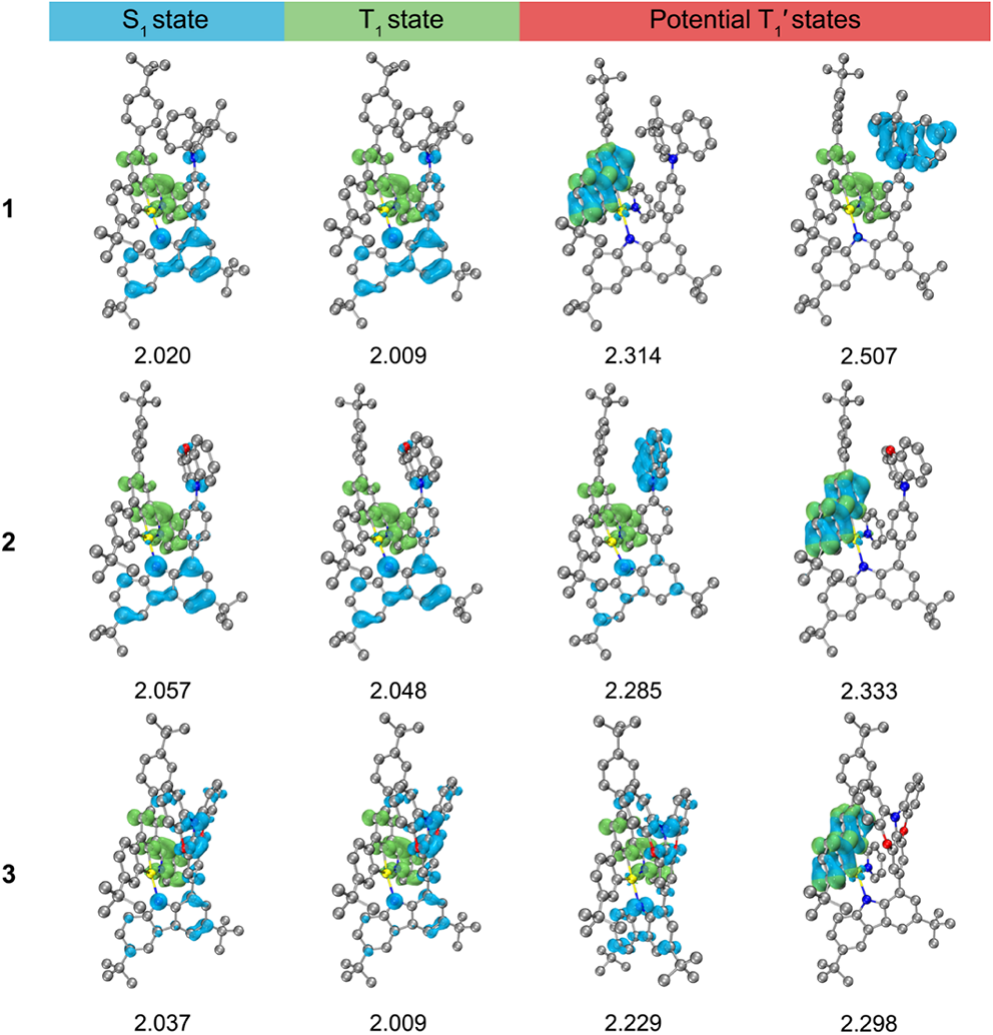
Distributions of the hole (blue) and electron (lime) for S_1_, T_1_, and potential T_1_′ states of **1**–**3** computed at their respective optimized structures. Computed excited state energy levels (eV) with respect to the optimized S_0_ state are provided.

To investigate the nature of emissive states, geometries of the S_1_, T_1_ and potential T_1_′ states have been optimized by TDDFT method and their distributions of electron and hole densities are shown in Figure 12. The percentage contributions of TBCT and TSCT characters of emissive states are further quantified using the interfragment charge transfer (IFCT)^47^ analysis (Figure S73 and Table S13). For both S_1_ and T_1_ states in **1**–**3**, the hole density distributes over the donor segment and the electron is localized on the cyclometalating C^C^N ligand. Such negligible hole–electron spatial overlap leads to an extremely small Δ*E*_S1–T1_ values of <30 meV, which favors TADF process. Results from IFCT analysis indicate that S_1_ and T_1_ states in **1** and **2** are almost pure TB-LLCT in nature (TBCT character ∼70%), whereas those in **3** contain a considerable percentage contribution from TSCT character (>50%). Notably, the electron-donating strength of the lateral arylamine and the spatial alignment between the lateral arylamine and the cyclometalating C^C^N ligand demonstrate significant impacts on the energy level of the higher-lying T_1_′ states. Due to the weak donor strength of DMAC, the pure ^3^TSCT state of **1** is energetically destabilized by over 190 meV relative to the ^3^IL state localized on C^C^N ligand and by around 500 meV relative to the pure ^3^TBCT (T_1_) state. In sharp contrast to **1**, T_1_′ state dominated by TSCT character in **2** is energetically lower-lying than the ^3^IL(C^C^N) state by 48 meV. The stabilization of ^3^TSCT state from **1** to **2** could be attributed to the better parallel alignment between PXO and the cyclometalating C^C^N ligand and the stronger donor strength of PXO over DMAC. For **3** with a cofacial spatial alignment and the strongest π–π interaction between DPXO and the cyclometalating C^C^N ligand, the TSCT and TBCT characters are considerably mixed in both T_1_ and the relatively lower-lying T_1_′ state, and the energy difference between T_1_′ (mixed TSCT/TBCT character) state and ^3^IL(C^C^N) state is computed to be 69 meV. The computed Δ*E*_S1–T1_ (≤28 meV) and Δ*E*_T1′–T1_ (≤305 meV) values (Table S14) of **1**–**3** are in the same order of magnitude as the values determined experimentally from the variable-temperature fs-TA studies (Δ*E*_S1–T1_ ≤ 85 meV and Δ*E*_T1′–T1_ ≤ 115 meV).^53^ These computational results on triplet excited states support that all the complexes are TSDP active given the presence of the T_1_′ excited states that are close-lying to the T_1_ state, i.e., ^3^IL(C^C^N) state in **1**, ^3^TSCT and ^3^IL(C^C^N) states in **2**, and mixed ^3^TSCT/^3^TBCT and ^3^IL(C^C^N) states in **3**, and the TSDP efficiency can possibly be enhanced by altering both the energy level and the nature of these close-lying triplet excited states. Hence, the emission origin of **1**–**3** is assigned as originating from T_1_ and T_1_′ states, involving a mixture of metal-perturbed ^3^IL [π→π*(C^C^N)], ^3^TB-LLCT [π(carbazolyl ligand)→π*(C^C^N)] and ^3^TS-LLCT [π(lateral arylamine)→π*(C^C^N)] excited states.

### OLED Fabrication and Characterization

**Figure 13:**
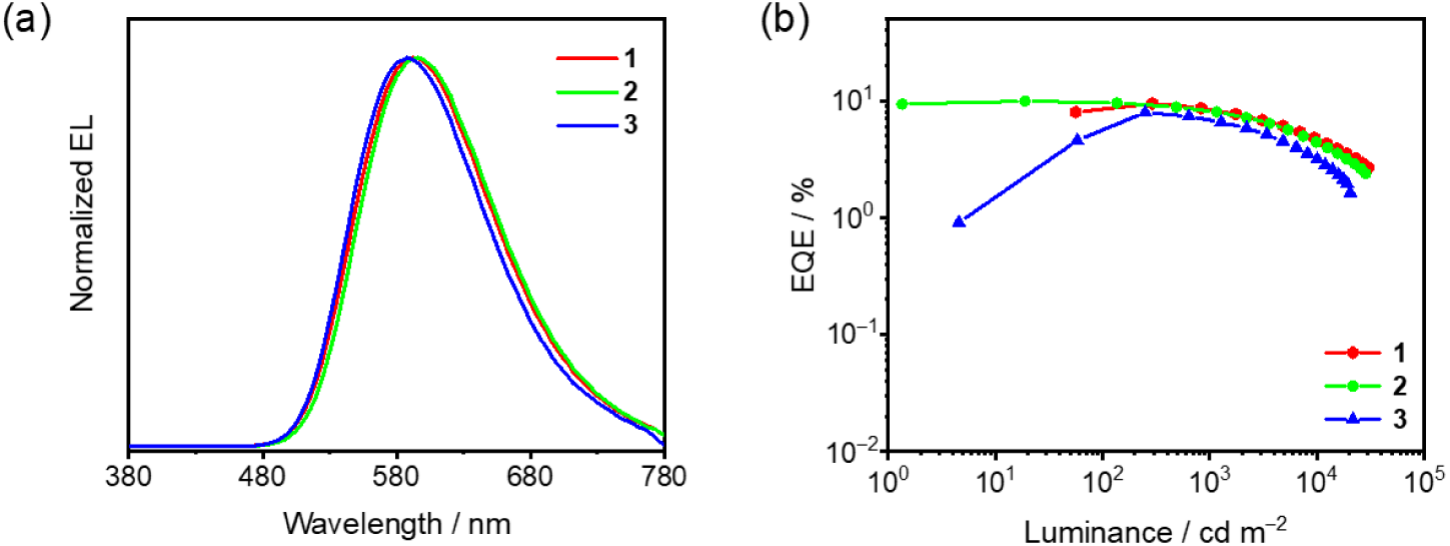
(a) Normalized EL spectra and (b) EQEs of the vacuum-deposited OLEDs based on 14 v/v% **1**–**3** doped in *m*-CBP at different concentrations.

Vacuum-deposited OLEDs based on **1**–**3** have been fabricated to investigate their electroluminescence (EL) properties, as shown in Figure 13. Consistent with the PL behaviors in the solid-state thin films, all the devices made with **1**–**3** exhibit Gaussian-shaped EL bands with similar emission energies. In addition, unlike other square-planar metal complexes, the EL spectra are relatively insensitive to the increase in dopant concentration. Notably, the EL spectra show small shifts of 349 cm^–1^ for **1**, 582 cm^–1^ for **2** and 354 cm^–1^ for **3** with increasing dopant concentrations from 2 v/v% to 14 v/v% (Figure S74). This could be ascribed to the incorporation of arylamine at the lateral position of the carbazolyl auxiliary ligand, in which the rigidification via the D–A CT and π–π interactions between the lateral arylamine donor and the cyclometalating C^C^N acceptor ligand has resulted in steric protection of the metal center by the lateral donor group, hence suppressing intermolecular interactions and thus excimeric emission. Orange-emitting OLEDs with respectable current efficiencies up to 25.7 cd A^–1^, corresponding to maxima EQEs of up to 10.9%, with moderate efficiency roll-offs in the range of 12–32% at luminance of 1000 cd m^–2^ have been demonstrated (Table S17).^54^

**Figure 14:**
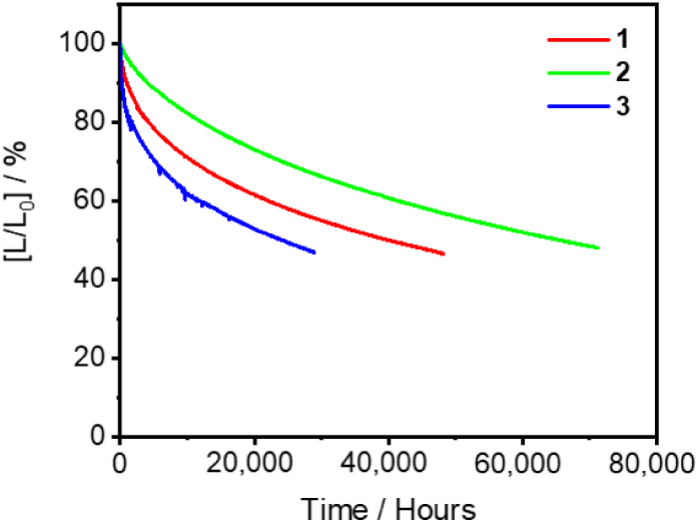
Relative luminescence (*L*/*L*_0_) of the vacuum-deposited OLEDs based on 11 v/v % **1**–**3** as a function of time under a constant driving current density of 20 mA cm^–2^.

The operational stabilities of these vacuum-deposited devices have also been tested by accelerated testing under a constant driving current density of 20 mA cm^–2^. The relative luminance (*L*/*L*_0_) of the OLEDs based on **1**–**3** as a function of time are illustrated in Figure 14 and the corresponding device lifetime data are summarized in Table S18. Device operational half-lifetimes (LT_50_), defined as the operational lifetime at 50% of initial brightness (*L*_0_), are determined to be 141 h for **1**, 236 h for **2** and 135 h for **3**. These correspond to the estimated LT_50_ of 39,873 h for **1**, 65,314 h for **2** and 24,007 h for **3** projected at an initial luminance of 100 cd m^–2^. The better operational stability in **2** than **1** and **3** could be mainly explained by the discrepancies in the emission lifetimes in doped thin films (i.e., **2** < **1** < **3**) (Table 1). The reduced triplet exciton lifetime can suppress the formation of high-energy singlet excitons via triplet–triplet annihilation, hence suppressing the degradation of organic materials. It is worth noting that the operational lifetimes of the present devices made with **1**–**3** with a lateral arylamine on the carbazolyl auxiliary ligand are one to two orders of magnitude longer than those of our previously reported structurally related carbazolyl gold(III) analogues with a *meta*- or *para*-substituted arylamine.^27^ These are likely due to the synergistic effects of the much shorter exciton lifetimes and the higher thermal stabilities of **1**–**3**, as revealed by TGA (Figure S1 and Table S1). It should be highlighted that such long device operational lifetimes, to the best of our knowledge, represent the most stable orange-emitting gold(III)-based OLEDs reported in the literature so far. Table S19 summarizes the key parameters of the state-of-the-art OLEDs based on TADF/TSDP emitters with similar emission colors.

## Conclusion

In conclusion, a new class of TSCT carbazolylgold(III) C^C^N complexes with unique TADF–TSDP properties has been developed by introducing a rigid lateral arylamine on the carbazolyl auxiliary ligand. The significant twisting between the cyclometalating C^C^N pincer ligand and the carbazolyl auxiliary ligand results in a negligible hole–electron overlap and thus a rather small Δ*E*_S1–T1_ which enables TADF properties of this class of gold(III) complexes. Both experimental transient absorption and emission studies and theoretical calculations reveal that the intramolecular through-space electronic coupling between the cyclometalating C^C^N ligand and the lateral arylamine in close proximity enables the formation of a relatively low-lying ^3^TSCT state, which can be further stabilized by strengthening such through-space electronic coupling through their better alignment with extended planarity and the strengthening of the electron-donating ability of the lateral arylamine (DMAC (**1**) < PXO (**2**) < DPXO (**3**)). The rapid RIC process has also been supported by the relatively smaller *E*_a_ determined by temperature-dependent fs-TA studies. An enhanced contribution of the TSCT character into ^3^TBCT (T_1_) state has also been suggested by computational calculations, which is favorable for RIC from T_1_ state to the energy close-lying T_1_′ state with a predominant TSCT character. Orange-emitting vacuum-deposited OLEDs with respectable maximum EQEs of 9.0–10.9% with small efficiency roll-offs have been achieved; more importantly, long operational stabilities with LT_50_ of up to 65,314 h have been realized. This work not only demonstrates the unique TADF–TSDP emission mechanism in the C^C^N gold(III) complexes, but also provides a new design approach to enrich the structural diversity of TADF–TSDP complexes, shedding light on the deeper understanding of the excited-state dynamics for optimal design of high-performance emitters.

## Supporting Information Available

Crystal and structure determination data; computational details; steady-state and time-resolved absorption and emission spectra; electrochemical data; OLED data (PDF)
