# Supplementary material for: Motivation on Intramolecular Through-Space Charge Transfer for the Realization of Thermally Activated Delayed Fluorescence (TADF)–Thermally Stimulated Delayed Phosphorescence (TSDP) in C^C^N Gold(III) Complexes and Their Applications in Organic Light-Emitting Devices
Source: J Am Chem Soc. 2025 Mar 30;147(14):12092–104. doi: 10.1021/jacs.5c00121 (PMC11987025; doi:10.1021/jacs.5c00121)
Supplement: Supplementary file 1 — ja5c00121_si_001.pdf [file ja5c00121_si_001.pdf]

Supporting Information

Motivation on Intramolecular Through-Space Charge Transfer for the Realization of Thermally Activated Delayed Fluorescence (TADF)-Thermally Stimulated Delayed Phosphorescence (TSDP) in C^C^N Gold(III) Complexes and Their Applications in Organic Light-Emitting Devices

Panpan Li,[‡ab] Ziyong Chen,[‡a] Ming-Yi Leung,[ab] Shiu-Lun Lai,[a] Shun-Cheung Cheng,[c] Wing-Kei Kwok,[ab] Chi-Chiu Ko,[c] Mei-Yee Chan,[ab] and Vivian Wing-Wah Yam*[ab]

[a] Institute of Molecular Functional Materials and Department of Chemistry, The University of Hong Kong, Pokfulam Road, Hong Kong, P. R. China
Fax: +(852) 2857-1586; Tel: +(852) 3917-2153

* E-mail: wwyam@hku.hk

[b] Hong Kong Quantum AI Lab Limited, 17 Science Park West Avenue, Pak Shek Kok, Hong Kong, P. R. China

[c] Department of Chemistry, City University of Hong Kong, Tat Chee Avenue, Kowloon Tong, Hong Kong. P. R. China

‡ Both authors contributed equally to this work.

Table of Contents

Experimental Details………………………………………………… S3

Synthesis and Characterization…………………………………… S5

Thermogravimetric Analysis……………………………………… S9

X-Ray Crystal Structures…………………………………………. S10

Photophysical Properties………………………………………….. S16

Variable-Temperature Emission Studies..……………………...…. S19

Transient Absorption Studies…………………………………….. S26

Electrochemical Studies…………………………………………... S54

Computational Studies……………………………………………. S55

OLED Fabrication and Characterization………………………… S66

NMR Spectra……………………………………………………... S100

References………………………………………………………… S103

Experimental Details

Materials and Reagents. All solvents were purified and distilled using standard procedures before use. All other reagents were of analytical grade and were used without further purification. Tetra-*n*-butylammonium hexafluorophosphate (Aldrich, 98 %, $^{n}Bu_4NPF_6$) was recrystallized for no less than three times from hot absolute ethanol prior to use.

Physical Measurements and Instrumentation.

^{1}H NMR and ^{13}C NMR spectroscopy were performed using a Bruker DRX 500 NMR spectrometer at 298 K with chemical shifts (δ, ppm) relative to tetramethylsilane (Me_4Si) for the ^{1}H NMR and ^{13}C NMR spectra. High resolution electrospray ionization (ESI) mass spectroscopy was performed using a Bruker MaXis II Ultrahigh-Resolution Time-of-Flight Mass Spectrometer. The UV−vis absorption spectra were recorded on a Varian Cary 50 spectrophotometer equipped with a Xenon flash lamp. Steady-state emission spectra were recorded using an Edinburgh Instruments FS5 spectrofluorometer. Toluene solutions of **1**–**3** (10^{-5} M) were prepared to investigate the steady-state absorption and photoluminescence (PL) characteristics in the solution state.

The femtosecond transient absorption experiments were conducted using the HELIOS fs-TA spectrometer designed by Ultrafast Systems. The pump pulses with an excitation wavelength of 400 nm were produced by frequency doubling of an 800-nm laser pulse generated by a femtosecond laser system with Ti:Sapphire amplifier (Coherent Libra-HE). The pulse width of the fs laser pulse is approximately 150 fs. A white-light continuum, as the probe light source, was produced by focusing the 800-nm light onto a sapphire crystal plate. The pump and white-light continuum probe beams were focused and overlapped on the sample solution in a 2-mm cuvette. The time delay (0–3000 ps) between the pump and probe beam was controlled by an optical delay line. The probe beam passed through the sample was focused on an optical fiber connecting to a spectrograph with a CCD detector with PC plugin controller, fully operated by

Surface Xplorer software. The kinetic analysis of the fs-TA data was performed to resolve the spectral changes associated with distinct photophysical processes (Figures S36–S59). For the kinetic analysis of **1**, the shortest-lived (initial rise) and longest-lived components were determined by global fitting across the entire wavelength range. For intermediate components with potential overlap, fitting was performed with the wavelength ranges at the extreme ends of the spectral features, as specified in the footnotes of Table S10, to minimize the influence of the overlapping components. For the kinetic analysis of **2** and **3**, where different photophysical processes are well-separated, the kinetics were determined by global fitting across the entire wavelength range. The initial estimates for τ_2 and τ_3 were based on the significant spectral changes observed, as specified in the footnotes of Table S10. This approach was used to minimize the influence of overlapping components. The picosecond transient absorption spectra were recorded on a Picosecond Transient Absorption Spectroscopy System (UNISOKU Co., Ltd.) at ambient temperature. The excitation source was the OPA output of a picosecond mode-locked Nd:YAG laser (EKSPLA PT403 Tunable Wavelength Picosecond Laser), and the probe light source was a picosecond Supercontinuum Light Source. The absorption kinetics were detected by amplified photodiodes and recorded on digital oscilloscope (200 MHz, 12 bit) with PC plugin controller, fully operated by UNISPEC software. Solution samples were degassed by bubbling with argon.

Solutions were degassed by using a high vacuum line in a two-compartment cell with four freeze-pump-thaw cycles. The optical dilute method developed by Demas and Crosby[1] was applied to determine the relative luminescence quantum yields of solutions with the use of quinine sulfate in 0.5 M sulfuric acid (Φ_{em} = 0.546, λ_{ex} = 365 nm) at 298 K as the standard.[2] Excited-state lifetimes of solutions and thin films were measured on a Quantaurus-Tau C11367-34 fluorescence lifetime spectrometer with the 340 nm excitation source. Time-resolved PL spectra in toluene matrix at 77 K were recorded on an Edinburgh Instruments LP980 Spectrometer with an Oxford Instrument OptistatDN2

cryostat. Thin films of 5 wt% **1**–**3** doped into 1,3-bis(carbazol-9-yl)benzene (mCP) were prepared by spin-coating a 10 mg cm^{-3} chloroform solution onto quartz substrates at spinning speed of 2000 rpm. The thin film was placed in a holder inside an Oxford Instrument OptistatDN2 cryostat and maintained at the desired temperature until equilibrium was reached before recording the decay curves at variable temperatures. Absolute photoluminescence quantum yields (PLQYs) of thin films were measured on Hamamatsu C9920-03 Absolute PLQY Measurement System under an excitation at 300 nm. Cyclic voltammetric (CV) measurements were performed by using a CH Instruments, Inc. model CHI 620A electrochemical analyzer. Electrochemical measurements were performed in tetrahydrofuran (THF) solutions with 0.1 mol dm^{-3} $^{n}Bu_4NPF_6$ used as supporting electrolyte at room temperature. The reference electrode was a Ag/$AgNO_3$ (0.1 mol dm^{-3} in acetonitrile) electrode, and the working electrode was a glassy carbon electrode (CH Instruments, Inc.) with a platinum wire as the counter electrode. The ferrocenium/ferrocene couple (Fc^+/Fc) was used as the internal reference. All solutions for electrochemical studies were deaerated with prepurified argon gas prior to measurements.

Synthesis and Characterization

[Au{4-^{t}BuC^C(4-^{t}BuC_6H_4)^N}{(1-(N-PhDMAC)-3,6-^{t}BuDTC}] (**1**). NaH (9.6 mg, 0.24 mmol) was added into the solution of 10-(4-(3,6-di-*tert*-butyl-9*H*-carbazol-1-yl)phenyl)-9,9-dimethyl-9,10-dihydroacridine (L1) (90 mg, 0.16 mmol) in anhydrous tetrahydrofuran (10 ml) and then stirred for 10 mins. [Au{4-^{t}BuC^C(4-^{t}BuC_6H_4)^N}Cl] (100 mg, 0.16 mmol) in anhydrous tetrahydrofuran (10 mL) was then subsequently transferred into the above solution, and the resultant reaction mixture was left to stir at room temperature for overnight. After removing the solvent, the crude product was purified by column chromatography on aluminum dioxide to afford a yellow solid. Yield: 66 mg, 56 %. ^{1}H NMR (500 MHz, CD_2Cl_2, 298 K, relative to Me_4Si, δ/ppm): δ 8.75 (d, J = 8.0 Hz, 1H, pyridyl proton), 8.30 (s, 1H, carbazolyl proton), 8.25 (s, 1H, carbazolyl proton), 8.05 (t, J = 8.0 Hz, 1H, pyridyl proton), 7.95 (d, J = 8.0 Hz, 1H,

pyridyl proton), 7.85 (d, J = 8.0 Hz, 2H, $–C_6H_4–$), 7.54–7.59 (m, 4H, $–C_6H_4–$), 7.53 (s, 1H, $–C_6H_2–$), 7.51 (s, 1H, $–C_6H_2–$), 7.48 (s, 1H, carbazolyl proton), 7.46 (d, J = 8.5 Hz, 1H, carbazolyl proton), 7.44 (t, J = 8.0 Hz, 1H, pyridyl proton), 7.30–7.33 (m, 3H, $–C_6H_4–$ and carbazolyl proton), 7.22 (d, J = 8.0 Hz, 1H, $–C_6H_3–$), 7.02 (d, J = 8.5 Hz, 1H, $–C_6H_3–$), 6.94 (d, J = 8.5 Hz, 2H, DMAC protons), 6.73 (t, J = 8.5 Hz, 2H, DMAC protons), 6.60 (t, J = 8.5 Hz, 2H, DMAC protons), 6.15 (s, 1H, $–C_6H_3–$), 6.01 (d, J = 8.5 Hz, 2H, DMAC protons), 1.55 (s, 9H, $–^t$Bu), 1.50 (s, 6H, $–CH_3$), 1.43 (s, 9H, $–^t$Bu), 1.41 (s, 9H, $–^t$Bu), 0.86 (s, 9H, $–^t$Bu). ^{13}C{H} NMR (150 MHz, CD_2Cl_2, relative to Me_4Si, δ/ppm): δ 164.78, 164.49, 151.68, 151.58, 151.55, 151.27, 149.33, 149.13, 148.35, 145.99, 142.58, 142.23, 141.75, 141.38, 141.08, 140.50, 139.91, 139.66, 138.73, 132.24, 131.25, 130.14, 129.91, 127.89, 127.27, 126.67, 126.53, 126.23, 125.77, 125.24, 125.02, 124.58, 124.36, 122.45, 122.38, 121.61, 120.99, 120.82, 120.49, 116.17, 116.05, 114.19, 113.81, 36.08, 34.95, 34.90, 34.85, 34.77, 32.43, 32.32, 31.49, 31.26, 30.96, 30.06. HRMS (positive ESI) calcd for $C_{72}H_{72}AuN_3$: m/z = 1175.5392 $[M]^+$; found 1175.5353 $[M]^+$. Elemental analyses: Found (%): C, 73.22; H, 6.23; N, 3.34. Calcd for $C_{72}H_{72}AuN_3$: C, 73.51; H, 6.17; N, 3.57.

[Au{4-^{t}BuC^C(4-^{t}BuC_6H_4)^N}{(1-(N-Ph-PXO)-3,6-^{t}BuDTC}] (**2**). The procedure was similar to that used to prepare **1**, except 1-(4-(9*H*-xanthen-9-yl)phenyl)-3,6-di-*tert*-butyl-9*H*-carbazole (L2) (86 mg, 0.16 mmol) was used in place of L1. The product was isolated as a yellow solid. Yield: 60 mg, 52 %. ^{1}H NMR (500 MHz, CD_2Cl_2, 298 K, relative to Me_4Si, δ/ppm): δ 8.73 (d, J = 8.0 Hz, 1H, pyridyl proton), 8.29 (s, 1H, carbazolyl proton), 8.25 (s, 1H, carbazolyl proton), 8.08 (t, J = 8.0 Hz, 1H, pyridyl proton), 7.98 (d, J = 8.0 Hz, 1H, pyridyl proton), 7.78 (d, J = 8.5 Hz, 2H, $–C_6H_4–$), 7.52–7.54 (m, 5H, $–C_6H_2–$, $–C_6H_3–$ and $–C_6H_4–$), 7.43–7.48 (m, 4H, $–C_6H_4–$, pyridyl and carbazolyl protons), 7.31 (d, J = 8.5 Hz, 1H, carbazolyl proton), 7.21 (d, 1H, $–C_6H_3–$), 7.02 (d, J = 8.5 Hz, 1H, $–C_6H_3–$), 6.93 (d, J = 8.0 Hz, 2H, $–C_6H_4–$), 6.53 (d, J = 7.5 Hz, 2H, PXO protons), 6.46 (t, J = 7.5 Hz, 2H, PXO protons), 6.28 (t, J = 7.5 Hz, 2H, PXO protons), 6.08 (s, 1H, $–C_6H_3–$), 5.63 (d, J = 7.5 Hz, 2H, PXO protons),

1.55 (s, 9H, –^{t}Bu), 1.45 (s, 9H, –^{t}Bu), 1.40 (s, 9H, –^{t}Bu), 0.85 (s, 9H, –^{t}Bu). ^{13}C{H} NMR (150 MHz, CD_2Cl_2, relative to Me_4Si, δ/ppm): δ 164.78, 164.31, 151.61, 151.59, 151.56, 151.31, 149.31, 149.16, 148.37, 146.03, 144.01, 142.97, 142.23, 141.84, 141.38, 140.51, 139.96, 138.65, 137.24, 134.63, 132.56, 130.72, 129.95, 127.88, 127.27, 126.44, 126.25, 125.77, 125.08, 124.61, 124.20, 123.78, 122.54, 122.33, 121.58, 121.21, 121.07, 120.81, 116.26, 116.10, 115.34, 113.79, 113.38, 34.97, 34.91, 34.88, 34.79, 32.44, 32.35, 31.51, 30.98. HRMS (positive ESI) calcd for $C_{69}H_{67}AuN_3O$: *m*/*z* = 1150.4950 [M+H]$^+$; found 1150.4944 [M+H]$^+$. Elemental analyses: Found (%): C, 71.90; H, 5.84; N, 3.46. Calcd for $C_{69}H_{66}AuN_3O$: C, 72.05; H, 5.78; N, 3.65.

[Au{4-^{t}BuC^C(4-^{t}BuC_6H_4)^N}{(1-(N-Ph-DPXO)-3,6-^{t}BuDTC}] (**3**). The procedure was similar to that used to prepare **1**, except 3,6-di-*tert*-butyl-1-(13b*H*-chromeno[2,3,4-*kl*]xanthen-7-yl)-9H-carbazole (L3) (88 mg, 0.16 mmol) was used in place of L1. The product was isolated as a yellow solid. Yield: 58 mg, 50 %. ^{1}H NMR (500 MHz, CD_2Cl_2, 298 K, relative to Me_4Si, δ/ppm): δ 8.32 (d, *J* = 8.0 Hz, 1H, pyridyl proton), 8.24 (s, 1H, carbazolyl proton), 8.22 (s, 1H, carbazolyl proton), 7.93 (t, *J* = 7.5 Hz, 1H, pyridyl proton), 7.64 (d, *J* = 8.0 Hz, 1H, pyridyl proton), 7.53 (d, *J* = 8.5 Hz, 1H, carbazolyl proton), 7.46 (d, *J* = 8.5 Hz, 2H, –C_6H_4–), 7.37 (d, *J* = 8.5 Hz, 1H, –C_6H_3–), 7.34 (t, *J* = 7.5 Hz, 1H, pyridyl proton), 7.26 (2H, *J* = 8.5 Hz, 2H, –C_6H_4–), 7.22 (s, 1H, carbazolyl proton), 7.19 (s, 1H, –C_6H_4–), 7.07 (s, 1H, –C_6H_4–), 7.00–7.05 (m, 2H, –C_6H_3– and carbazolyl proton), 6.92 (d, *J* = 8.0 Hz, 2H, DPXO protons), 6.82 (t, *J* = 8.0 Hz, 2H, DPXO protons), 6.71 (m, 2H, DPXO protons), 6.50 (d, *J* = 8.0 Hz, 2H, DPXO protons), 6.27 (s, 2H, DPXO protons), 6.05 (s, 1H, –C_6H_3–), 1.48 (s, 9H, –^{t}Bu), 1.47 (s, 9H, –^{t}Bu), 1.40 (s, 9H, –^{t}Bu), 0.85 (s, 9H, –^{t}Bu). ^{13}C{H} NMR (150 MHz, CD_2Cl_2, relative to Me_4Si, δ/ppm): δ 164.77, 164.41, 151.80, 151.23, 151.19, 150.96, 149.32, 148.93, 148.20, 146.72, 146.45, 144.90, 141.40, 141.09, 139.55, 139.49, 138.72, 138.57, 130.42, 128.83, 127.15, 126.88, 126.02, 125.92, 125.27, 124.65, 124.26, 123.50, 123.37, 122.60, 122.54, 122.10, 121.56, 120.35, 120.25, 119.09, 117.46, 115.91, 115.86, 115.03, 113.90, 34.90, 34.89, 34.86, 34.77, 32.40, 31.55, 31.00.

HRMS (positive ESI) calcd for $C_{69}H_{64}AuN_3O_2$: m/z = 1163.4664 $[M]^+$; found 1163.4659 $[M]^+$. Elemental analyses: Found (%): C, 70.95; H, 5.56; N, 3.66. Calcd for $C_{69}H_{64}AuN_3O_2$: C, 71.18; H, 5.54; N, 3.61.

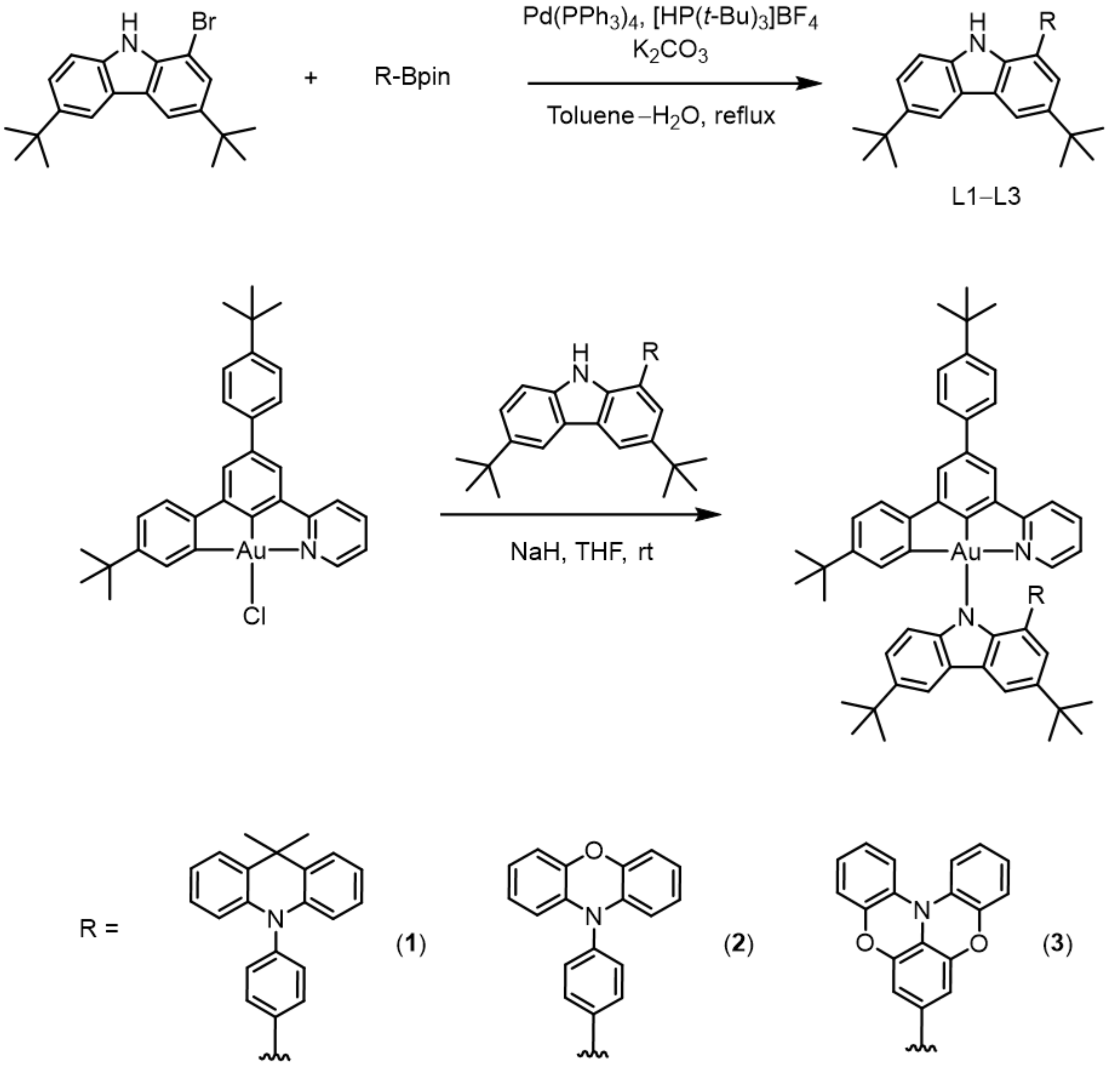

Scheme S1. Synthetic routes for **1**–**3**.

Thermogravimetric Analysis

Thermal analyses were performed on a TA instruments 55 thermogravimetric analyzer (TGA) with a heating rate of 10 °C min^{-1} under nitrogen atmosphere, in which the decomposition temperature (T_d) is defined as the temperature at which the material showed a 5 % weight loss.

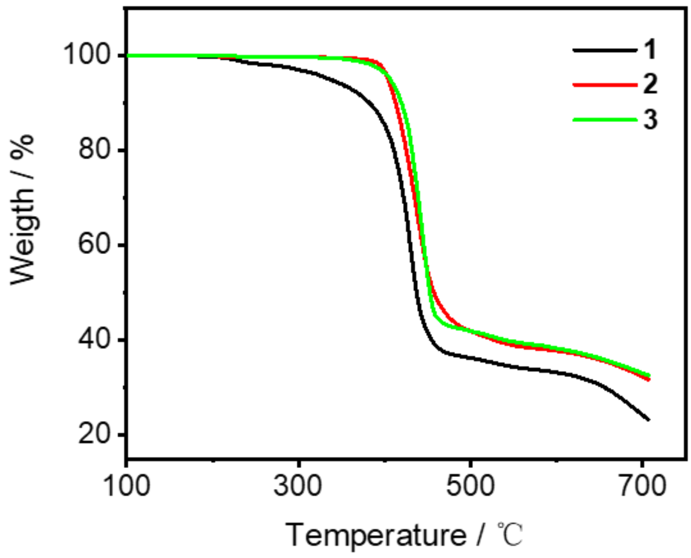

Figure S1. TGA curves of **1–3**.

Table S1. Thermal properties of **1–3**

Complex	T_d / °C[a]
**1**	336
**2**	404
**3**	406

[a] T_d was determined at 5 % weight loss.

X-Ray Crystal Structures

Needle-shaped single crystals of **1** and **2** and parallelogram-shaped single crystals of **3** those are suitable for structure determination by X-ray crystallography have been obtained by laying methanol on a concentrated acetone solution at room temperature. Single-crystal X-ray data were collected using synchrotron radiation λ = 1.54178 Å on an Oxford Diffraction Gemini S Ultra X-ray single-crystal diffractometer for **1** and **2** and on BRUKER APEX II Diffractometer with APEX II CCD Detector for **3**, respectively. The diffraction data reduction and integration were performed by the HKL3000 software. The structures were solved by direct methods and refined employing full-matrix least-squares on F^2 by using SHELXTL (Bruker) software package and expanded using Fourier techniques.[3] All non-H atoms of the compounds were refined by SHELXL with anisotropic thermal parameters. The hydrogen atoms were included in idealized positions and refined with fixed geometry with respect to their carrier atoms. The X-ray crystallographic data for **1–3** have been deposited at the Cambridge Crystallographic Data Centre (CCDC), under the deposition number CCDC 2375750, 2375751 and 2375752 respectively. The data can be obtained free of charge from the Cambridge Crystallographic Data Center via www.ccdc.cam.ac.uk/data_request/cif.

Table S2. Crystal structure determination data of **1**

Empirical formula	$C_{72}H_{72}AuN_3$
Formula weight	1176.29
Temperature, K	193
Wavelength, Å	1.54178
Crystal system	triclinic
Space group	$P\bar{1}$
a, Å	12.7693(5)
b, Å	15.7768(6)
c, Å	16.9743(6)
α, deg	95.273(1)
β, deg	108.880(1)
γ, deg	111.537(1)
Volume, Å^3	2922.22(19)
Z	2
Density (Calcd), g cm^{-3}	1.337
F_{000}	1208.0
θ_{max} for data collection, deg	75.318
Index ranges	$-14 \leq h \leq 15; -19 \leq k \leq 19; -21 \leq l \leq 21$
Reflections collected (unique)	11896
Goodness-of-fit on F^2[a]	1.053
Final R indices [$I > 2\sigma(I)$]	R_1[b] = 0.0343, wR_2[c]= 0.0849

^{a} Goodness-of-fit = $[\Sigma w(|F_{obs}{}^2|-|F_{calc}{}^2|)^2/(N_{obs}-N_{param})]^{1/2}$.

^{b} $R_1 = \Sigma||F_{obs}|-|F_{calc}||/\Sigma|F_{obs}|$.

^{c} $wR_2 = [\Sigma w(|F_{obs}{}^2|-|F_{calc}{}^2|)^2/\Sigma w|F_{obs}{}^2|^2]^{1/2}$.

Table S3. Crystal structure determination data of **2**

Empirical formula	$C_{75}H_{80}AuN_3O$
Formula weight	1236.38
Temperature, K	223
Wavelength, Å	1.54178
Crystal system	triclinic
Space group	$P\bar{1}$
a, Å	12.4468(3)
b, Å	16.7592(4)
c, Å	16.8900(4)
α, deg	100.560(10)
β, deg	105.138(10)
γ, deg	92.523(10)
Volume, $Å^3$	3327.66(14)
Z	2
Density (Calcd), g cm^{-3}	1.234
F_{000}	1276.0
θ_{max} for data collection, deg	133.166
Index ranges	$-14 \leq h \leq 14$; $-19 \leq k \leq 19$; $-20 \leq l \leq 20$
Reflections collected (unique)	11709
Goodness-of-fit on F^2[a]	1.087
Final R indices [$I > 2\sigma(I)$]	R_1[b] = 0.0561, wR_2[c]= 0.1321

^{a} Goodness-of-fit = $[\Sigma w(|F_{obs}{}^2|-|F_{calc}{}^2|)^2/(N_{obs}-N_{param})]^{1/2}$.

^{b} $R_1 = \Sigma||F_{obs}|-|F_{calc}||/\Sigma|F_{obs}|$.

^{c} $wR_2 = [\Sigma w(|F_{obs}{}^2|-|F_{calc}{}^2|)^2/\Sigma w|F_{obs}{}^2|^2]^{1/2}$.

Table S4. Crystal structure determination data of **3**

Empirical formula	$C_{69}H_{64}AuN_3O_2$
Formula weight	1164.19
Temperature, K	100.00
Wavelength, Å	1.54178
Crystal system	orthorhombic
Space group	$P2_12_12_1$
a, Å	11.7846(10)
b, Å	25.277(2)
c, Å	40.015(4)
α, deg	90
β, deg	90
γ, deg	90
Volume, $Å^3$	11919.9(18)
Z	8
Density (Calcd), g cm^{-3}	1.297
F_{000}	4752.0
Crystal size, mm	0.065 × 0.06 × 0.053
θ range for data collection, deg	4.134 to 129.754
Index ranges	$-10 \leq h \leq 13$; $-21 \leq k \leq 29$; $-46 \leq l \leq 46$
Reflections collected (unique)	41952
Goodness-of-fit on F^2[a]	1.071
Final R indices [$I > 2\sigma(I)$]	R_1[b] = 0.0562, wR_2[c] = 0.1257

[a] Goodness-of-fit = $[\Sigma w(|F_{obs}^2|-|F_{calc}^2|)^2/(N_{obs}-N_{param})]^{1/2}$.

[b] $R_1 = \Sigma||F_{obs}|-|F_{calc}||/\Sigma|F_{obs}|$.

[c] $wR_2 = [\Sigma w(|F_{obs}^2|-|F_{calc}^2|)^2/\Sigma w|F_{obs}^2|^2]^{1/2}$.

Table S5. Selected bond distances, bond angles and torsion angles of **1**–**3**

**1**			
Bond distances / Å			
Au1−C1	2.035	Au1−C7	1.974
Au1−N1	2.150	Au1−N2	2.104
Bond angles / °			
C1−Au1−C7	80.66	C7−Au1−N1	79.60
C1−Au1−N2	100.78	N1−Au1−N2	98.72
Torsion angles / °			
C19−C18−C10−C11	45.58	C1−Au1−N2−C24	81.82
C37−C36−C35−C34	38.03	C42−N3−C39−C38	91.05

**2**			
Bond distances / Å			
Au1−N1	2.162	Au1−C11	1.978
Au1−C17	2.026	Au1−N2	2.106
Bond angles / °			
C11−Au1−C17	80.43	C11−Au1−N1	79.29
N1−Au1−N2	100.08	C17−Au1−N2	100.10
Torsion angles / °			
C19−C18−C8−C9	134.05	N1−Au1−N2−C32	98.86
C45−C44−C40−C39	43.39	C50−N3−C47−C46	102.04

3

Bond distances / Å			
Au1–C28	2.010	Au1–C12	1.983
Au1–N1	2.150	Au1–N33	2.075

Bond angles / °			
C12–Au1–C28	80.89	C12–Au1–N1	79.66
C28–Au1–N33	99.92	N1–Au1–N33	99.55

Torsion angles / °			
C10–C9–C13–C18	19.40	C28–Au1–N33–C34	71.96
C59–C54–C44–C45	70.57	C66–N67–C68–C69	20.05

Photophysical Properties

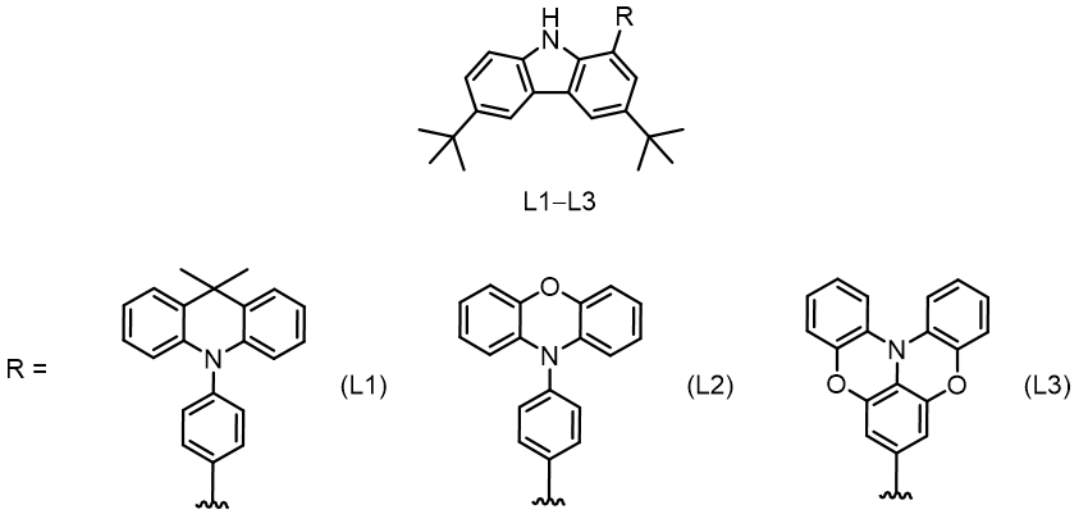

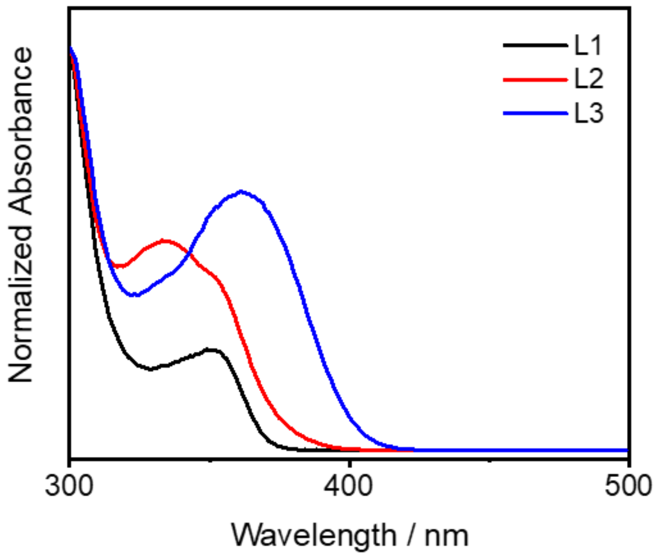

Figure S2. Normalized absorption spectra of ligands L1–L3 in toluene solution at 298 K.

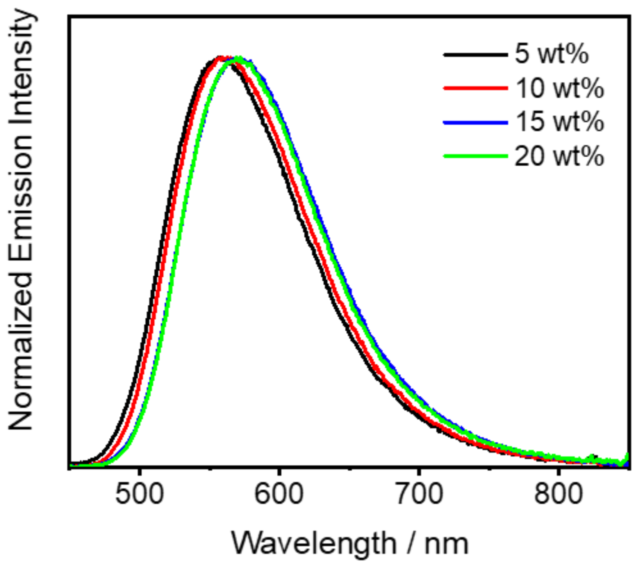

Figure S3. Normalized emission spectra of thin films of **1** doped into mCP at different concentrations (wt%) at 298 K.

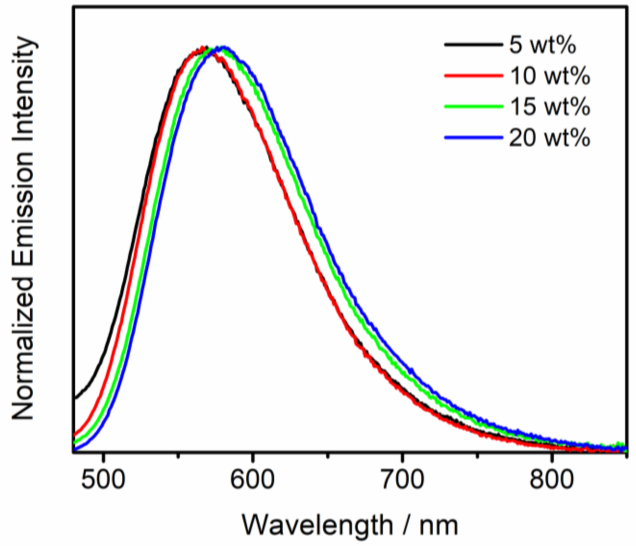

Figure S4. Normalized emission spectra of thin films of **2** doped into mCP at different concentrations (wt%) at 298 K.

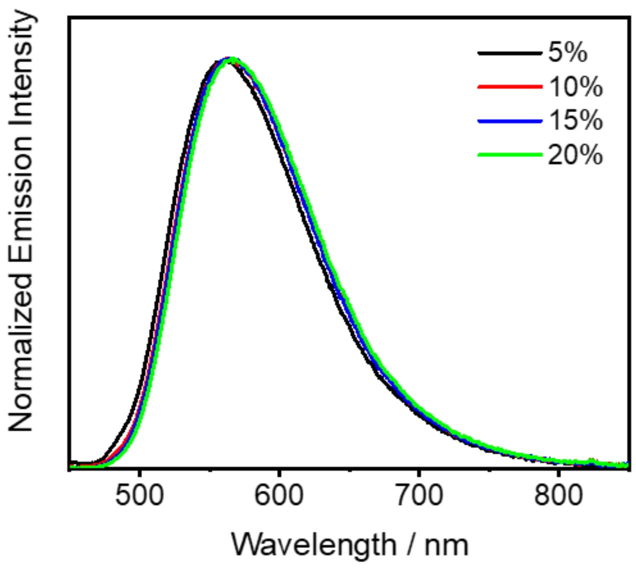

Figure S5. Normalized emission spectra of thin films of **3** doped into mCP at different concentrations (wt%) at 298 K.

Table S6. Luminescence lifetimes of mCP thin films of **1**–**3** doped at various concentrations

Complex	Dopant concentration / wt%	τ_{T1} / μs (%)[a]	$\tau_{T1'}$ / μs (%)[a]
**1**	5	5.5 (29.8)	2.6 (70.2)
	10	5.6 (44.6)	2.7 (55.4)
	15	4.0 (56.4)	2.0 (43.6)
	20	3.9 (58.0)	2.0 (42.0)
**2**	5	5.4 (40.8)	2.3 (59.2)
	10	4.7 (47.7)	2.2 (52.3)
	15	4.1 (46.9)	1.9 (53.1)
	20	3.6 (55.2)	1.7 (44.8)
**3**	5	10.7 (27.4)	4.3 (72.6)
	10	8.0 (33.6)	3.6 (66.4)
	15	7.1 (39.9)	3.3 (60.1)
	20	6.2 (50.4)	3.0 (49.6)

[a] Percentage contribution in parentheses, calculated from the amplitudes and decay constants of τ_{T1} and $\tau_{T1'}$ respectively.

Variable-Temperature Emission Studies

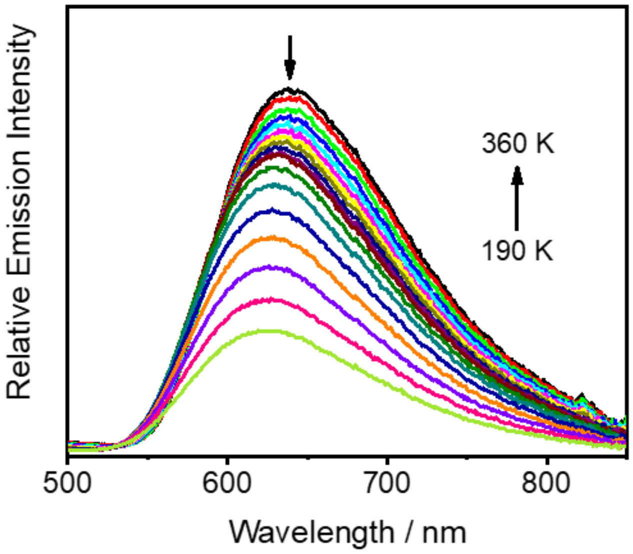

Figure S6. Emission spectra of **1** in degassed toluene solution at temperatures between 190 and 360 K.

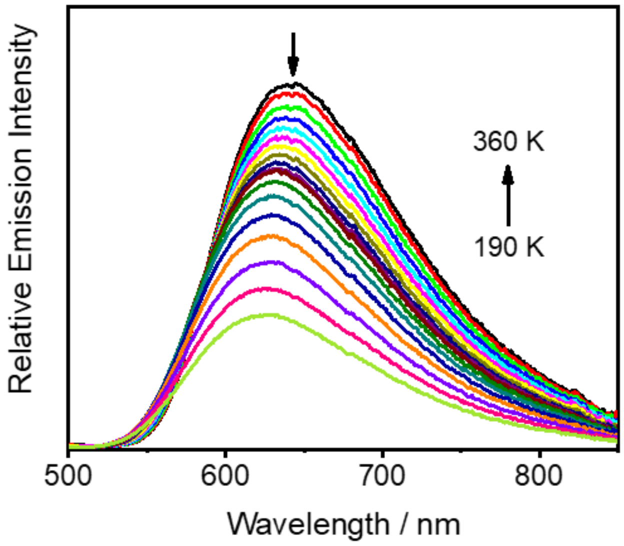

Figure S7. Emission spectra of **2** in degassed toluene solution at temperatures between 190 and 360 K.

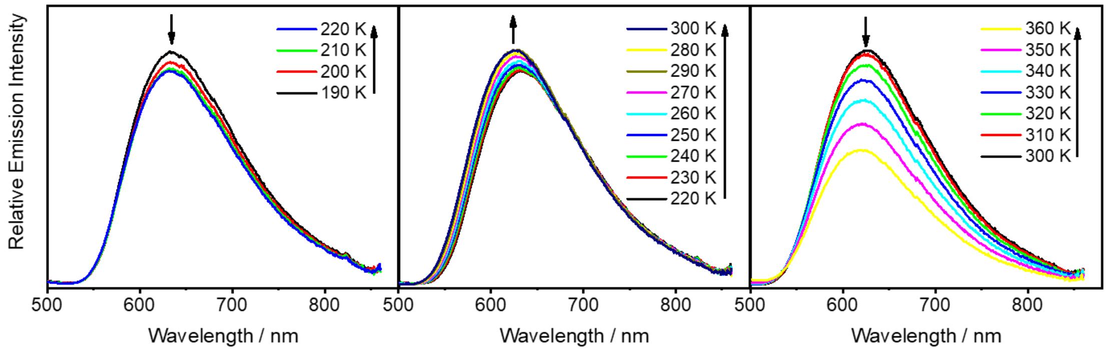

Figure S8. Emission spectra of **3** in degassed toluene solution at temperatures between 190 and 360 K.

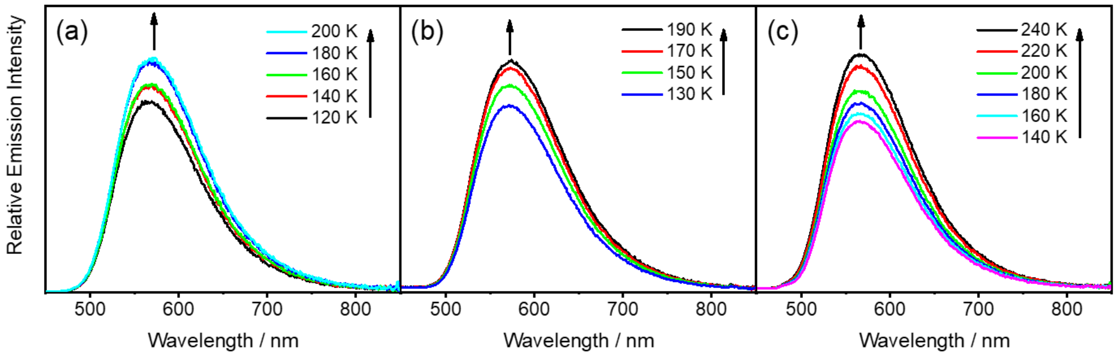

Figure S9. Emission spectra of 20 wt% doped mCP thin films at temperatures (a) between 120 and 200 K for **1**, (b) between 130 and 190 K for **2** and (c) between 140 and 240 K for **3**.e

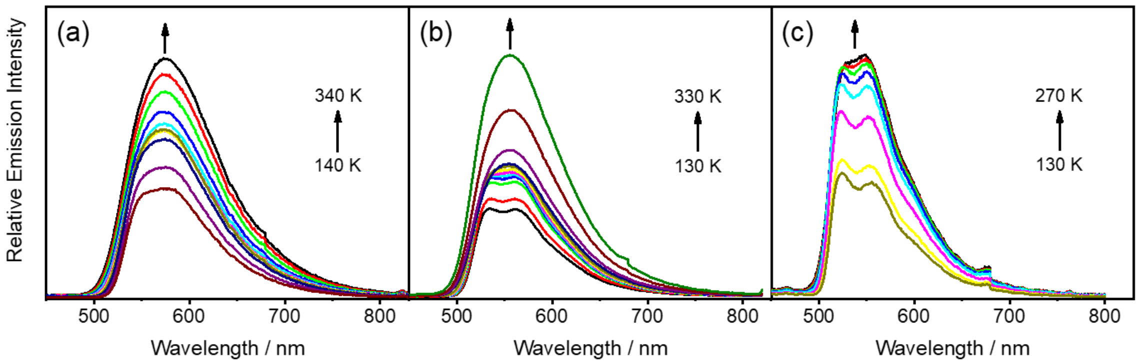

Figure S10. Emission spectra in solid state at temperatures (a) between 140 and 340 K for **1**, (b) between 130 and 330 K for **2** and (c) between 130 and 270 K for **3**.

Determination of $\Delta E_{T1'-T1}$ According to A Modified Three-State Boltzmann Equation

The plot of averaged lifetime (τ_{ave})[4] of 20 wt% **1–3** doped mCP thin films as a function of temperature can be well-fitted using a modified three-state Boltzmann equation (see below),[5,6] where k_B is the Boltzmann constant and τ_{T1} and $\tau_{T1'}$ are the intrinsic decay times of the emitting lowest triplet excited state and the energetically close-lying higher triplet excited state respectively.

$$\tau = \frac{1 + \exp\frac{-\Delta E(T_1' - T_1)}{k_B T}}{\frac{1}{\tau_{T1}} + \frac{1}{\tau_{T1'}}\exp\frac{-\Delta E(T_1' - T_1)}{k_B T}}$$

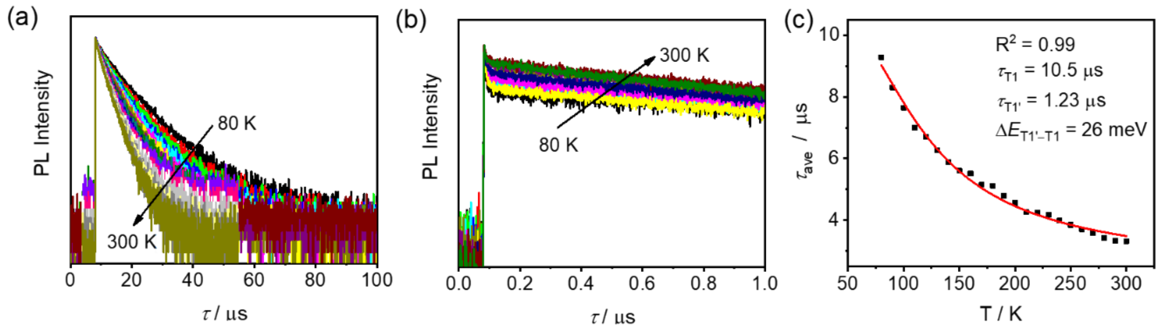

Figure S11. (a, b) PL decay profiles of 20 wt% doped mCP thin film for **1** at 80–300 K and (c) the corresponding plot of average lifetime (τ_{ave}) measured in the time range of 100 μs against temperature (excitation wavelength at 340 nm).

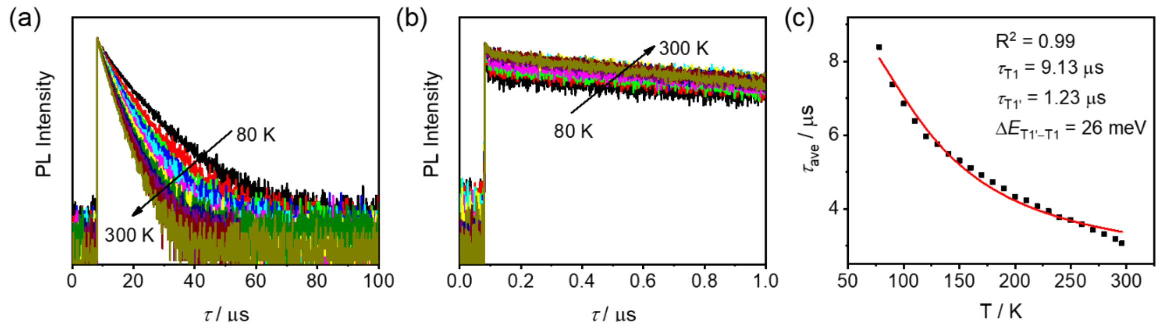

Figure S12. (a, b) PL decay profiles of 20 wt% doped mCP thin film for **2** at 80–300 K and (c) the corresponding plot of average lifetime (τ_{ave}) measured in the time range of 100 μs against temperature (excitation wavelength at 340 nm).

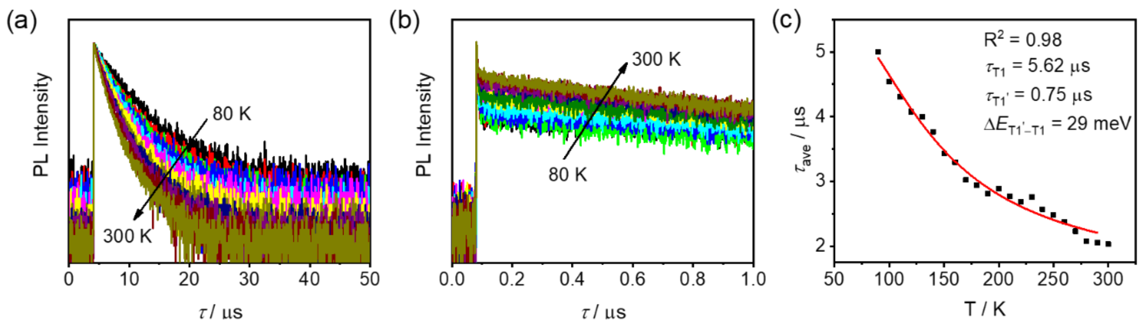

Figure S13. (a, b) PL decay profiles of **1** in the solid state at 80–300 K and (c) the corresponding plot of average lifetime (τ_{ave}) measured in the time range of 100 µs against temperature (excitation wavelength at 340 nm).

Table S7a. Luminescence lifetimes of 20 wt% **1** doped mCP thin films measured in time range of 1 μs at different temperatures

Temperature / K	τ_1 / ns	τ_2 / μs	A_1[a]	A_2[b]
80	6.0	2.9	555.51	613.06
100	5.5	2.7	653.73	744.65
120	7.4	2.5	509.78	833.37
140	5.6	2.4	632.39	896.12
160	6.8	2.3	655/89	846.68
180	6.0	2.1	664.23	882.59
200	6.5	2.2	761.47	815.92
220	10.1	2.0	340.68	1089.10
240	9.5	2.1	421.37	1048.23
260	8.8	1.9	391.59	1185.18
280	8.4	1.9	350.32	1271.31
300	6.6	1.8	399.00	1258.64

[a] Pre-exponential factor for τ_1

[b] Pre-exponential factor for τ_2

Table S7b. Luminescence lifetimes of 20 wt% **1** doped mCP thin films measured in time range of 50–100 μs at different temperatures.

Temperature / K	τ_{ave} / μs	τ_1 / μs	τ_2 / μs
80	9.28	5.66	13.15
100	7.65	4.92	11.09
120	6.70	4.36	10.01
140	5.88	3.49	7.82
160	5.52	3.70	8.28
180	5.12	3.32	7.58
200	4.55	2.58	5.69
220	4.25	2.59	5.36
240	3.99	2.29	4.89
260	3.71	2.30	4.59
280	3.63	2.39	4.96
300	3.32	2.21	4.41

Table S8. Luminescence lifetimes of 20 wt% **2** doped mCP thin films measured in time range of 1 μs at different temperatures

Temperature / K	τ_1 / ns	τ_2 / μs	A_1[a]	A_2[b]
80	8.2	2.7	164.21	356.62
100	5.9	2.4	218.32	436.44
120	7.9	2.3	163.65	458.65
140	19.8	2.4	91.55	524.25
160	18.4	2.2	52.94	570.08
180	8.3	2.2	143.98	547.81
200	14.7	2.2	51.36	624.01
220	23.1	2.1	65.81	631.62
240	39.2	2.0	41.49	639.61
260	55.9	2.0	38.58	655.85
280	78.9	2.0	30.58	699.73
300	46.5	1.8	61.38	693.52

[a] Pre-exponential factor for τ_1

[b] Pre-exponential factor for τ_2

Table S9. Luminescence lifetimes of 20 wt% **3** doped mCP thin films measured in time range of 1 μs at different temperatures

Temperature / K	τ_1 / ns	τ_2 / μs	A_1[a]	A_2[b]
80	11.6	3.1	169.79	109.97
100	7.9	2.9	214.18	124.81
120	7.7	2.5	160.76	124.37
140	8.4	2.8	170.28	189.54
160	9.3	2.6	145.72	217.30
180	8.8	2.8	127.74	281.48
200	9.0	2.4	146.02	316.33
220	10.0	2.5	132.15	356.82
240	12.2	2.4	100.40	384.81
260	15.5	2.4	87.83	324.64
280	7.3	2.4	107.82	414.33
300	7.3	2.2	211.74	448.69

[a] Pre-exponential factor for τ_1

[b] Pre-exponential factor for τ_2

Transient Absorption Studies

Complex **2**

The evolution of the TA signals in the ps regime are similar to that of **1**. The signal shows an initial rise with time constants ~400 fs (τ_1) with absorption peaks at 558 and 756 nm (part of the signal is masked by the 800 nm laser peak, but to a smaller extent compared to complex **1**) Then, a decrease of signal in the 600–750 nm region is observed (τ_2, 2.2–17.9 ps), followed by a slower rise of signal in the 550–650 nm region. (τ_3, 19.1–309.4 ps) After that, the signal shows a decay with time-constants beyond our instrument time-window (τ_3, > 20 ns).

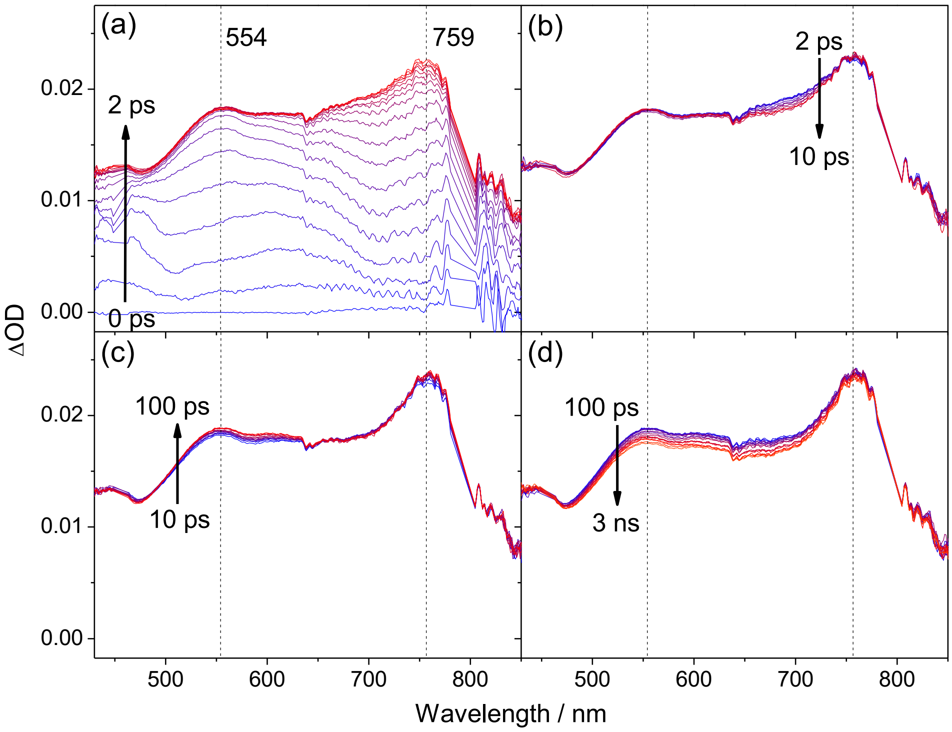

Figure S14. Selected fs-TA spectra of **2** in toluene solution at 293 K recorded at (a) 0–2, (b) 2–10, (c) 10–100 and (d) 100–3000 ps after 400 nm laser excitation.

Complex **3**

The evolution of the TA signals in the ps regime are similar to that of **1**. The evolution of the TA signal in the ps regime consist of four parts, which includes an initial rise with

time constants ~350 fs (τ_1) with absorption maximum at 488, 625 and 681 nm. Then, the signals are redshifted to 503, 634 and 691 nm respectively (τ_2, 2.6–14.4 ps), followed by a slower rise of signal in the 550–650 nm region. (τ_3, 24.7–568.6 ps) After that, the signal shows a decay with time-constants beyond our instrument time-window (τ_4, > 20 ns).

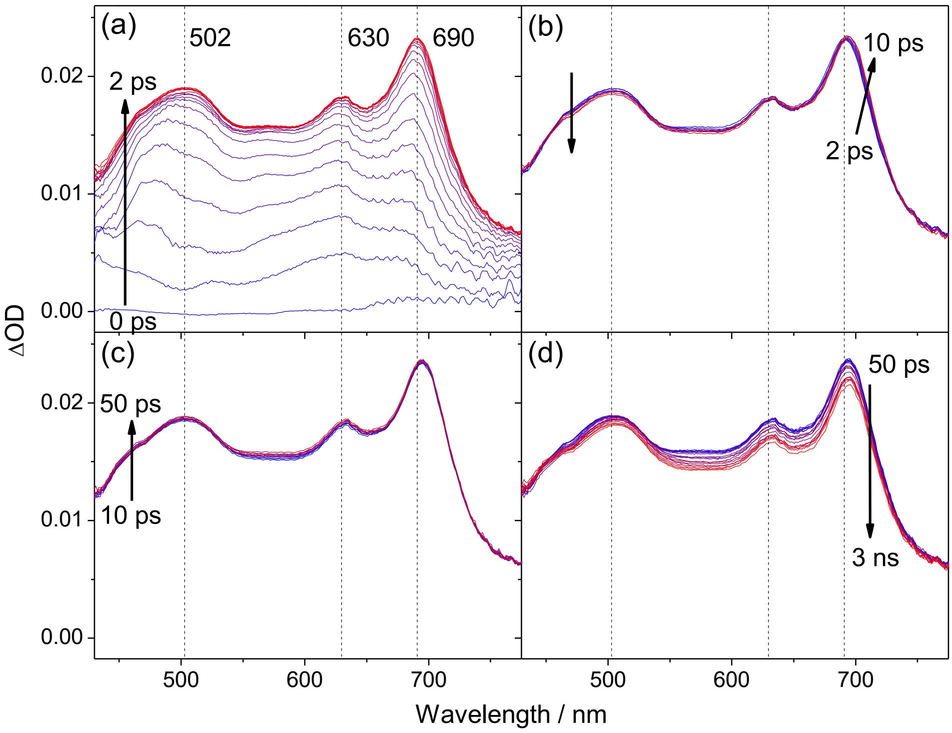

Figure S15. Selected fs-TA spectra of **3** in toluene solution at 293 K recorded at (a) 0–2, (b) 2–10, (c) 10–50 and (d) 50–3000 ps after 400 nm laser excitation.

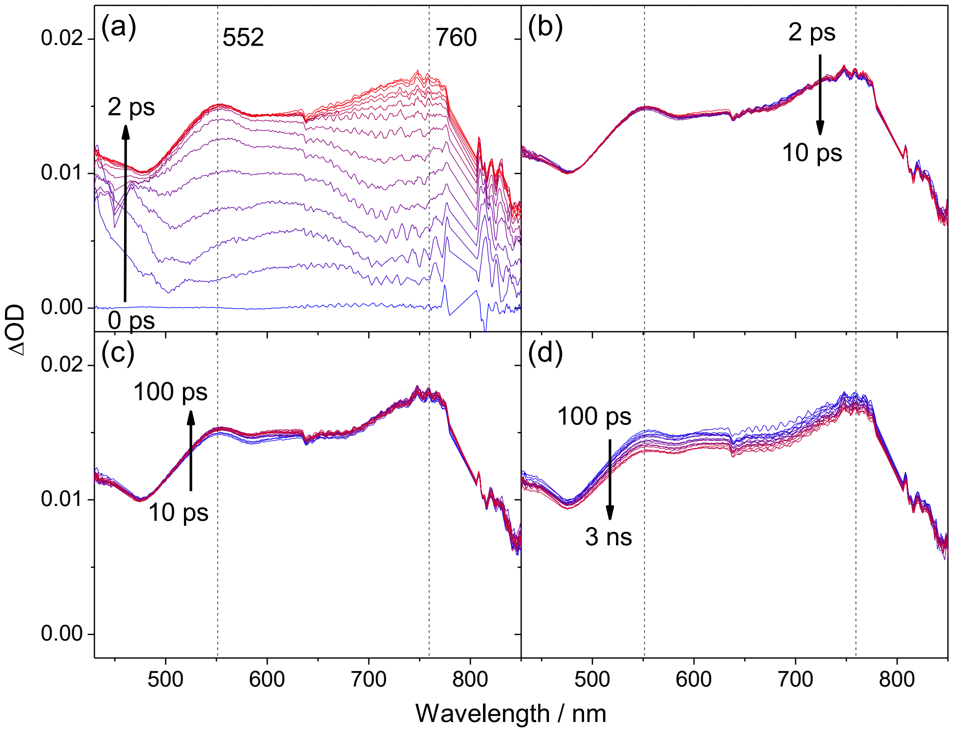

Figure S16. Selected fs-TA spectra of **1** in toluene solution at 354 K recorded at (a) 0–2, (b) 2–10, (c) 10–100 and (d) 100–3000 ps after 400 nm laser excitation.

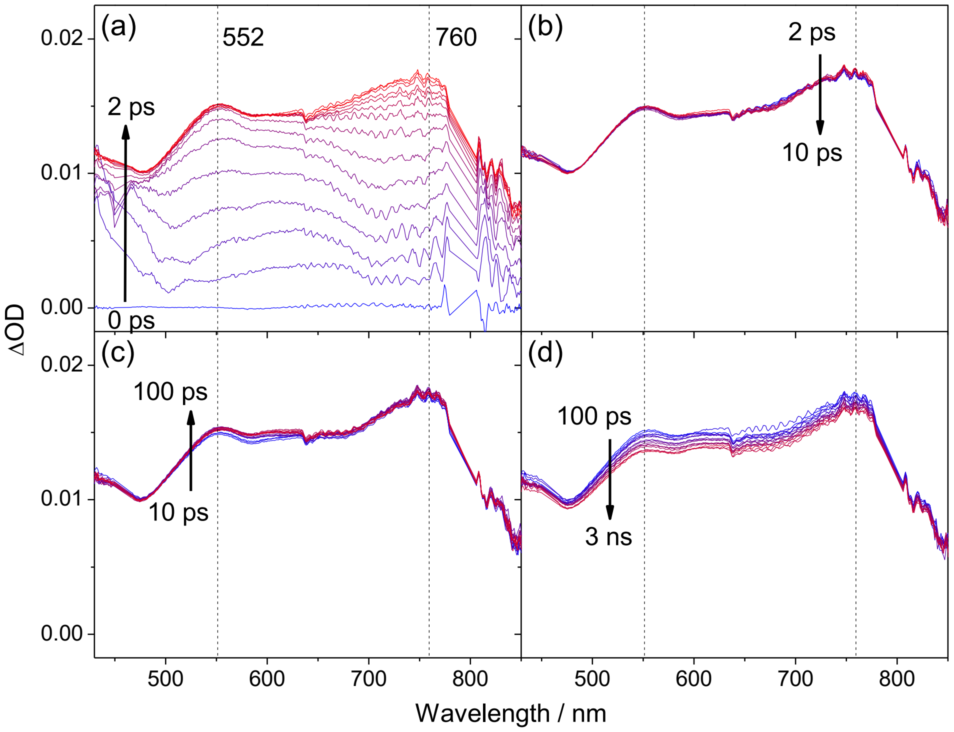

Figure S17. Selected fs-TA spectra of **1** in toluene solution at 332 K recorded at (a) 0–2, (b) 2–10, (c) 10–100 and (d) 100–3000 ps after 400 nm laser excitation.

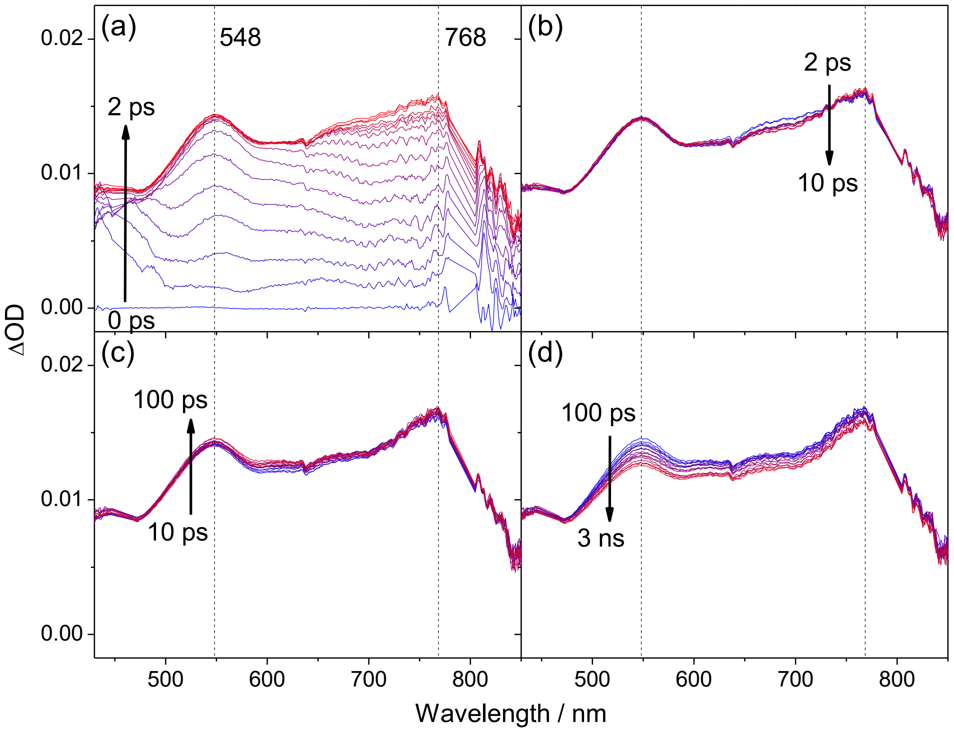

Figure S18. Selected fs-TA spectra of **1** in toluene solution at 311 K recorded at (a) 0–2, (b) 2–10, (c) 10–100 and (d) 100–3000 ps after 400 nm laser excitation.

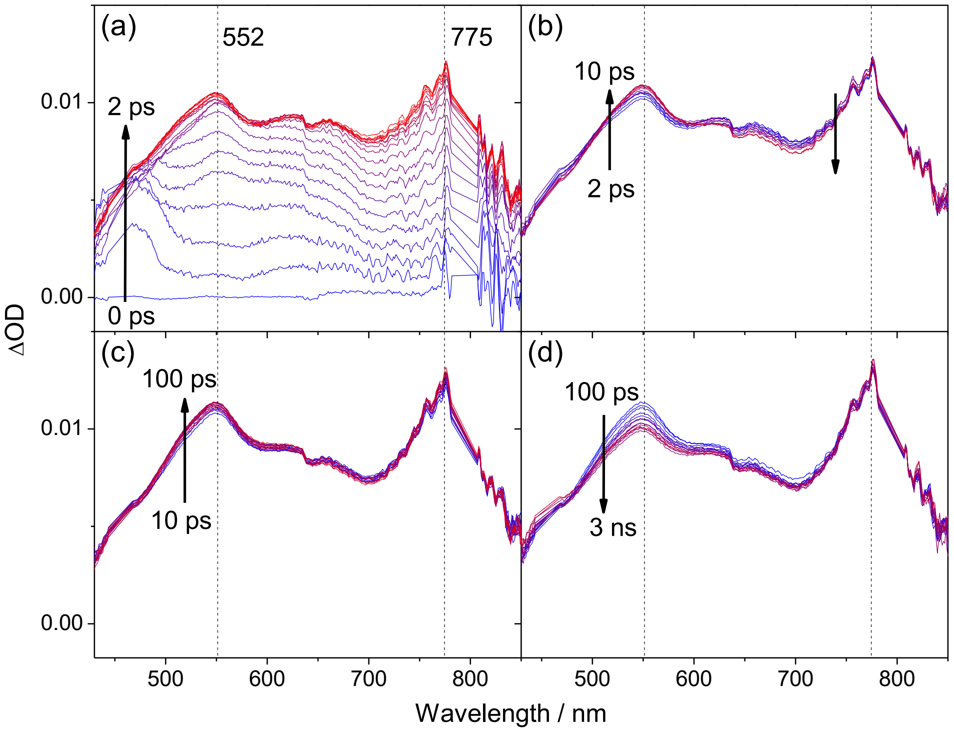

Figure S19. Selected fs-TA spectra of **1** in toluene solution at 258 K recorded at (a) 0–2, (b) 2–10, (c) 10–100 and (d) 100–3000 ps after 400 nm laser excitation.

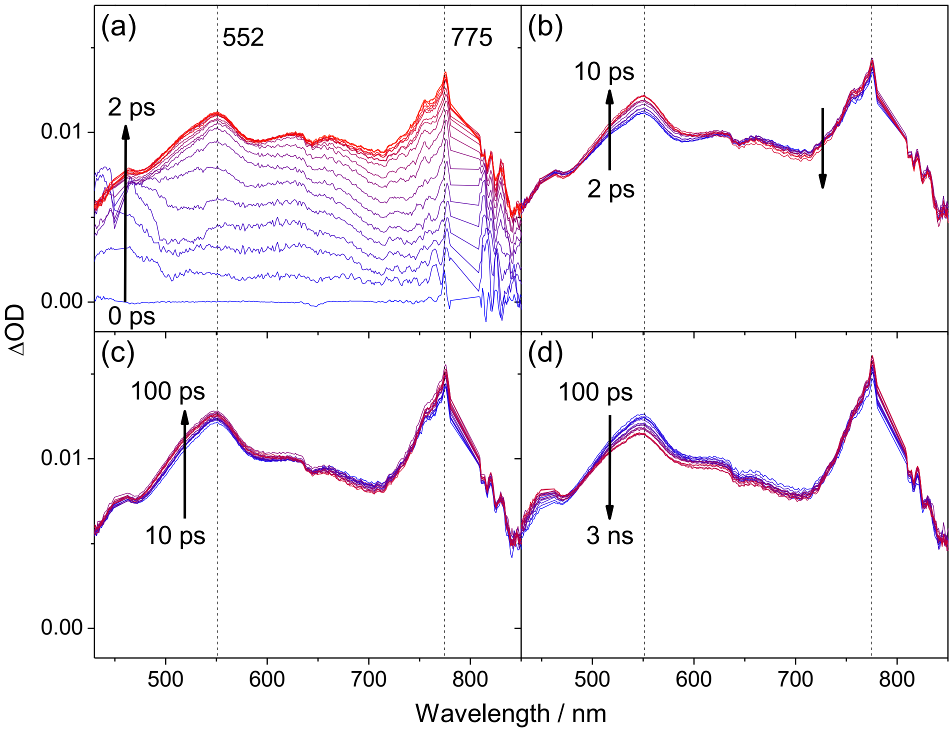

Figure S20. Selected fs-TA spectra of **1** in toluene solution at 238 K recorded at (a) 0–2, (b) 2–10, (c) 10–100 and (d) 100–3000 ps after 400 nm laser excitation.

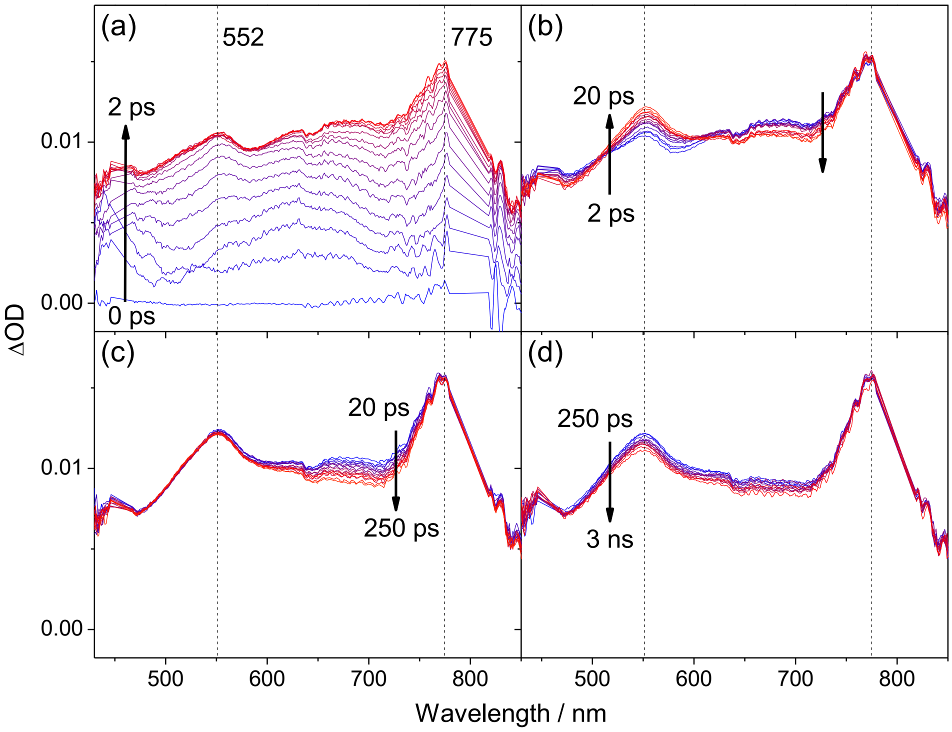

Figure S21. Selected fs-TA spectra of **1** in toluene solution at 213 K recorded at (a) 0–2, (b) 2–20, (c) 20–250 and (d) 250–3000 ps after 400 nm laser excitation.

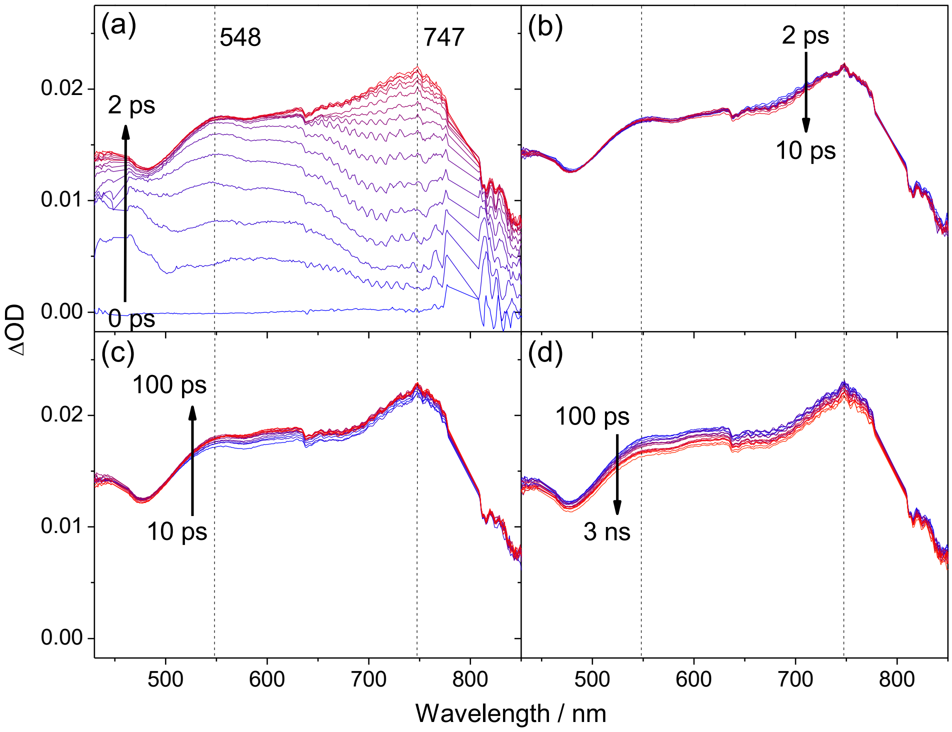

Figure S22. Selected fs-TA spectra of **2** in toluene solution at 354 K recorded at (a) 0–2, (b) 2–10, (c) 10–100 and (d) 100–3000 ps after 400 nm laser excitation.

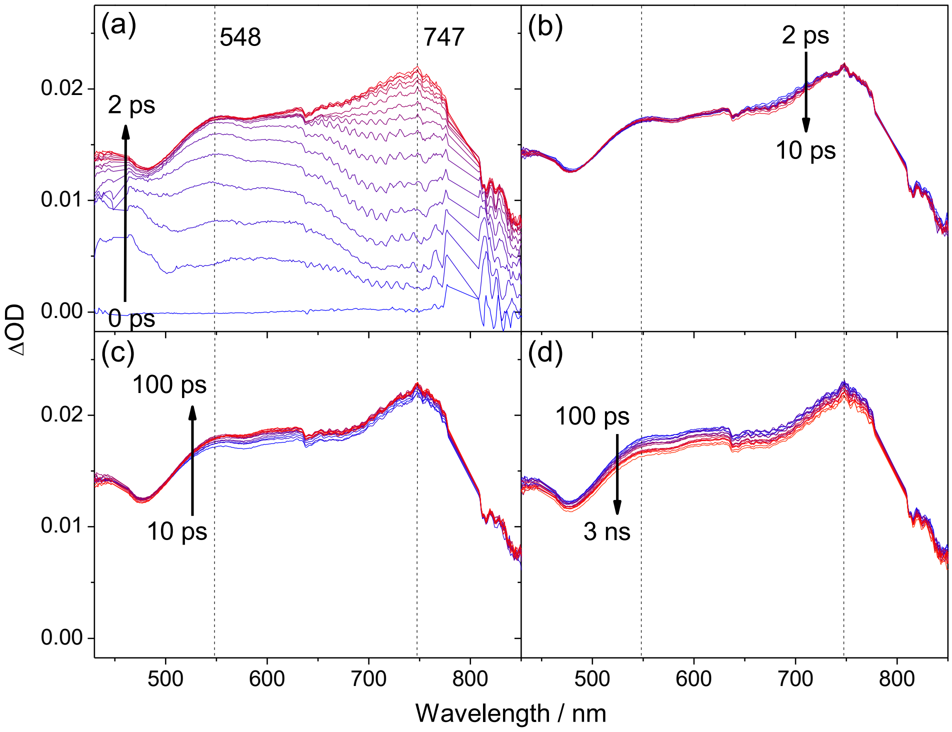

Figure S23. Selected fs-TA spectra of **2** in toluene solution at 332 K recorded at (a) 0–2, (b) 2–10, (c) 10–100 and (d) 100–3000 ps after 400 nm laser excitation.

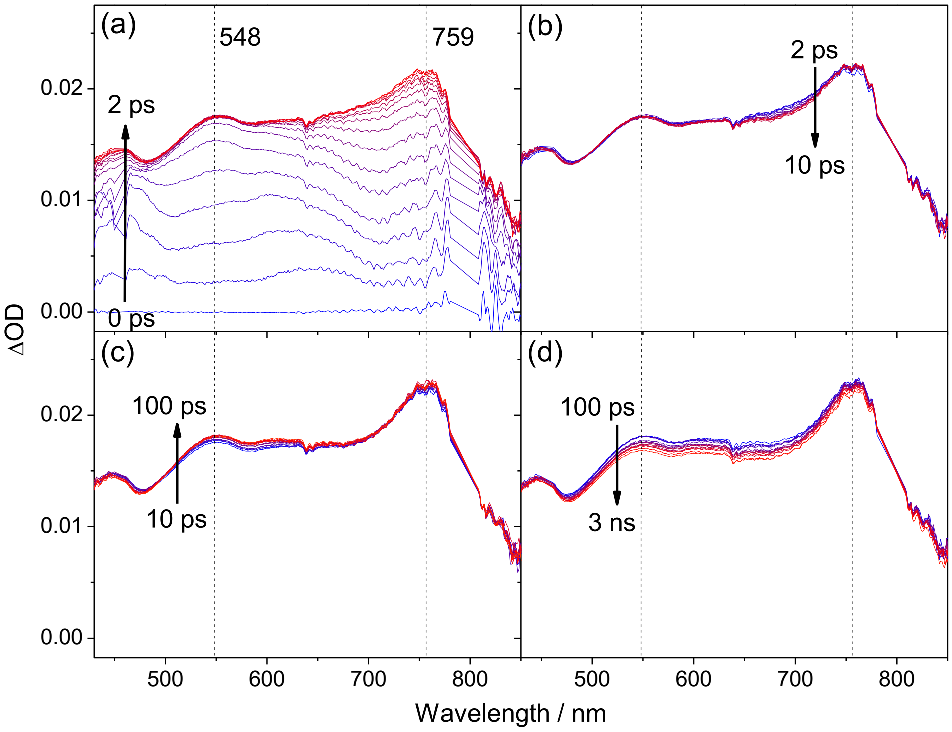

Figure S24. Selected fs-TA spectra of **2** in toluene solution at 311 K recorded at (a) 0–2, (b) 2–10, (c) 10–100 and (d) 100–3000 ps after 400 nm laser excitation.

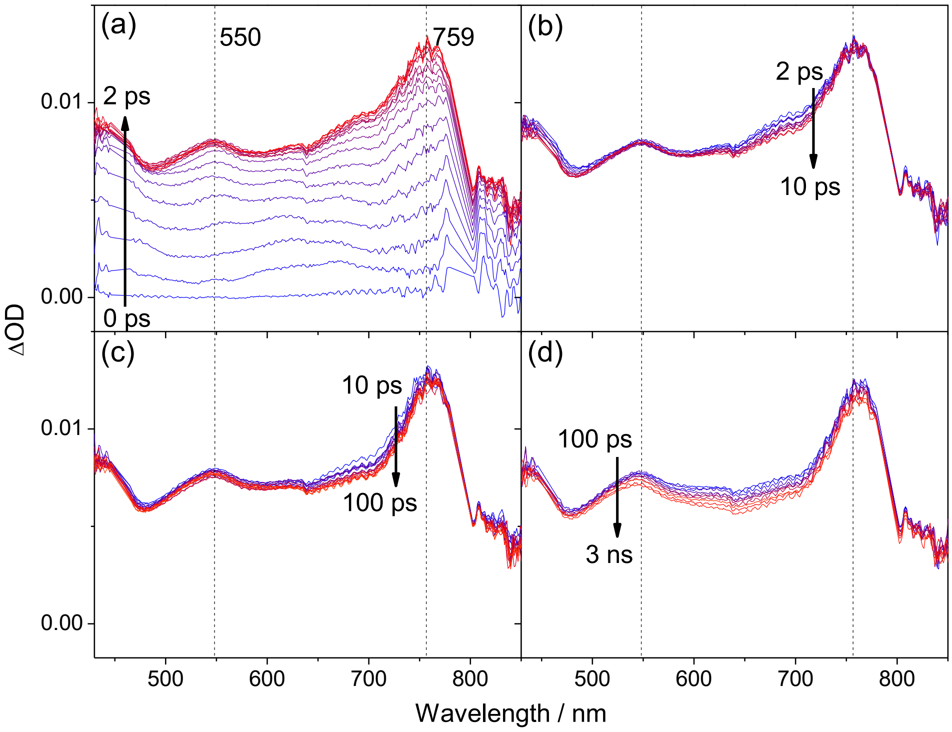

Figure S25. Selected fs-TA spectra of **2** in toluene solution at 258 K recorded at (a) 0–2, (b) 2–10, (c) 10–100 and (d) 100–3000 ps after 400 nm laser excitation.

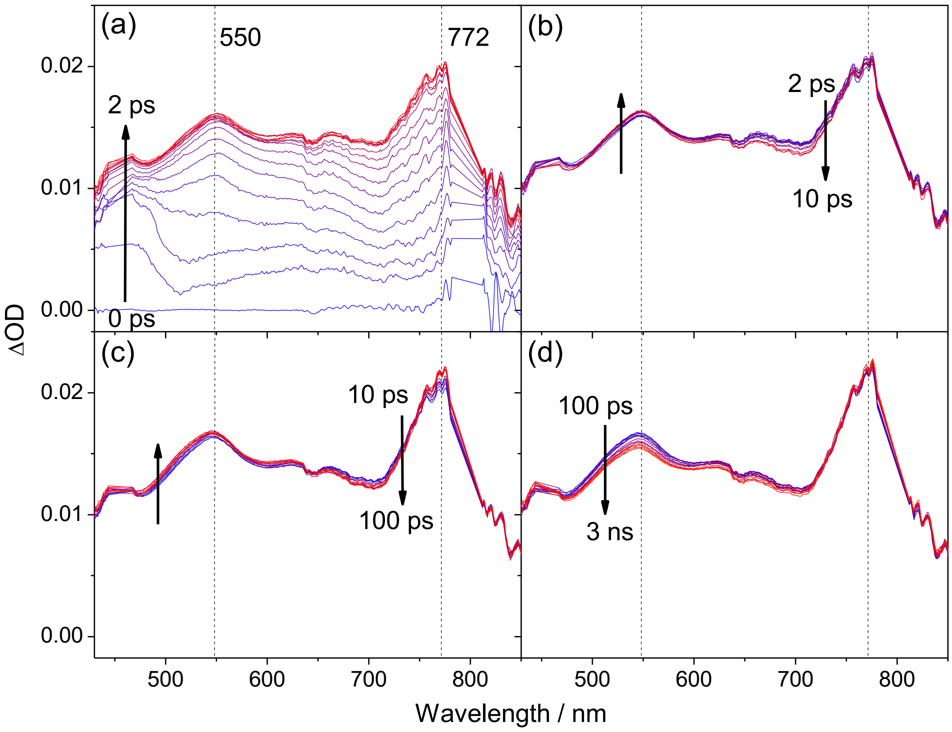

Figure S26. Selected fs-TA spectra of **2** in toluene solution at 238 K recorded at (a) 0–2, (b) 2–10, (c) 10–100 and (d) 100–3000 ps after 400 nm laser excitation.

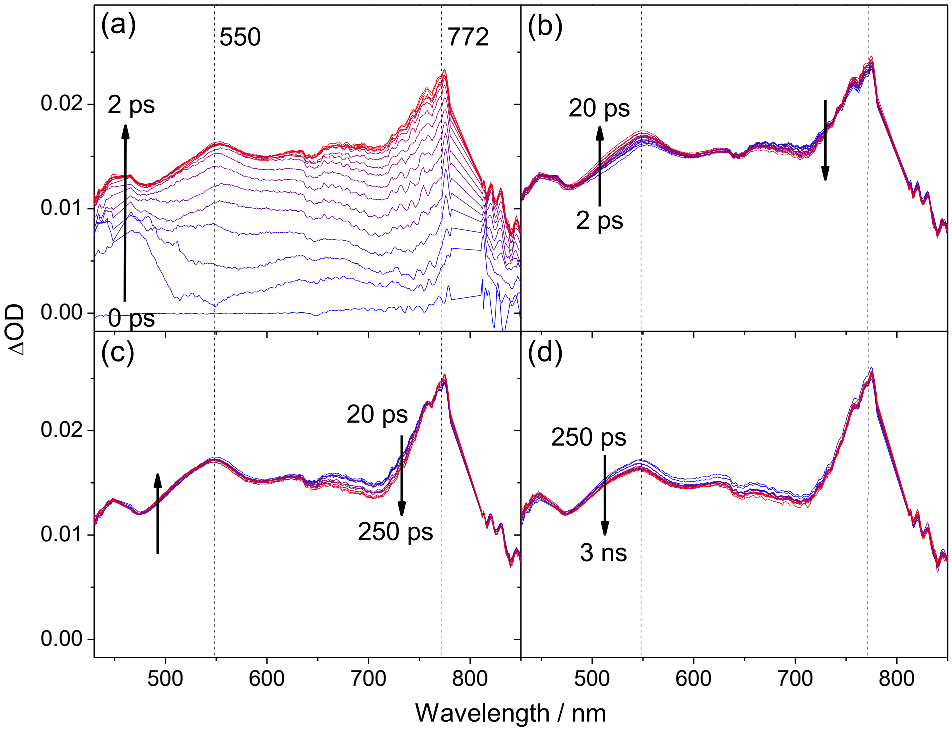

Figure S27. Selected fs-TA spectra of **2** in toluene solution at 213 K recorded at (a) 0–2, (b) 2–20, (c) 20–250 and (d) 250–3000 ps after 400 nm laser excitation.

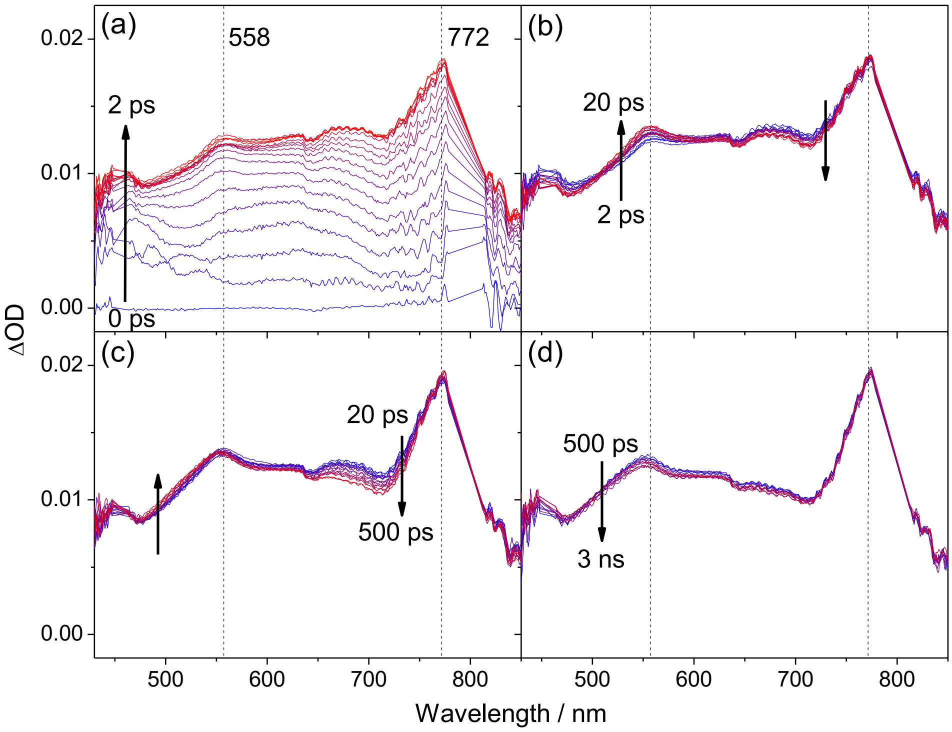

Figure S28. Selected fs-TA spectra of **2** in toluene solution at 188 K recorded at (a) 0–2, (b) 2–20, (c) 20–500 and (d) 500–3000 ps after 400 nm laser excitation.

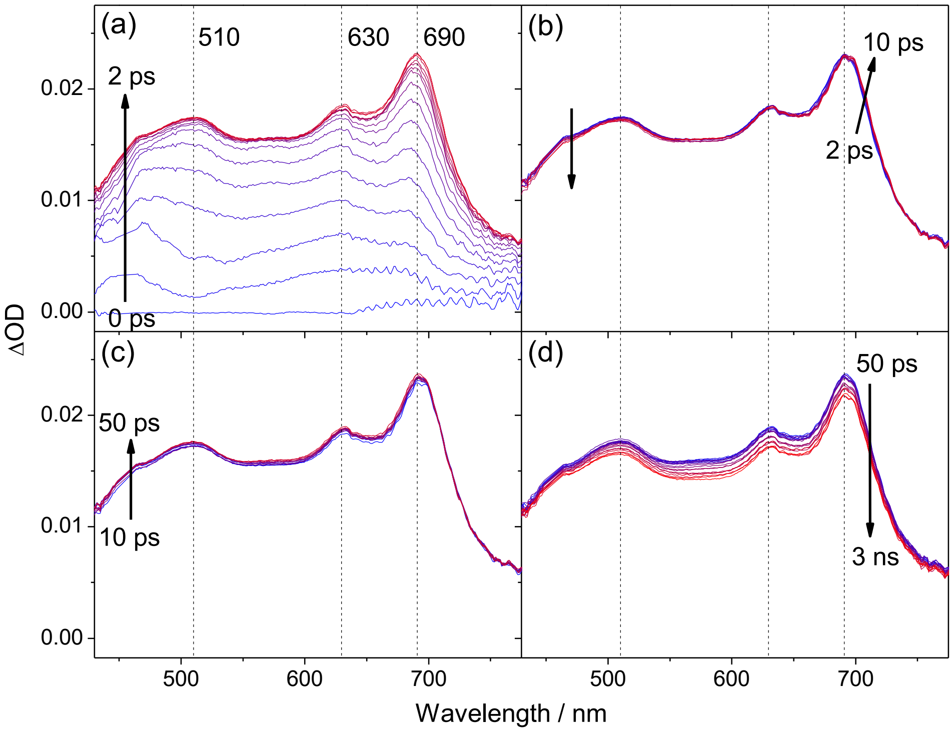

Figure S29. Selected fs-TA spectra of **3** in toluene solution at 354 K recorded at (a) 0–2, (b) 2–10, (c) 10–50 and (d) 50–3000 ps after 400 nm laser excitation.

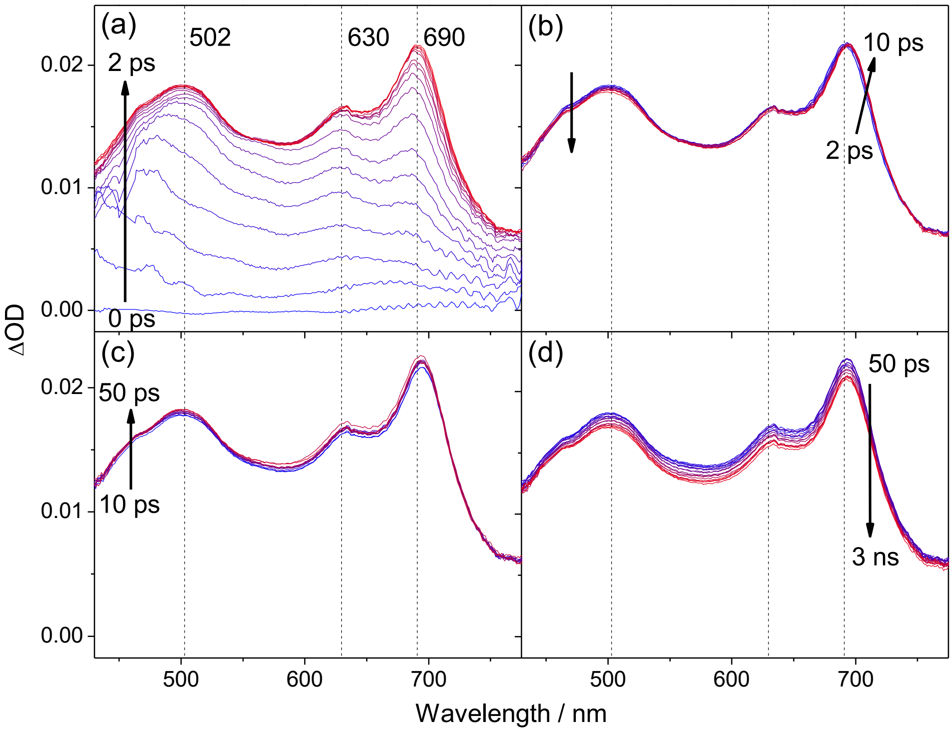

Figure S30. Selected fs-TA spectra of **2** in toluene solution at 322 K recorded at (a) 0–2, (b) 2–10, (c) 10–50 and (d) 50–3000 ps after 400 nm laser excitation.

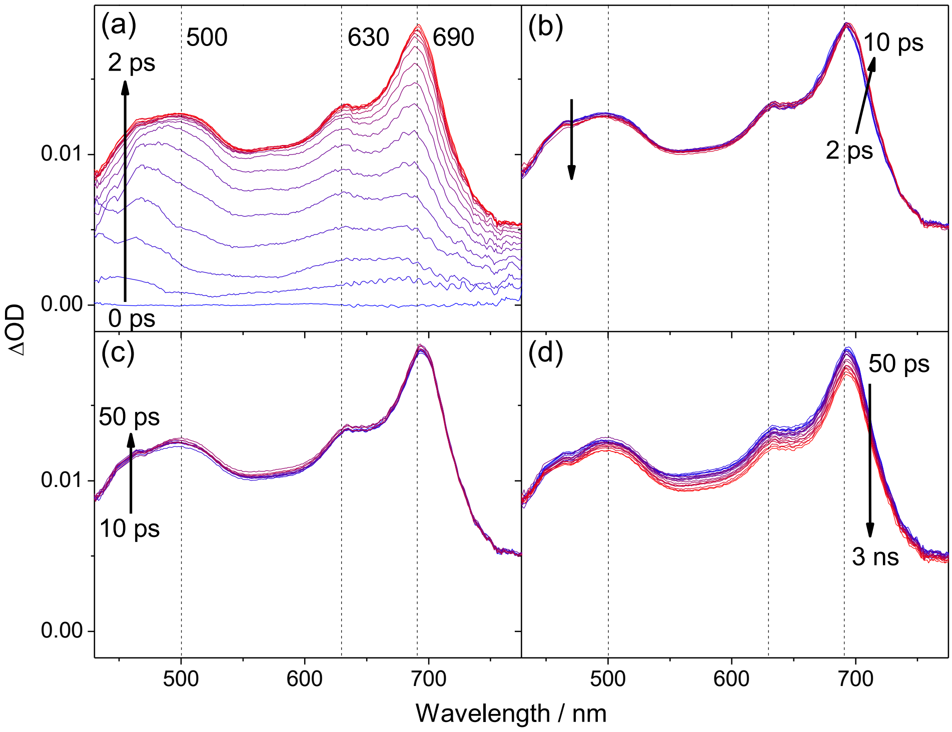

Figure S31. Selected fs-TA spectra of **2** in toluene solution at 311 K recorded at (a) 0–2, (b) 2–10, (c) 10–50 and (d) 50–3000 ps after 400 nm laser excitation.

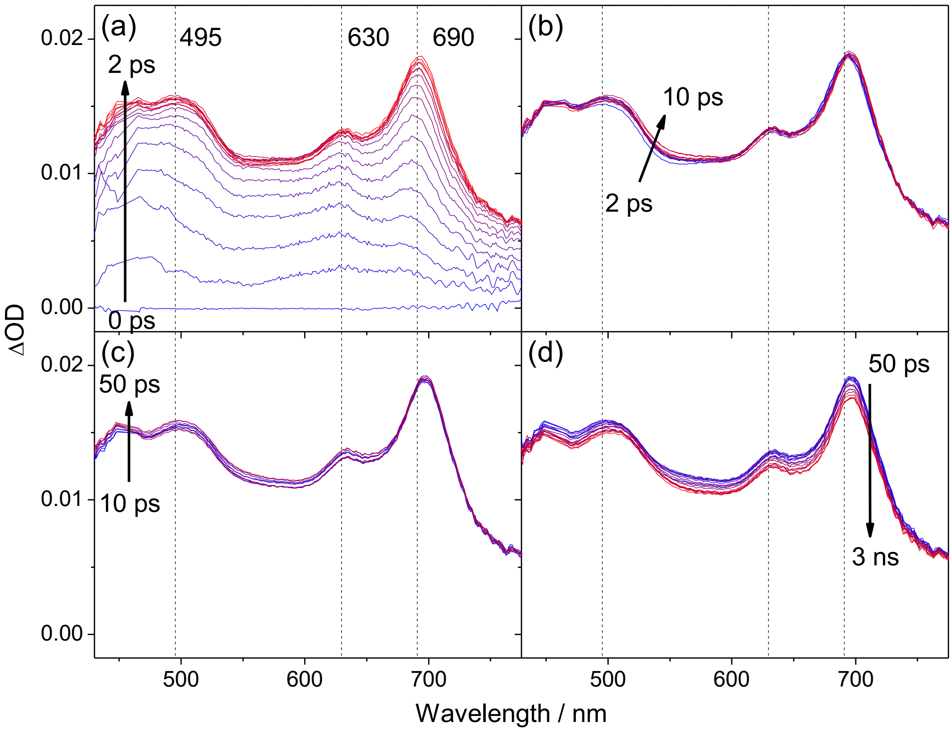

Figure S32. Selected fs-TA spectra of **3** in toluene solution at 273 K recorded at (a) 0–2, (b) 2–10, (c) 10–50 and (d) 50–3000 ps after 400 nm laser excitation.

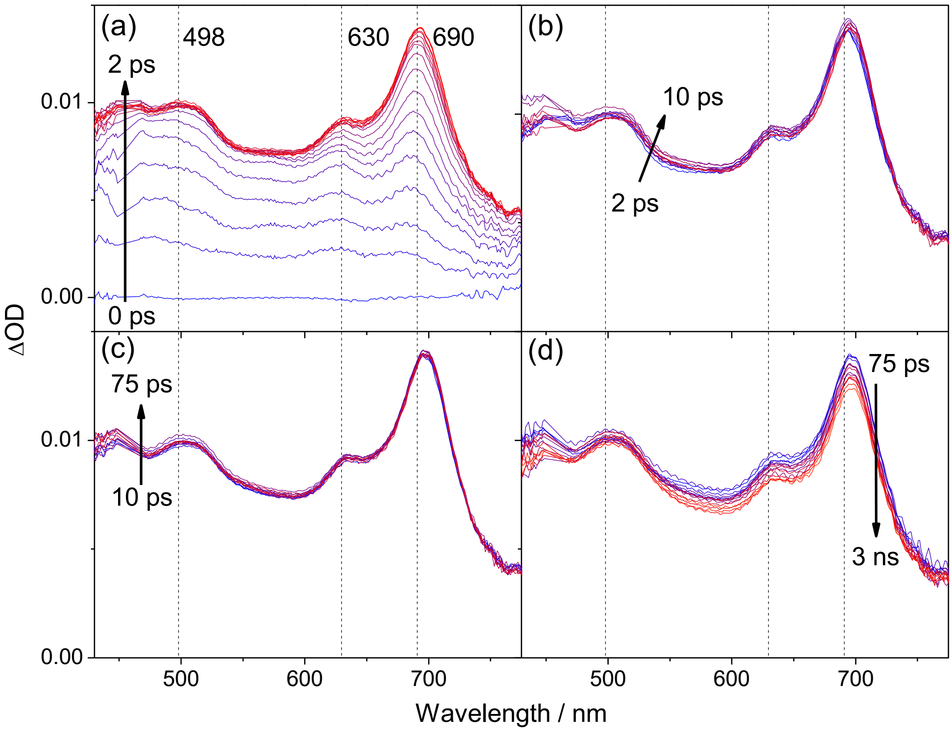

Figure S33. Selected fs-TA spectra of **3** in toluene solution at 258 K recorded at (a) 0–2, (b) 2–10, (c) 10–75 and (d) 75–3000 ps after 400 nm laser excitation.

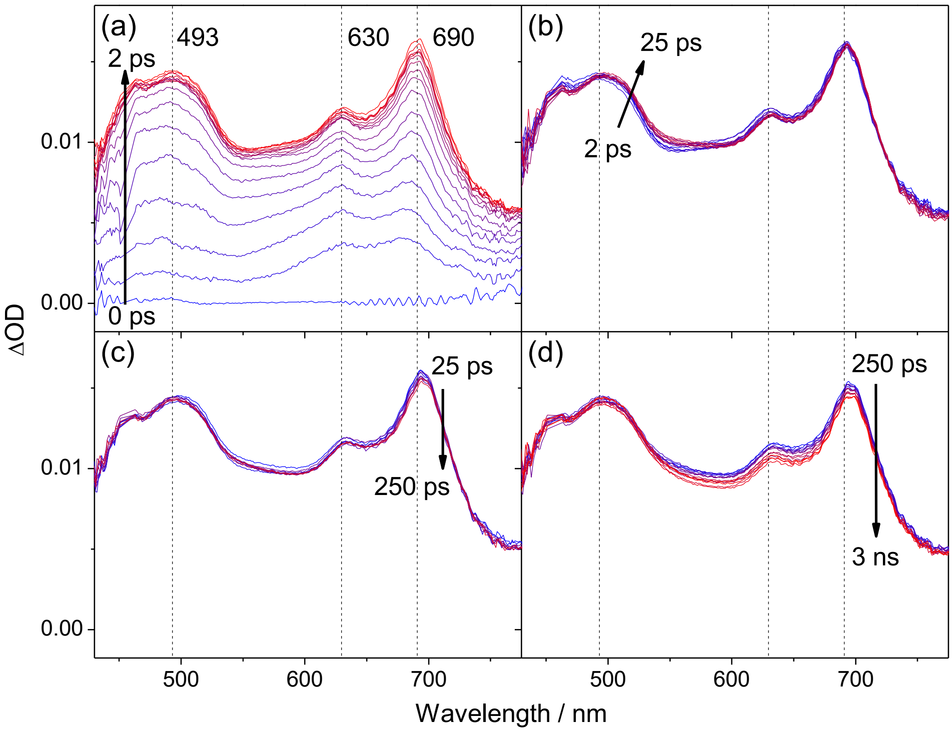

Figure S34. Selected fs-TA spectra of **3** in toluene solution at 213 K recorded at (a) 0–2, (b) 2–25, (c) 25–250 and (d) 250–3000 ps after 400 nm laser excitation.

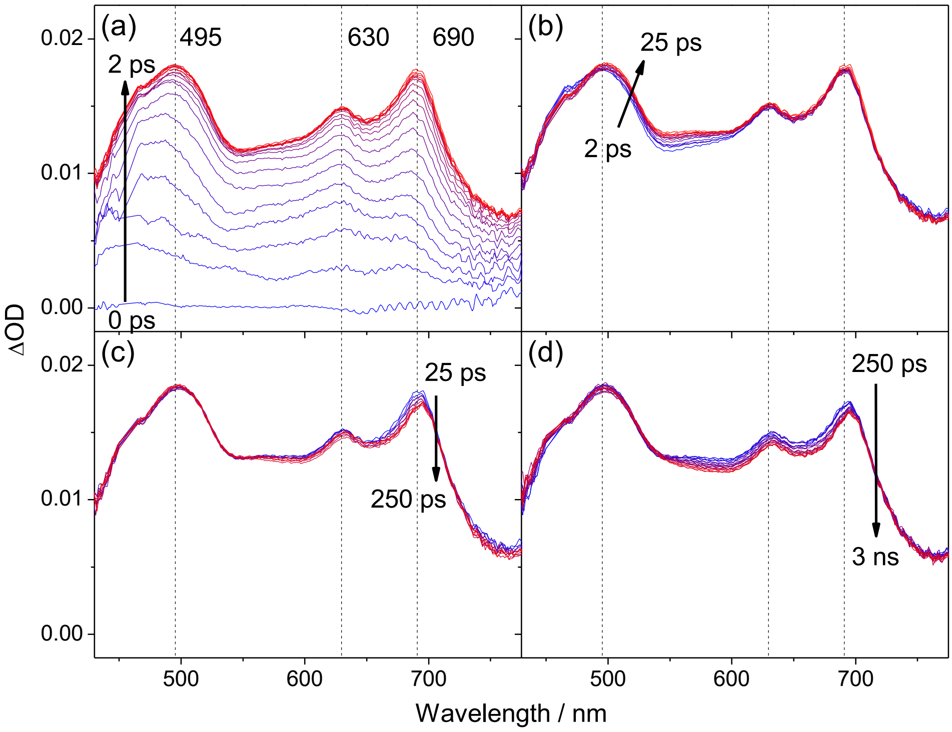

Figure S35. Selected fs-TA spectra of **3** in toluene solution at 188 K recorded at (a) 0–2, (b) 2–25, (c) 25–250, and (d) 250–3000 ps after 400 nm laser excitation.

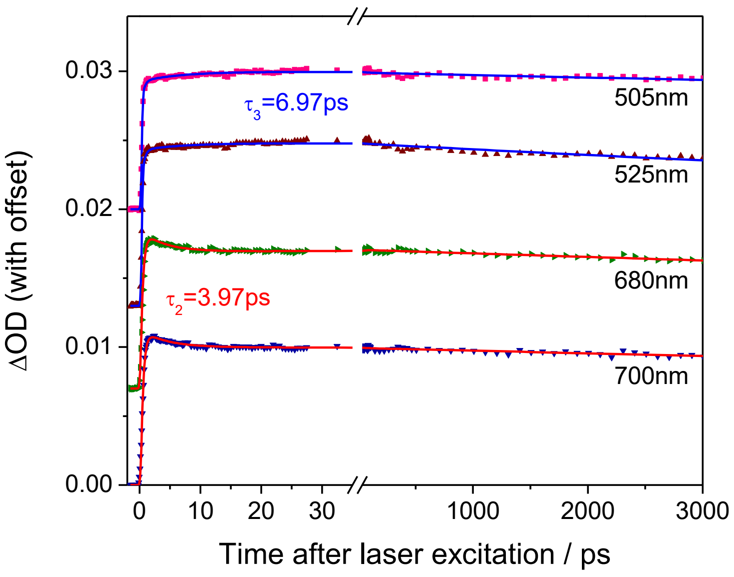

Figure S36. Best fit curves of the kinetic traces of **1** at selected wavelengths in toluene solution at 293 K, with time constants: τ_1 = 0.393 ps, τ_3 = 6.97 ps, τ_4 = 39.4 ps, τ_5 = 45 ns (blue lines) and τ_1 = 0.393 ps, τ_2 = 3.97 ps, τ_4 = 39.4 ps, τ_5 = 45 ns (red lines).

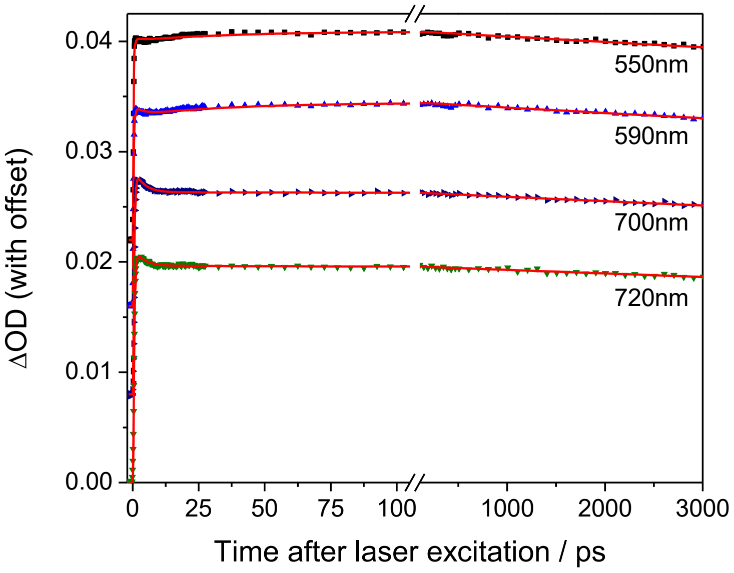

Figure S37. Best fit curves of the kinetic traces of **2** at selected wavelengths in toluene solution at 293 K, with time constants: τ_1 = 0.378 ps, τ_2 = 3.48 ps, τ_3 = 39.3 ps, τ_4 = 46 ns.

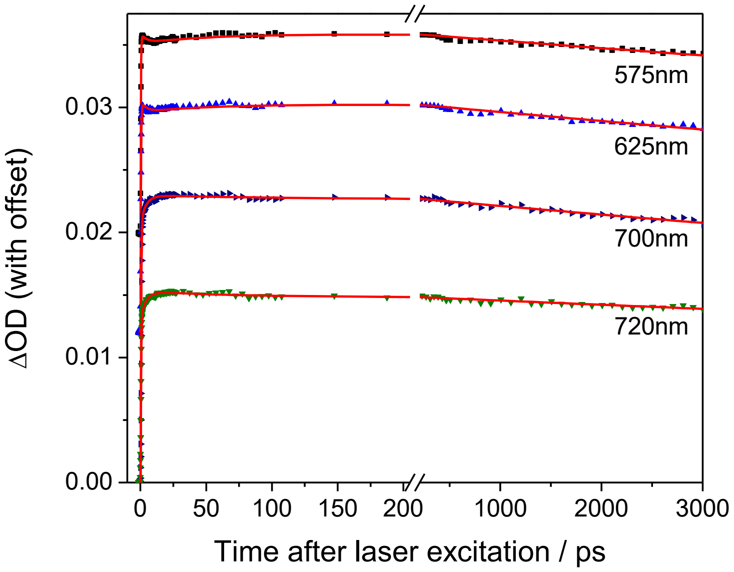

Figure S38. Best fit curves of the kinetic traces of **3** at selected wavelengths in toluene solution at 293 K, with time constants: τ_1 = 0.320 ps, τ_2 = 3.94 ps, τ_3 = 61.1 ps, τ_4 = 37 ns.

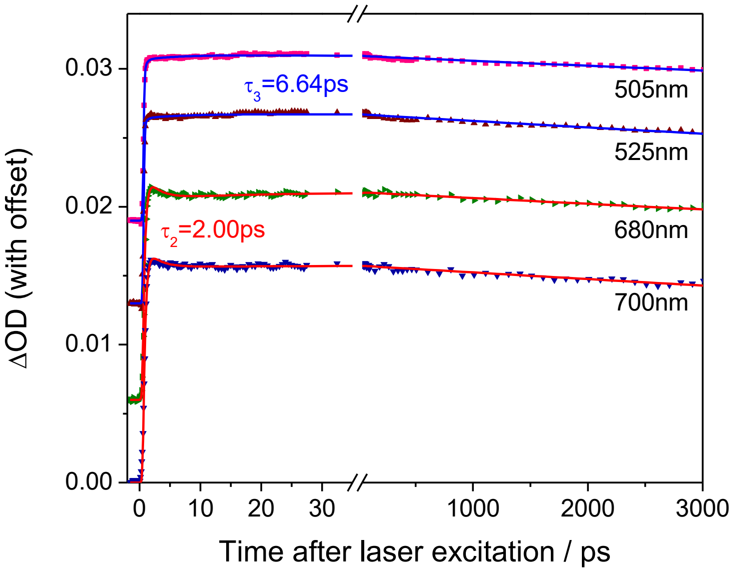

Figure S39. Best fit curves of the kinetic traces of **1** at selected wavelengths in toluene solution at 354 K, with time constants: τ_1 = 0.377 ps, τ_3 = 6.64 ps, τ_4 = 17.3 ps, τ_5 = 34 ns (blue lines) and τ_1 = 0.377 ps, τ_2 = 2.00 ps, τ_4 = 17.3 ps, τ_5 = 34 ns (red lines).

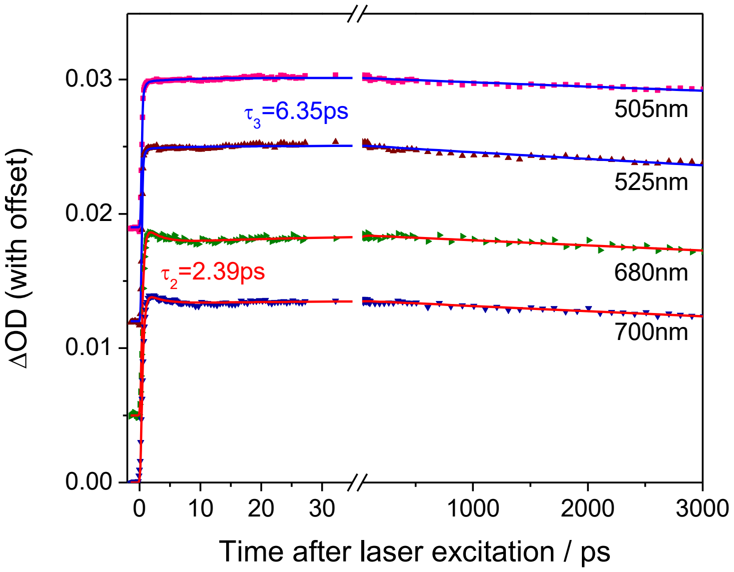

Figure S40. Best fit curves of the kinetic traces of **1** at selected wavelengths in toluene solution at 332 K, with time constants: τ_1 = 0.341 ps, τ_3 = 6.35 ps, τ_4 = 22.7 ps, τ_5 = 34 ns (blue lines) and τ_1 = 0.341 ps, τ_2 = 2.39 ps, τ_4 = 22.7 ps, τ_5 = 34 ns (red lines).

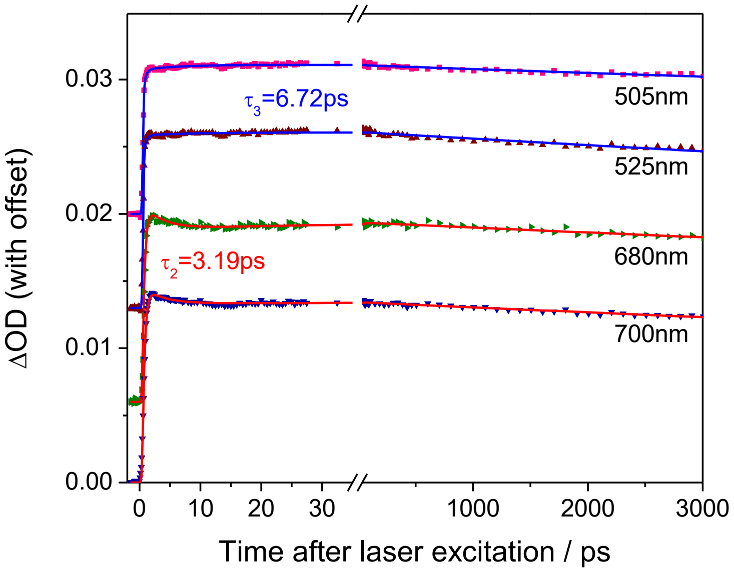

Figure S41. Best fit curves of the kinetic traces of **1** at selected wavelengths in toluene solution at 311 K, with time constants: τ_1 = 0.368 ps, τ_3 = 6.72 ps, τ_4 = 27.0 ps, τ_5 = 36 ns (blue lines) and τ_1 = 0.368 ps, τ_2 = 3.19 ps, τ_4 = 27.0 ps, τ_5 = 36 ns (red lines).

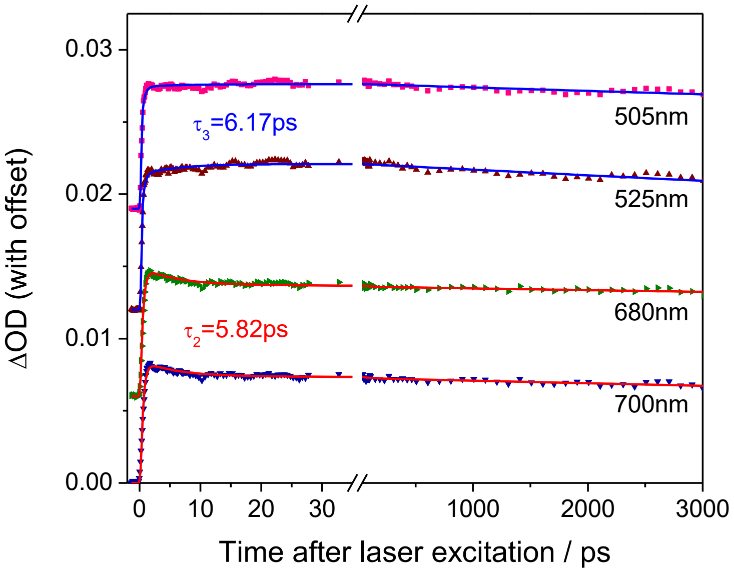

Figure S42. Best fit curves of the kinetic traces of **1** at selected wavelengths in toluene solution at 258 K, with time constants: τ_1 = 0.419 ps, τ_3 = 6.17 ps, τ_4 = 78.3 ps, τ_5 = 60 ns (blue lines) and τ_1 = 0.419 ps, τ_2 = 5.82 ps, τ_4 = 78.3 ps, τ_5 = 60 ns (red lines).

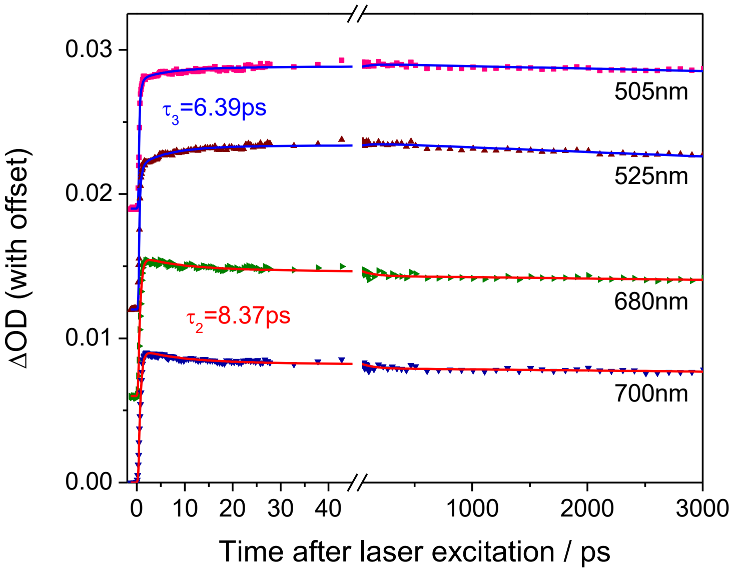

Figure S43. Best fit curves of the kinetic traces of **1** at selected wavelengths in toluene solution at 238 K, with time constants: τ_1 = 0.425 ps, τ_3 = 6.39 ps, τ_4 = 106.8 ps, τ_5 = 76 ns (blue lines) and τ_1 = 0.425 ps, τ_2 = 8.37 ps, τ_4 = 106.8 ps, τ_5 = 76 ns (red lines).

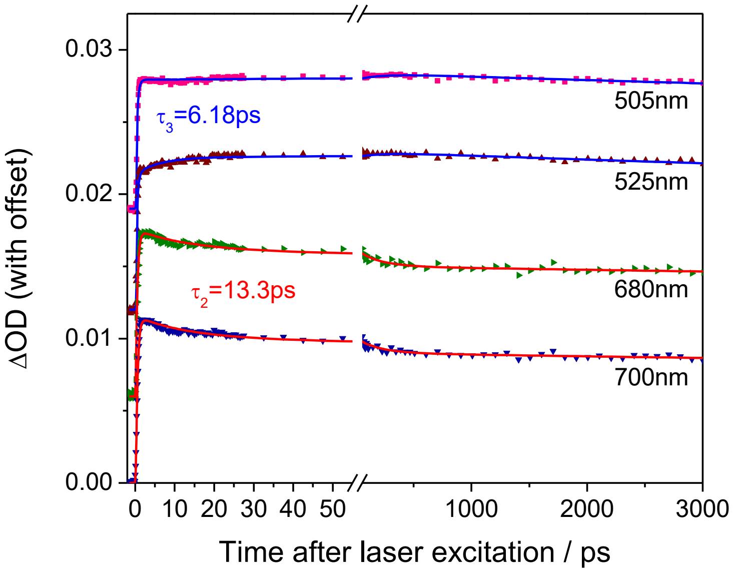

Figure S44. Best fit curves of the kinetic traces of **1** at selected wavelengths in toluene solution at 213 K, with time constants: τ_1 = 0.424 ps, τ_3 = 6.18 ps, τ_4 = 195.0 ps, τ_5 = 85 ns (blue lines) and τ_1 = 0.424 ps, τ_2 = 13.3 ps, τ_4 = 195.0 ps, τ_5 = 85 ns (red lines).

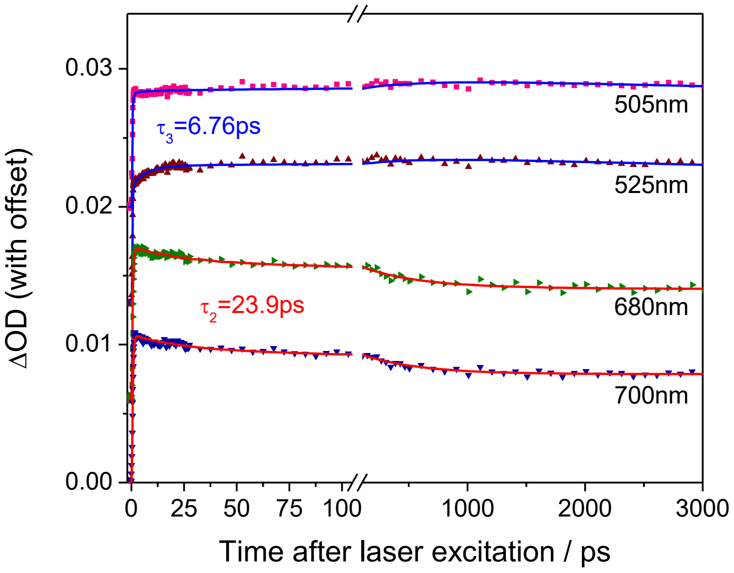

Figure S45. Best fit curves of the kinetic traces of **1** at selected wavelengths in toluene solution at 188 K, with time constants: τ_1 = 0.432 ps, τ_3 = 6.76 ps, τ_4 = 487.4 ps, τ_5 = 180 ns (blue lines) and τ_1 = 0.432 ps, τ_2 = 23.9 ps, τ_4 = 487.4 ps, τ_5 = 180 ns (red lines).

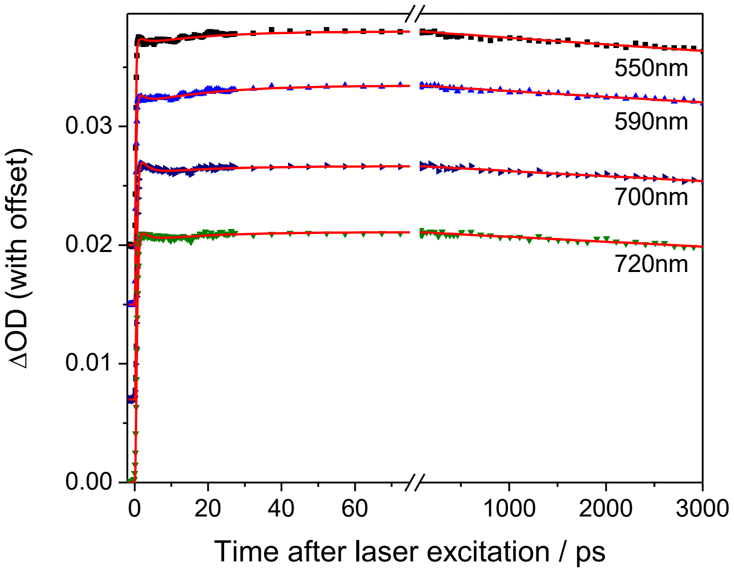

Figure S46. Best fit curves of the kinetic traces of **2** at selected wavelengths in toluene solution at 354 K, with time constants: $\tau_1 = 0.382$ ps, $\tau_2 = 2.21$ ps, $\tau_3 = 19.1$ ps, $\tau_4 = 40$ ns.

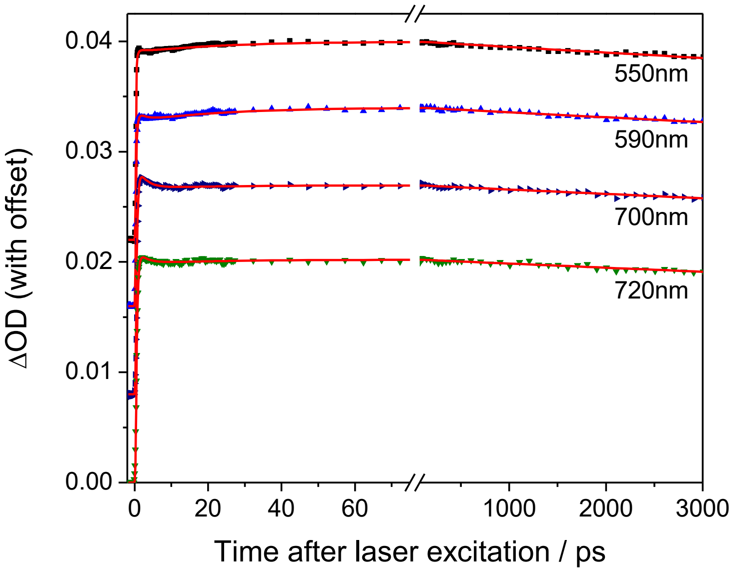

Figure S47. Best fit curves of the kinetic traces of **2** at selected wavelengths in toluene solution at 332 K, with time constants: $\tau_1 = 0.361$ ps, $\tau_2 = 2.42$ ps, $\tau_3 = 24.1$ ps, $\tau_4 = 42$ ns.

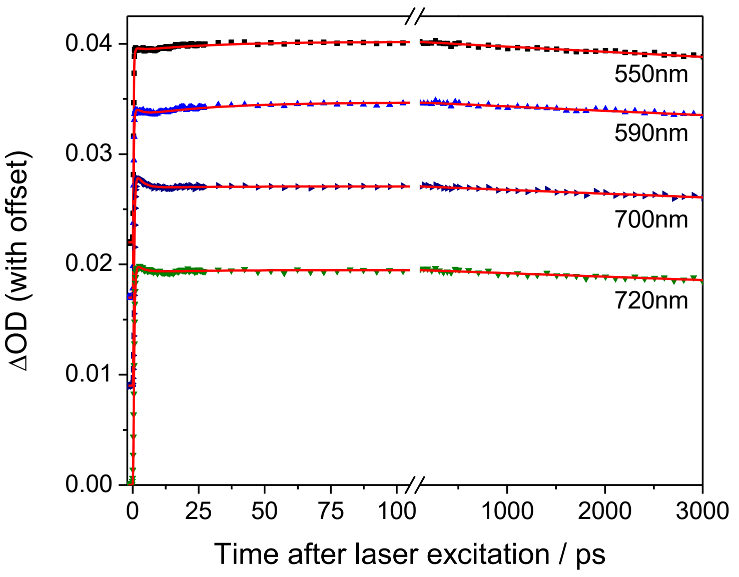

Figure S48. Best fit curves of the kinetic traces of **2** at selected wavelengths in toluene solution at 311 K, with time constants: τ_1 = 0.355 ps, τ_2 = 2.72 ps, τ_3 = 30.1 ps, τ_4 = 45 ns.

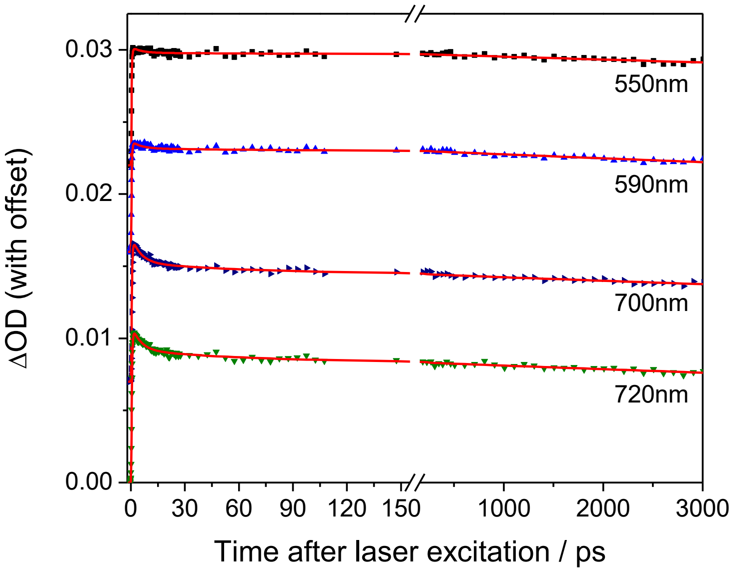

Figure S49. Best fit curves of the kinetic traces of **2** at selected wavelengths in toluene solution at 258 K, with time constants: τ_1 = 0.357 ps, τ_2 = 5.09 ps, τ_3 = 64.0 ps, τ_4 = 49 ns.

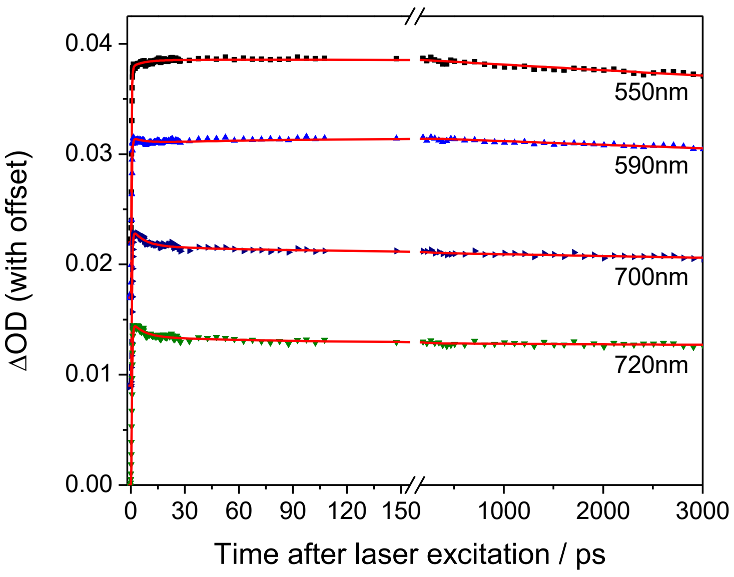

Figure S50. Best fit curves of the kinetic traces of **2** at selected wavelengths in toluene solution at 238 K, with time constants: τ_1 = 0.369 ps, τ_2 = 6.86 ps, τ_3 = 90.5 ps, τ_4 = 67 ns.

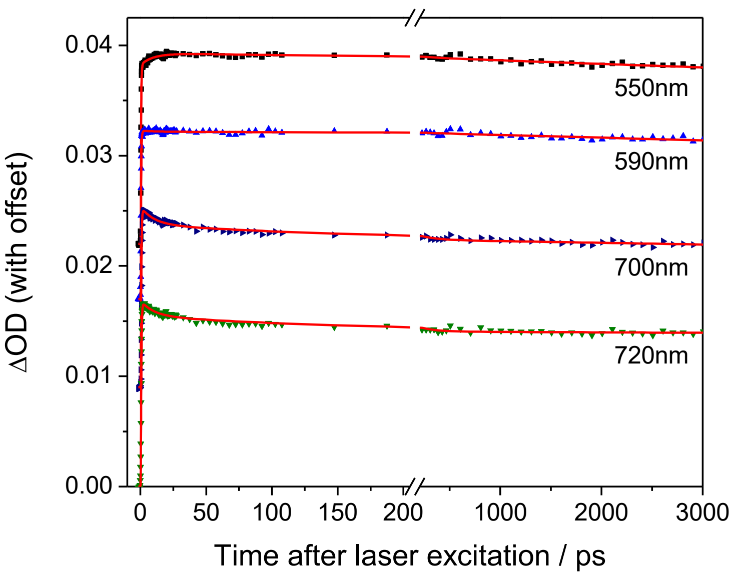

Figure S51. Best fit curves of the kinetic traces of **2** at selected wavelengths in toluene solution at 213 K, with time constants: τ_1 = 0.384 ps, τ_2 = 9.85 ps, τ_3 = 158.5 ps, τ_4 = 95 ns.

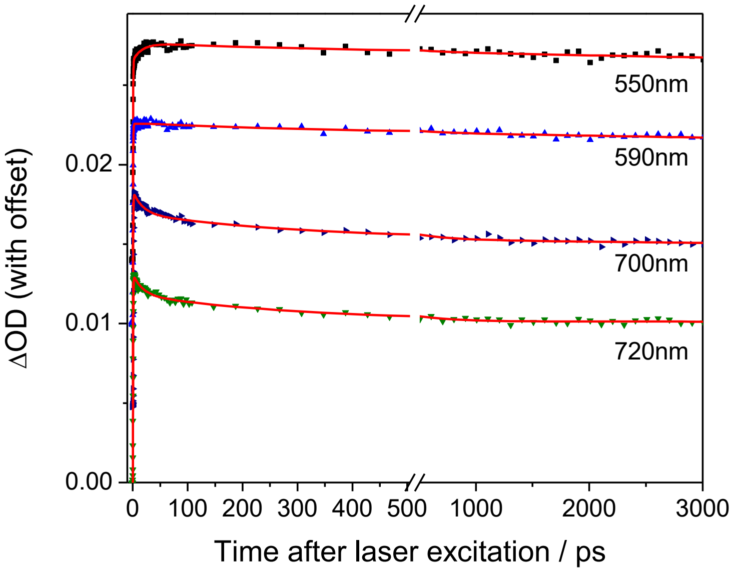

Figure S52. Best fit curves of the kinetic traces of **2** at selected wavelengths in toluene solution at 188 K, with time constants: τ_1 = 0.415 ps, τ_2 = 17.9 ps, τ_3 = 309.4 ps, τ_4 = 160 ns.

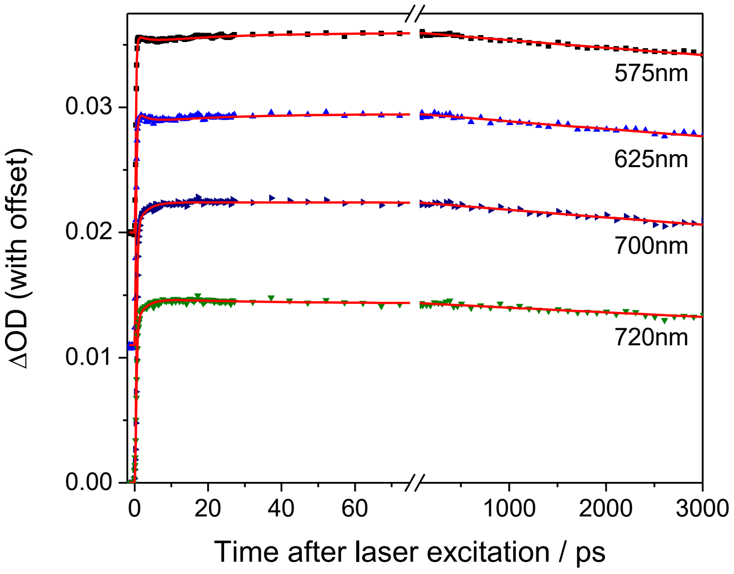

Figure S53. Best fit curves of the kinetic traces of **3** at selected wavelengths in toluene solution at 354 K, with time constants: τ_1 = 0.296 ps, τ_2 = 2.66 ps, τ_3 = 24.7 ps, τ_4 = 33 ns.

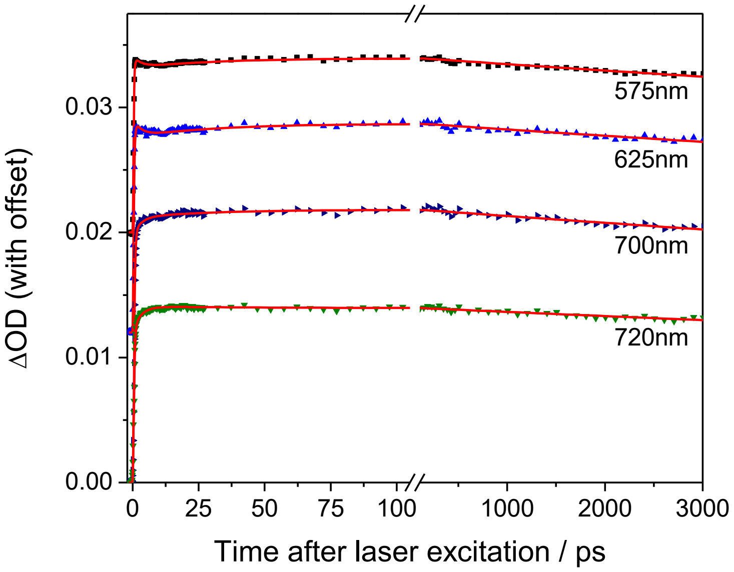

Figure S54. Best fit curves of the kinetic traces of **3** at selected wavelengths in toluene solution at 332 K, with time constants: τ_1 = 0.331 ps, τ_2 = 2.92 ps, τ_3 = 33.1 ps, τ_4 = 34 ns.

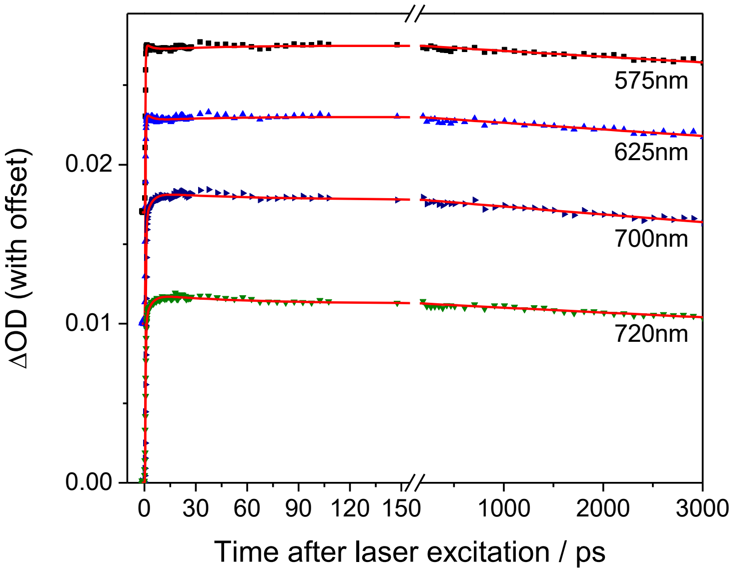

Figure S55. Best fit curves of the kinetic traces of **3** at selected wavelengths in toluene solution at 310 K, with time constants: τ_1 = 0.326 ps, τ_2 = 3.40 ps, τ_3 = 47.0 ps, τ_4 = 35 ns.

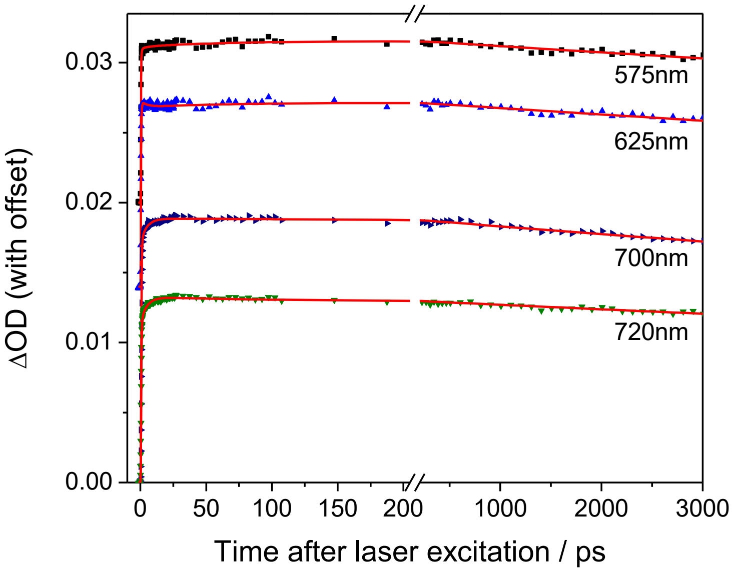

Figure S56. Best fit curves of the kinetic traces of **3** at selected wavelengths in toluene solution at 273 K, with time constants: τ_1 = 0.334 ps, τ_2 = 4.70 ps, τ_3 = 75.3 ps, τ_4 = 37 ns.

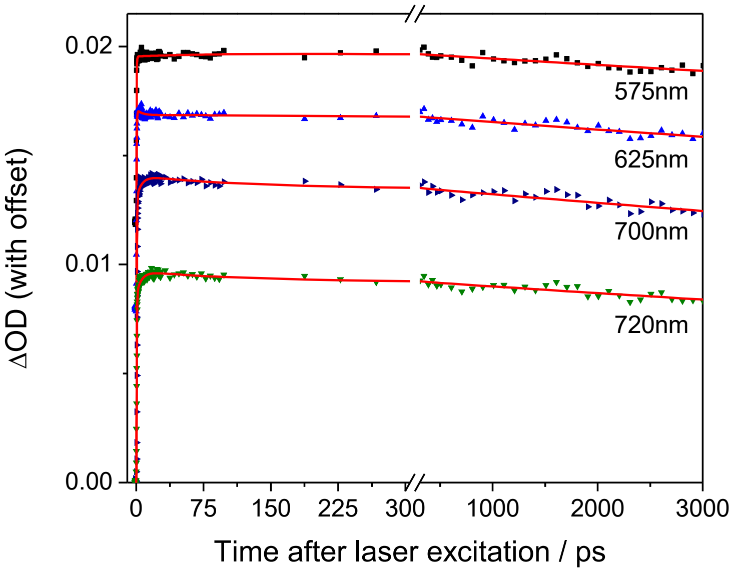

Figure S57. Best fit curves of the kinetic traces of **3** at selected wavelengths in toluene solution at 258 K, with time constants: τ_1 = 0.353 ps, τ_2 = 5.76 ps, τ_3 = 99.0 ps, τ_4 = 38 ns.

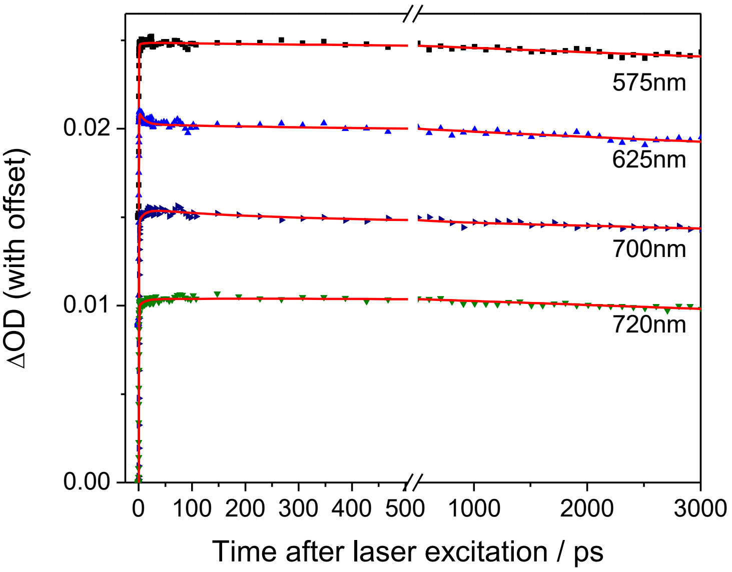

Figure S58. Best fit curves of the kinetic traces of **3** at selected wavelengths in toluene solution at 213 K, with time constants: $\tau_1 = 0.341$ ps, $\tau_2 = 10.4$ ps, $\tau_3 = 244.7$ ps, $\tau_4 =$ 67 ns.

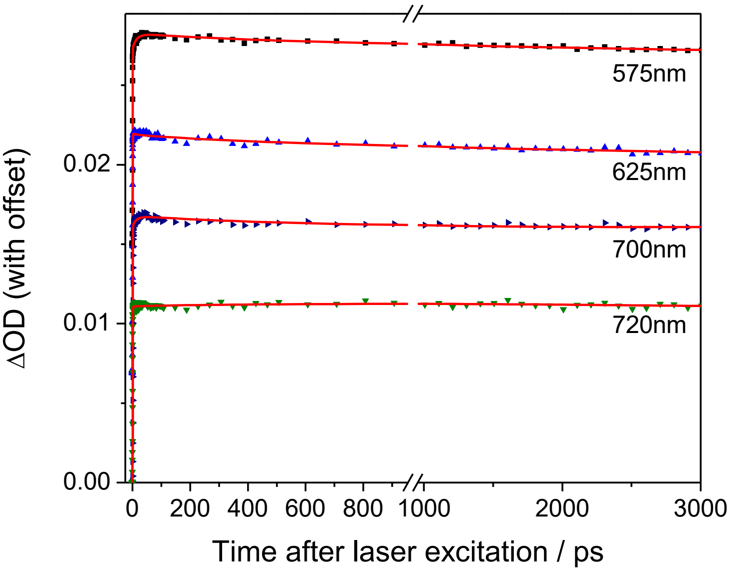

Figure S59. Best fit curves of the kinetic traces of **3** at selected wavelengths in toluene solution at 188 K, with time constants: $\tau_1 = 0.376$ ps, $\tau_2 = 14.4$ ps, $\tau_3 = 568.6$ ps, $\tau_4 =$ 106 ns.

Table S10. A summary of kinetic profiles[a] of different exponential components observed in TA spectra of **1**–**3** at different temperatures

1

T / K	τ_1 / ps	τ_2[b] / ps	τ_3[c] / ps	τ_4 / ps	τ_5[d] / ns
353.65	0.377±0.078	2.00±0.10	6.64±0.24	17.3±2.8	>20
332.35	0.341±0.095	2.39±0.20	6.35±0.74	22.7±1.2	>20
310.75	0.368±0.072	3.19±0.17	6.72±0.75	27.0±1.7	>20
293.15	0.393±0.095	3.97±0.23	6.97±0.45	39.4±3.4	>20
258.15	0.419±0.077	5.82±0.53	6.17±0.59	78.3±9.7	>20
238.15	0.425±0.067	8.37±0.85	6.39±0.54	106.8±17.1	>20
213.15	0.424±0.067	13.3±1.33	6.18±0.79	195.0±21.2	>20
188.15	0.432±0.061	23.9±1.77	6.76±0.83	487.4±41.7	>20

2[e]

T / K	τ_1 / ps	τ_2 / ps	τ_3 / ps	τ_4[d] / ns
353.65	0.382±0.042	2.21±0.13	19.1±0.8	>20
332.35	0.361±0.051	2.42±0.08	24.1±1.3	>20
310.75	0.355±0.051	2.72±0.18	30.1±1.3	>20
293.15	0.378±0.039	3.48±0.13	39.3±1.2	>20
258.15	0.357±0.030	5.09±0.72	64.0±3.3	>20
238.15	0.369±0.038	6.86±0.59	90.5±6.0	>20
213.15	0.384±0.030	9.85±1.51	158.5±18.7	>20
188.15	0.415±0.029	17.9±2.00	309.4±22.8	>20

3[f]

T / K	τ_1 / ps	τ_2 / ps	τ_3 / ps	τ_4[d]/ ns
353.65	0.296±0.046	2.66±0.17	24.7±1.8	>20
332.35	0.331±0.047	2.92±0.07	33.1±2.3	>20
310.75	0.326±0.044	3.40±0.24	47.0±4.7	>20
293.15	0.320±0.039	3.94±0.19	61.1±3.3	>20
273.15	0.334±0.050	4.70±0.37	75.3±4.9	>20
258.15	0.353±0.070	5.76±0.78	99.0±17.6	>20
213.15	0.341±0.038	10.4±0.83	244.7±26.8	>20
188.15	0.376±0.032	14.4±1.32	568.6±50.2	>20

[a] The error bars are calculated from the variations from multiple measurements and the fitting errors.

[b] Determined from the spectral changes in the wavelength range of 680–700 nm.

[c] Determined from the spectral changes in the wavelength range of 500–550 nm.

[d] Cannot be accurately determined as the time constants are beyond the instrument time window.

[e] Determined by global fitting across the entire wavelength range, with the initial estimates for τ_2 and τ_3 based on the spectral changes observed in the wavelength ranges of 700–730 nm and 540–600 nm, respectively.

[f] Determined by global fitting across the entire wavelength range, with the initial estimates for τ_2 and τ_3 based on the spectral changes observed in the wavelength ranges of 700–730 nm and 560–630 nm, respectively.

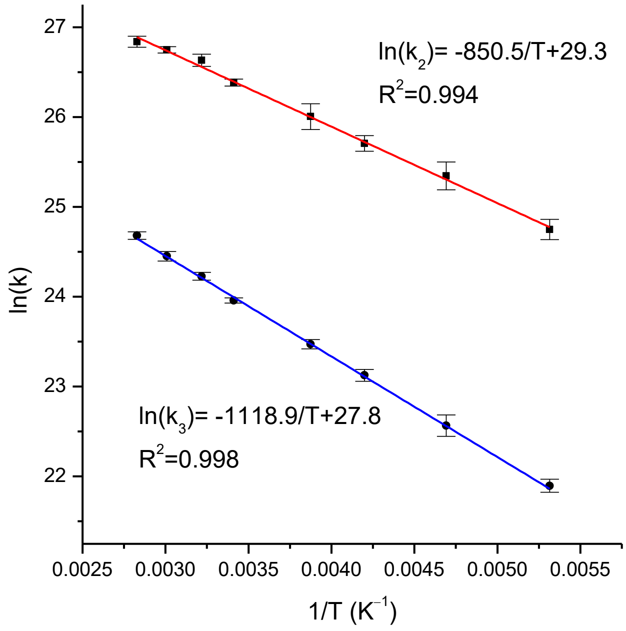

Figure S60. Arrhenius plots of k_2 (τ_2) and k_3 (τ_3) from kinetics analysis of transient absorption spectra of **2**. Among the processes observed in the ps regime, the processes associated with τ_2 and τ_3 show temperature dependence, and the calculated E_a for the processes are +7.1 ± 0.2 kJ mol^{-1} (74 ± 2 meV) and +9.3 ± 0.2 kJ mol^{-1} (96 ± 2 meV).

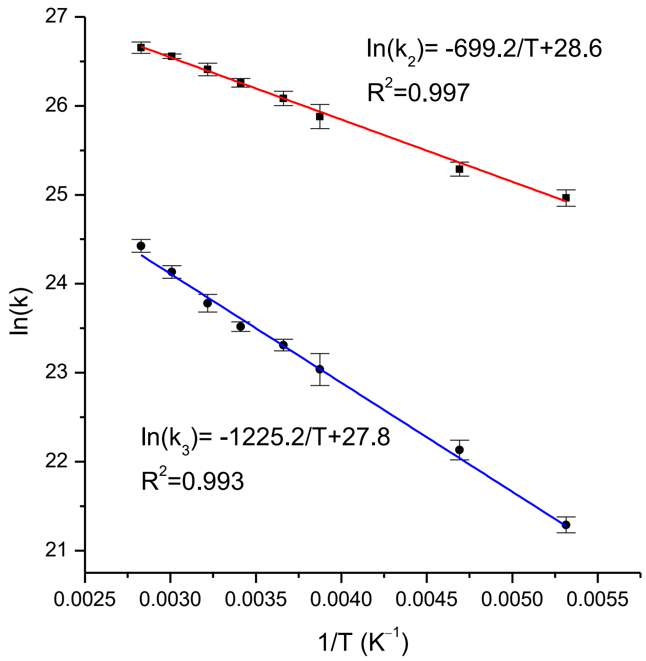

Figure S61. Arrhenius plots of k_2 (τ_2) and k_3 (τ_3) from kinetics analysis of transient absorption spectra of **3**. Among the processes observed in the ps regime, the processes associated with τ_2 and τ_3 show temperature dependence, and the calculated E_a for the processes are +5.8 ± 0.1 kJ mol^{-1} (60 ± 1 meV) and +10.2 ± 0.3 kJ mol^{-1} (106 ± 3 meV).

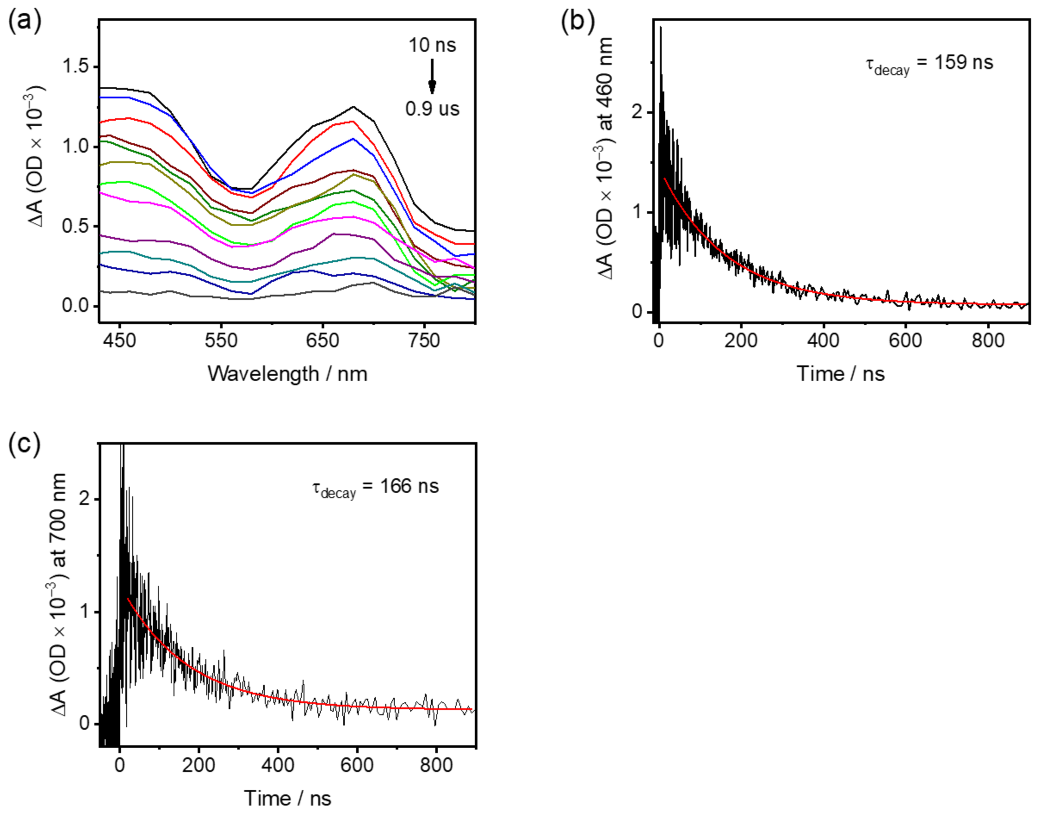

Figure S62. (a) ps-TA spectra of **3** in degassed toluene at 298 K at decay times of 0.01–0.9 μs and the decay traces monitored at (b) 460 nm and (c) 700 nm.

Electrochemical Properties

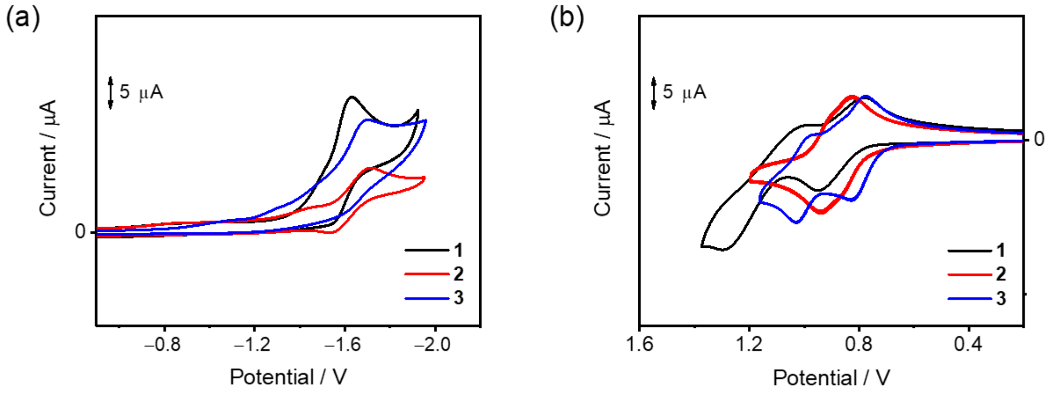

Figure S63. Cyclic voltammograms showing the (a) reductive and (b) oxidative scans of **1**–**3** in tetrahydrofuran (0.1 M ^{n}Bu$_4$NPF$_6$).

Table S11. Electrochemical data of **1**–**3**[a]

Compound	Oxidation $E_{1/2}^{ox}$ / V vs SCE[b] (ΔE_p / mV)[c] [E_{pa} / V vs SCE]	Reduction [E_{pc} / V vs SCE]	HOMO / eV[d]	LUMO / eV[d]
**1**	+0.85 (73), [+1.17]	[−1.55]	−5.09	−2.69
**2**	+0.84 (64), [+0.86]	[−1.57]	−5.08	−2.67
**3**	+0.80 (61), +1.00 (64)	[−1.61]	−5.04	−2.63

[a] Measured in THF solution with 0.1 M ^{n}Bu$_4$NPF$_6$ (TBAH) as supporting electrolyte at 298 K; scan rate = 100 mV s^{-1}.

[b] $E_{1/2} = (E_{pa} + E_{pc})/2$; E_{pa} and E_{pc} are the peak anodic and peak cathodic potentials, respectively.

[c] $\Delta E_p = (E_{pa} - E_{pc})$.

[d] E_{HOMO} and E_{LUMO} levels were calculated from electrochemical potentials, i.e. $E_{HOMO} = -[E_{1/2}^{ox}$ (vs Fc$^+$/Fc) + 4.80] eV; $E_{LUMO} = -[E_{1/2}^{red}$ (vs Fc$^+$/Fc) + 4.80] eV. E^o(Fc$^+$/Fc) = +0.56 V vs SCE in THF (0.1 M ^{n}Bu$_4$NPF$_6$).

Computational Studies

Geometric optimization with density functional theory (DFT) and time-dependent DFT (TDDFT) methods has been performed using Gaussian 16 (Revision A.03) package.[7] The ground-state (S_0) geometries of **1**–**3** were fully optimized in toluene at PBE0/6-31g(d,p)/SDD level,[8] in connection with the polarizable continuum model (PCM).[9] The noncovalent interactions were analyzed on both crystal structures and optimized S_0 geometries using NCIPLOT.[10] TDDFT calculations at the same level were carried out on the optimized S_0 geometries to obtain the singlet excited states of **1**–**3**. The singlet-singlet transitions in the electronic absorption spectra were simulated using Multiwfn package.[11] To derive more accurate electronic energies, single point calculations using SDD pseudopotential and basis set for gold and 6-311g(3df,3dp) basis set for other atoms have been performed on the optimized structures. The simulated absorption spectra and the first five singlet excited states of the complexes are depicted in Figures S46–S48 and Table S14. To gain more insights into the unique TADF-TSDP property, we have optimized the geometric structures of emissive states, including S_1, T_1 and potential T_1' (with ^{3}IL and ^{3}CT characters) states. The electron-hole and inter-fragment charge transfer (IFCT) analysis were performed for S_1, T_1 and potential T_1' states of **1**–**3** using Multiwfn package. The definition for the molecular fragment can be found in Figure S73. The Cartesian coordinates of the optimized structures of **1**–**3** are given in Tables S20–S34.

The energies of the frontier molecular orbitals of **1**–**3** are depicted in Figures S43–S45. The computed HOMO energy increases on going from **1** (–5.09 eV) to **2** (–5.06 eV) to **3** (–5.04 eV), due to the destabilization of the HOMO by increasing the electron-donating strength of the lateral arylamine on the carbazolyl auxiliary ligand from DMAC to PXO to DPXO. A similar trend is also observed for the LUMO energy levels of **1** (–2.12 eV), **2** (–2.10 eV) and **3** (–2.07 eV) as the result of the destabilization of the π* orbital localized on the C^C^N ligand. The electrostatic potential surface analysis (Figure S46) demonstrates the enhancement of electron densities on carbazolyl unit and

the lateral arylamine in the order of **1** < **2** < **3**, in line with the increasing contribution of the lateral arylamine to the HOMO composition. The improved donor strength of the carbazolyl *N*-donor ligand results in a more electron-rich metal center, which destabilizes the metal d_{π} orbitals and in turn would lead to a larger destabilization of π^* orbital localized on the the C^C^N ligand. Overall, the trends of HOMO and LUMO energies and HOMO–LUMO gap are in excellent agreement with the electrochemical studies (Table S11).

We have further computed the rate constants (Table S15) for the reverse intersystem crossing (k_{RISC}) and reverse internal conversion (k_{RIC}) processes following the computational protocols described in our previous work.[12] For **2** and **3**, the $T_1 \rightarrow T_1'$ (^{3}CT) RIC process is faster than the RISC process at room temperature while for **1**, the rate constant for the $T_1 \rightarrow T_1'$ (^{3}IL) RIC process is in the same order of magnitude as k_{RISC}. Our results confirm that RIC processes are indeed efficient. It is believed that the ^{3}IL state of **1**, the ^{3}IL and ^{3}TSCT states of **2**, and the ^{3}IL and mixed ^{3}TSCT/^{3}TBCT states of **3** are involved in the emission process. To investigate the potential impacts of excited state geometries and noncovalent interactions on the k_{RIC} values, we have also estimated the energy gaps among low-lying triplet states by ΔE_{T2-T1} and ΔE_{T3-T1} computed using the solid state crystal structures of **1**–**3** (Table S16). Here, triplet states that are higher-lying than the T_1 state were labelled using the T_2 and T_3 states rather than the T_1' state, since the T_2 and T_3 states were computed based on the same geometry (solid state crystal structure) as the T_1 state. Despite the differences in noncovalent interactions between solid and solution phases, the computed ΔE_{T2-T1} and ΔE_{T3-T1} in the solid state (ranging from 139 to 438 meV) are comparable to the computed $\Delta E_{T1'-T1}$ in toluene (ranging from 220 to 498 meV, Table S14). The computed k_{RIC} is heavily influenced by the energy gap between triplet states. Thus, we anticipate that k_{RIC} in the solid and solution phases would be in a similar order of magnitude.

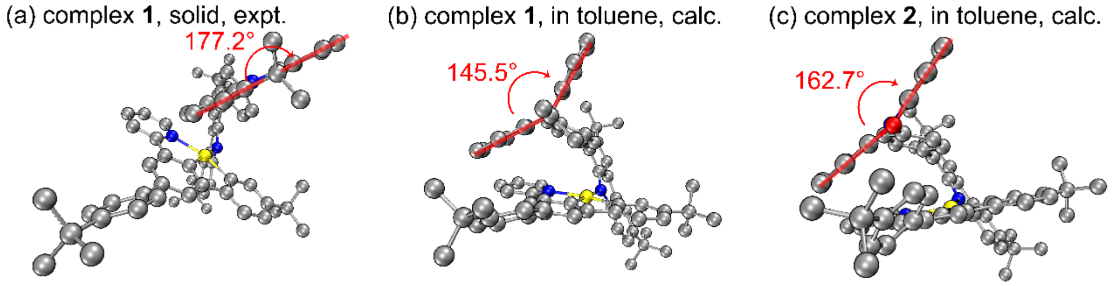

Figure S64. (a) Perspective view of the crystal structure of **1** and optimized S_0 geometries of (b) **2** and (c) **3** in toluene showing the dihedral angle between two phenyl rings.

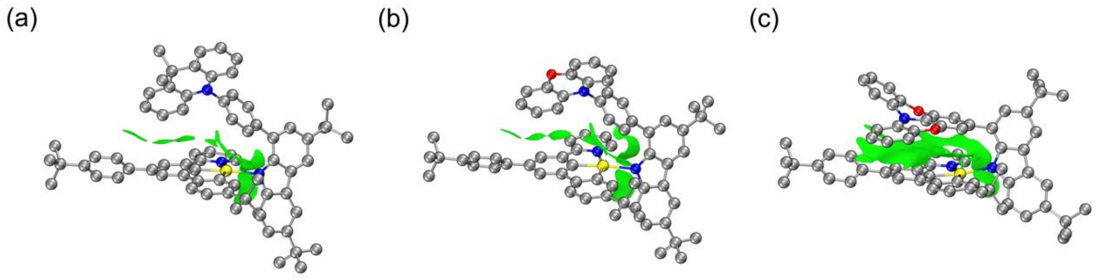

Figure S65. Isosurfaces of the noncovalent interaction for (a) **1**, (b) **2** and (c) **3** in the solid state crystal.

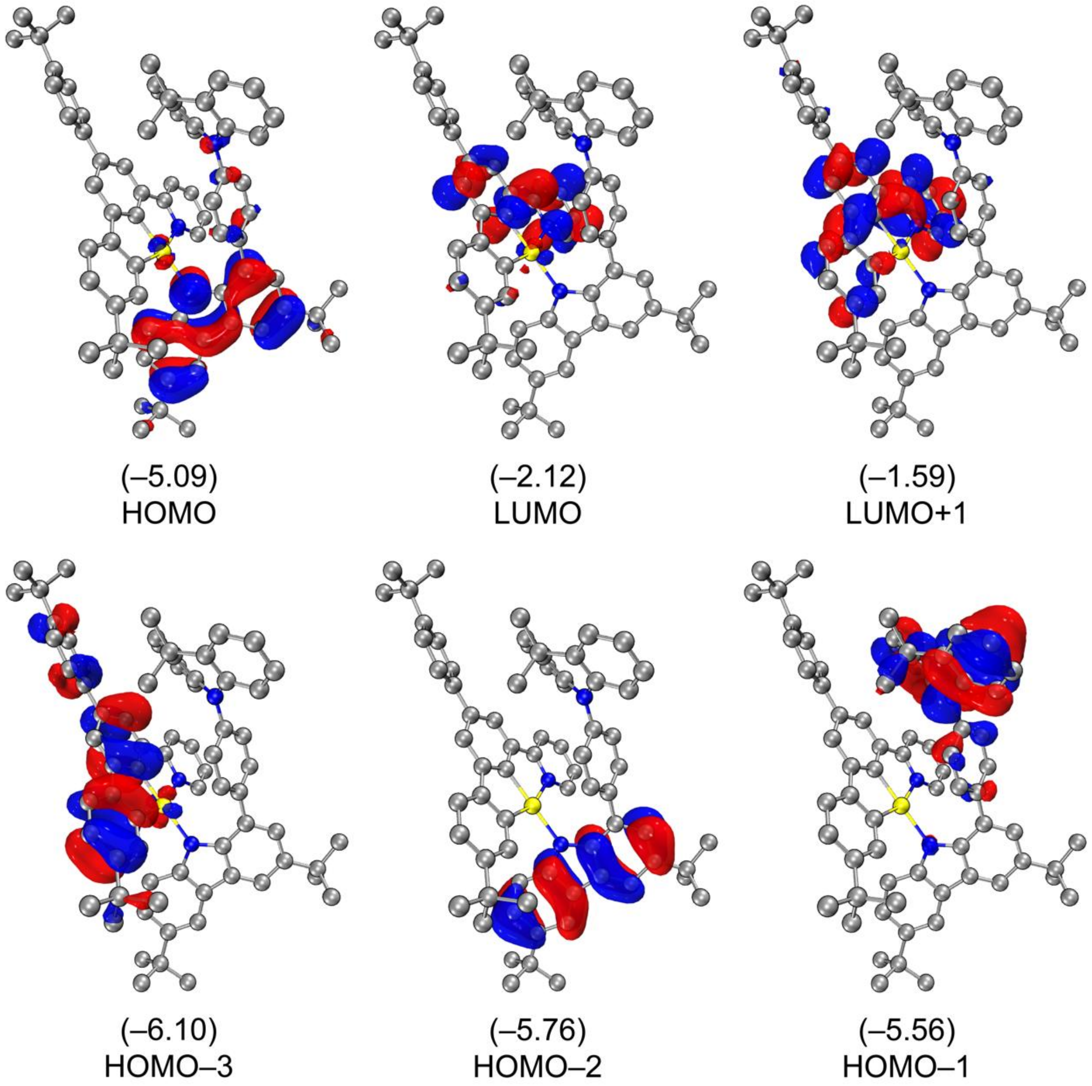

Figure S66. Spatial plots (isovalue = 0.03) of selected frontier molecular orbitals of **1** obtained from the PBE0/PCM (toluene) calculation. MO energies are provided in parentheses.

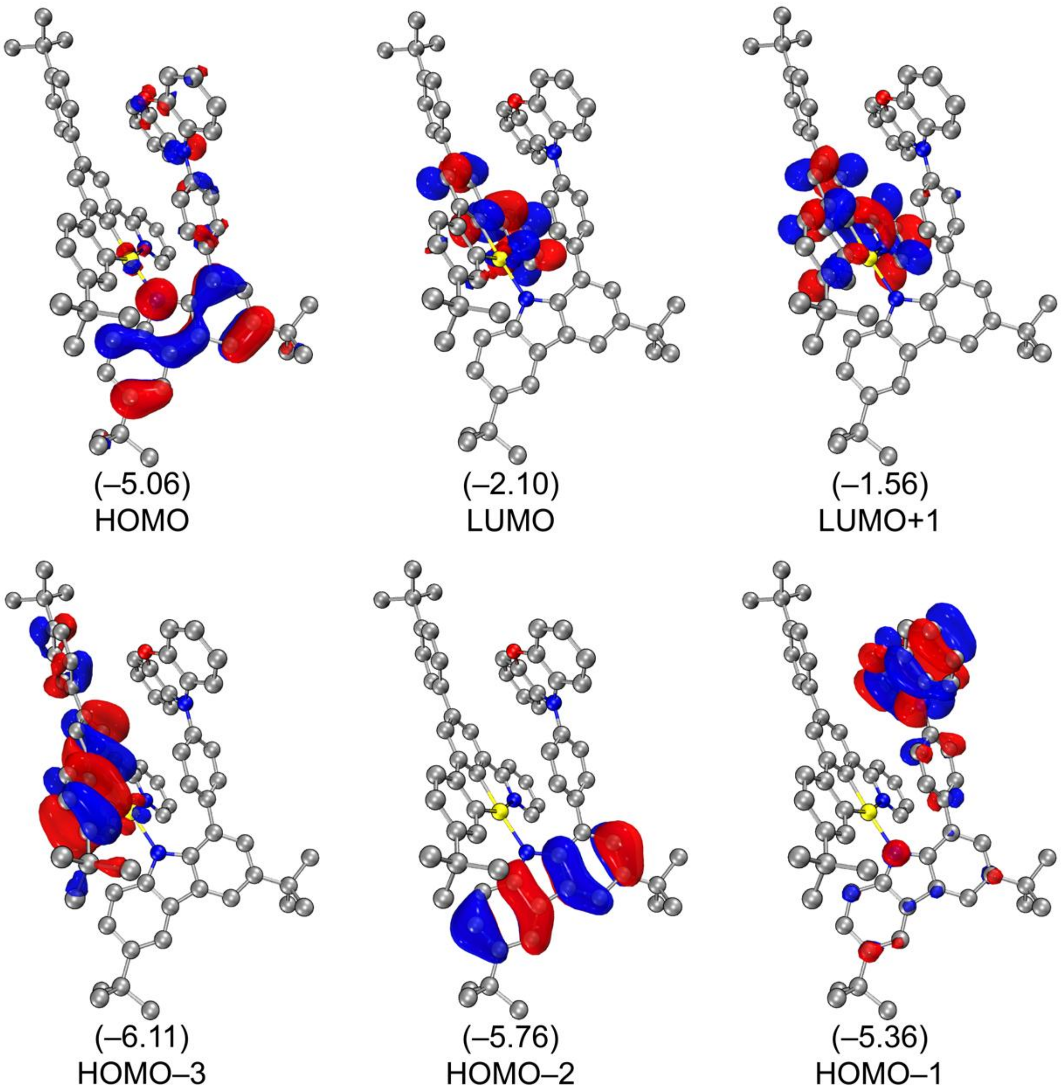

Figure S67. Spatial plots (isovalue = 0.03) of selected frontier molecular orbitals of **2** obtained from the PBE0/PCM (toluene) calculation. MO energies are provided in parentheses.

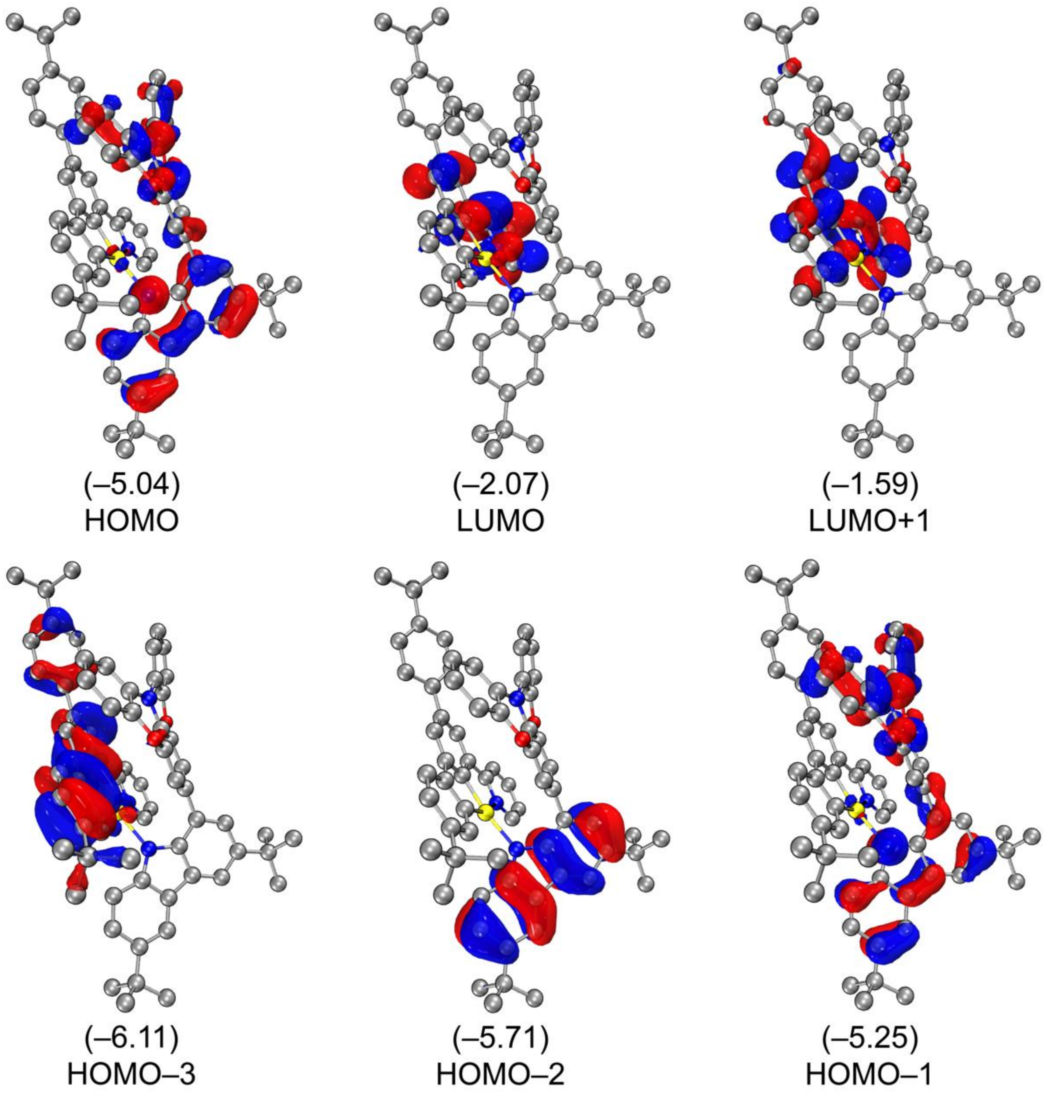

Figure S68. Spatial plots (isovalue = 0.03) of selected frontier molecular orbitals of **3** obtained from the PBE0/PCM (toluene) calculation. MO energies are provided in parentheses.

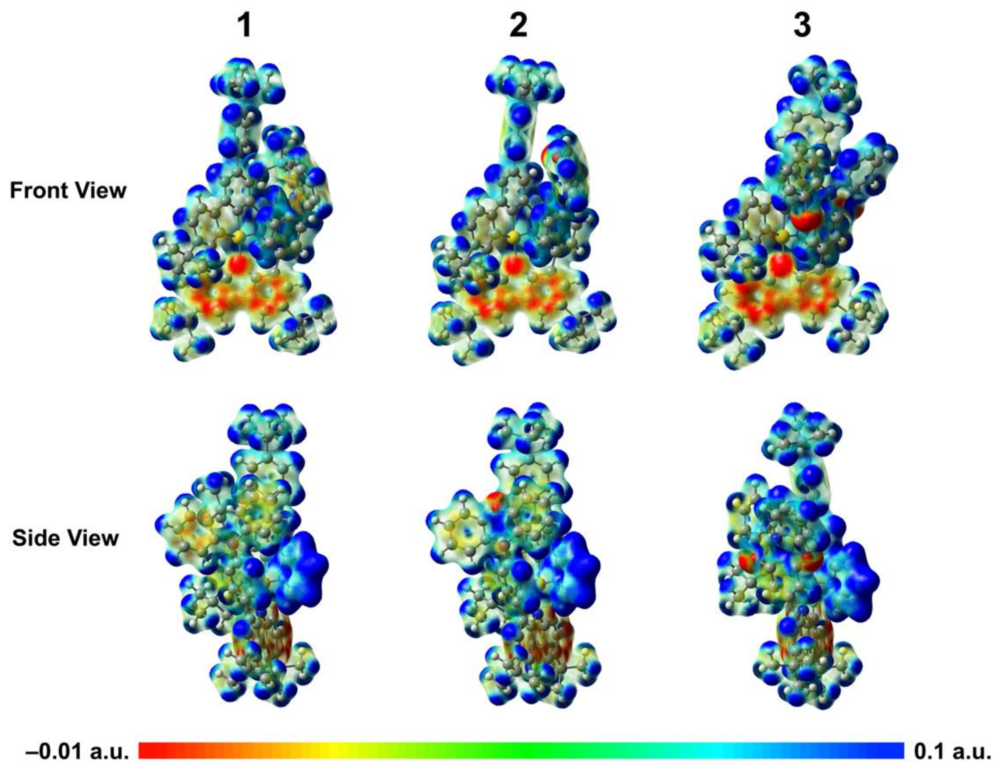

Figure S69. Calculated electrostatic potential surfaces (isovalue = 0.02) using the ground-state geometries of **1–3**.

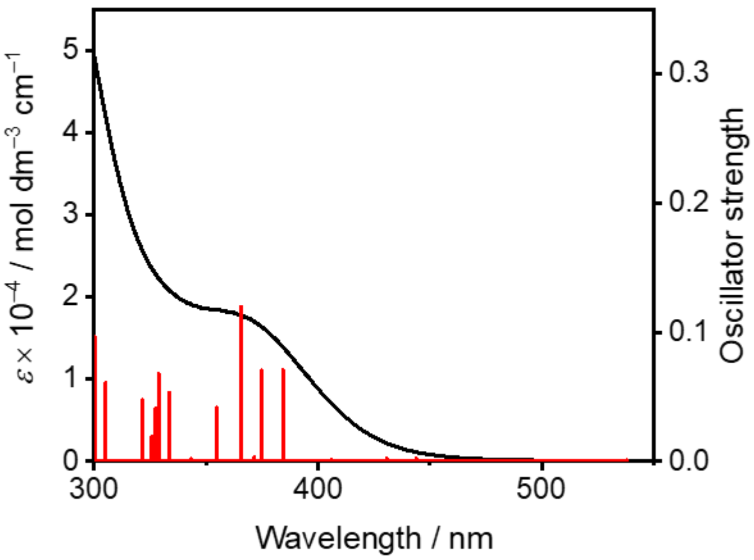

Figure S70. Simulated absorption spectrum of **1**. The vertical lines refer to the unbroadened oscillator strengths of the singlet–singlet transitions.

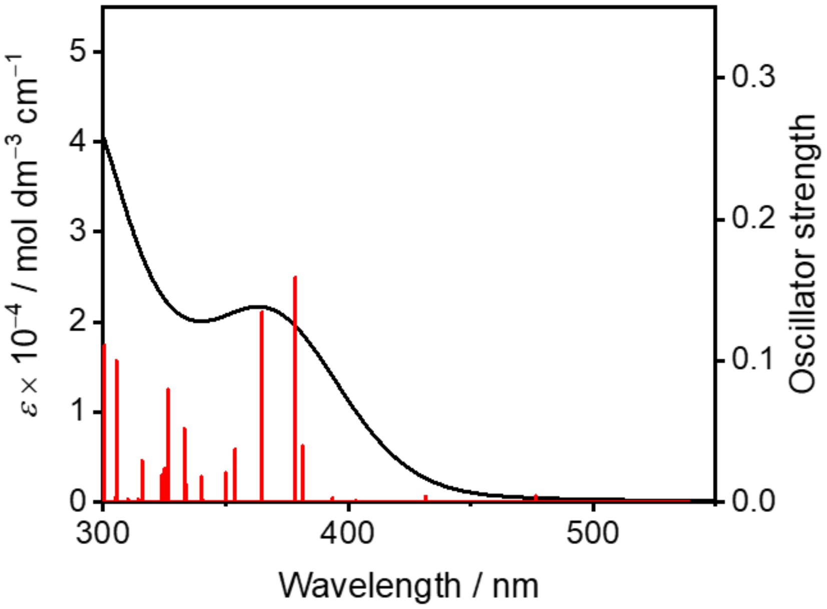

Figure S71. Simulated absorption spectrum of **2**. The vertical lines refer to the unbroadened oscillator strengths of the singlet–singlet transitions.

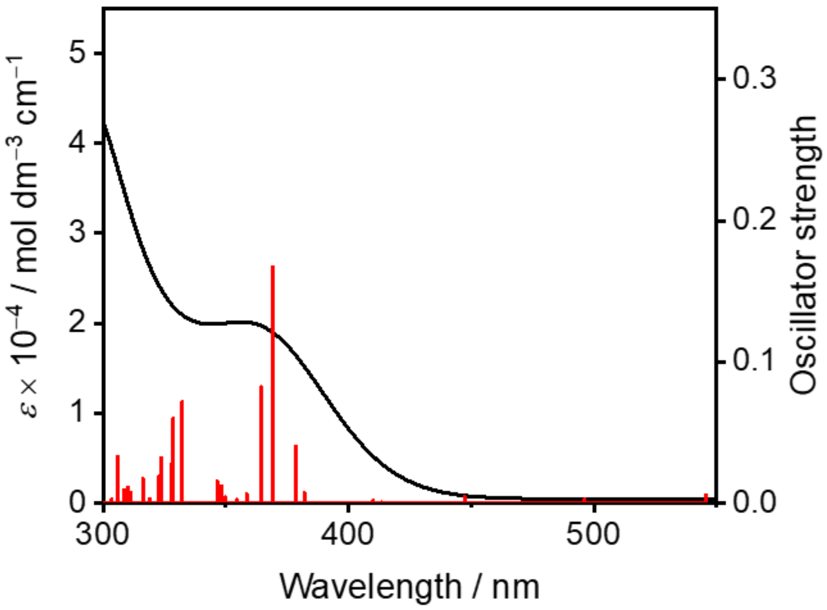

Figure S72. Simulated absorption spectrum of **3**. The vertical lines refer to the unbroadened oscillator strengths of the singlet–singlet transitions.

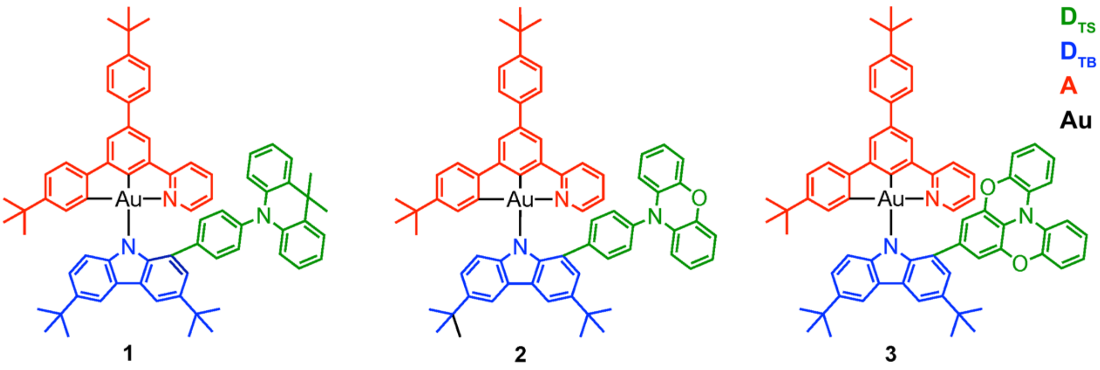

Figure S73. Molecular fragments of complexes **1–3** defined for IFCT analysis.

Table S12. The first five singlet (S_{1-5}) excited states computed by TDDFT/PCM (toluene) at the optimized ground-state geometries

Complex	S_n	Excitation[a] (Coefficient)[b]	λ^c / nm	f^d
**1**	S_1	H→L (0.70)	538	0.001
	S_2	H–1→L (0.70)	444	0.003
	S_3	H→L+1 (0.70)	431	0.003
	S_4	H–2→L (0.70)	406	0.001
	S_5	H–3→L (0.69)	384	0.071
**2**	S_1	H→L (0.70)	539	0.000
	S_2	H–1→L (0.70)	477	0.004
	S_3	H→L+1 (0.70)	431	0.004
	S_4	H–2→L (0.70)	403	0.001
	S_5	H–1→L+1 (0.70)	394	0.003
**3**	S_1	H→L (0.70)	546	0.006
	S_2	H–1→L (0.70)	496	0.003
	S_3	H→L+1 (0.68)	447	0.004
	S_4	H–1→L+1 (0.67)	414	0.000
	S_5	H–2→L (0.70)	410	0.002

[a] The orbitals involved in the excitation (H = HOMO and L = LUMO).

[b] The coefficients in the configuration interaction (CI) expansion that are less than 0.3 are not listed.

[c] Vertical excitation wavelength.

[d] Oscillator strengths.

Table S13. The intra-fragment charge redistribution (diagonal elements) and inter-fragment charge transfer (off-diagonal elements) quantities for S_1, T_1, and potential T_1' states of **1–3** computed at the corresponding optimized structures. The value in each cell records the charge transfer number from the fragment in the row to the fragment in the column. Importantly, D_{TS}→A, D_{TB}→A and A→A represent TSCT, TBCT and IL(C^C^N) characters, respectively. Overall charge transfer (%CT) and local excitation (%LE) characters are also provided

	**1**				**2**				**3**			
	D_{TS}	D_{TB}	A	Au	D_{TS}	D_{TB}	A	Au	D_{TS}	D_{TB}	A	Au
**S_1 state**												
D_{TS}	0.005	0.002	0.206	0.011	0.005	0.002	0.204	0.011	0.015	0.004	0.556	0.027
D_{TB}	0.018	0.005	0.684	0.035	0.017	0.005	0.688	0.036	0.009	0.002	0.337	0.017
A	0.000	0.000	0.014	0.001	0.000	0.000	0.013	0.001	0.001	0.000	0.020	0.001
Au	0.000	0.000	0.017	0.001	0.000	0.000	0.017	0.001	0.000	0.000	0.010	0.001
	%CT = 97.4, %LE = 2.6				%CT = 97.5, %LE = 2.5				%CT = 96.3, %LE = 3.7			
**T_1 state**												
D_{TS}	0.006	0.002	0.211	0.011	0.006	0.002	0.207	0.011	0.025	0.005	0.562	0.029
D_{TB}	0.018	0.006	0.672	0.035	0.019	0.008	0.673	0.037	0.013	0.003	0.295	0.015
A	0.000	0.000	0.018	0.001	0.000	0.000	0.016	0.001	0.002	0.000	0.037	0.002
Au	0.000	0.000	0.018	0.001	0.001	0.000	0.017	0.001	0.001	0.000	0.011	0.001
	%CT = 97.0, %LE = 3.0				%CT = 97.0, %LE = 3.0				%CT = 93.5, %LE = 6.5			
**T_1' (^{3}CT) state**												
D_{TS}	0.018	0.006	0.851	0.042	0.018	0.005	0.540	0.028	0.012	0.004	0.461	0.023
D_{TB}	0.001	0.000	0.050	0.002	0.011	0.003	0.339	0.018	0.011	0.003	0.428	0.022
A	0.001	0.000	0.025	0.001	0.001	0.000	0.025	0.001	0.000	0.000	0.019	0.001
Au	0.000	0.000	0.002	0.000	0.000	0.000	0.010	0.001	0.000	0.000	0.014	0.001
	%CT = 95.7, %LE = 4.3				%CT = 95.4, %LE = 4.6				%CT = 96.5, %LE = 3.5			
**T_1' (^{3}IL) state**												
D_{TS}	0.000	0.000	0.014	0.001	0.000	0.000	0.017	0.001	0.002	0.001	0.040	0.002
D_{TB}	0.000	0.000	0.008	0.000	0.000	0.000	0.007	0.000	0.000	0.000	0.009	0.000
A	0.025	0.013	0.878	0.033	0.021	0.011	0.885	0.030	0.032	0.012	0.841	0.033
Au	0.001	0.000	0.026	0.001	0.001	0.000	0.025	0.001	0.001	0.000	0.027	0.001
	%CT = 12.0, %LE = 88.0				%CT = 11.3, %LE = 88.7				%CT = 15.6, %LE = 84.4			

Table S14. Computed relative energy differences of the singlet and triplet excited states of **1–3** in toluene

Complex	ΔE_{S1-T1} / meV[a]	$\Delta E_{T1'(CT)-T1}$ / meV[a]	$\Delta E_{T1'(LE)-T1}$ / meV[a]
**1**	11	498	305
**2**	9	237	285
**3**	28	220	289

[a] Energy difference between the excited states at the corresponding optimized geometry.

Table S15. Computed rate constants of RISC (k_{RISC}) and RIC (k_{RIC}) processes at 298 K

Complex	$k_{RISC}(T_1 \rightarrow S_1)$ / s^{-1}	$k_{RIC}[T_1 \rightarrow T_1' (^3CT)]$ / s^{-1}	$k_{RIC}[T_1 \rightarrow T_1' (^3IL)]$ / s^{-1}
**1**	2.6×10^8	3.9×10^4	1.2×10^8
**2**	3.2×10^8	8.9×10^9	1.6×10^7
**3**	1.3×10^9	6.6×10^{10}	7.5×10^7

Table S16. Computed ΔE_{S1-T1}, ΔE_{T2-T1} and ΔE_{T3-T1} using the solid state crystal structures of **1–3**

Complex	ΔE_{S1-T1} / meV	ΔE_{T2-T1} / meV	ΔE_{T3-T1} / meV
**1**	15	264	438
**2**	14	139	403
**3**	17	243	314

OLED Fabrication and Characterization

Vacuum-deposited OLEDs were fabricated on patterned indium-tin oxide (ITO) coated glass substrates. The substrates were cleaned with Decon 90, rinsed with deionized water, dried in an oven, and finally treated in an ultraviolet-ozone chamber. Sequential thermal evaporation of a 40 nm thick *N*,*N*′-bis(naphthalene-1-yl)-*N*,*N*′-bis(phenyl)-2,2′-dimethylbenzidine (α-NPD), a 5 nm thick 4,4′,4″-tris(carbazol-9-yl)triphenylamine (TCTA), a 20 nm thick emissive layer, a 50 nm thick 1,3,5-tris(6-(3-(pyridin-3-yl)phenyl)pyridine-2-yl)benzene (Tm3PyP26PyB), a 1 nm thick LiF and a 150 nm thick aluminum was made onto the ITO substrate, in which α-NPD, TCTA, and Tm3PyP26PyB were used as hole-transporting, exciton-blocking and electron-transporting layers, respectively. The emissive layer was prepared by co-evaporating the respective boron compound and *m*-CBP as host simultaneously. All organic materials and metals were thermally evaporated by a Trovato vacuum deposition system in vacuum under a base pressure of 10^{-6} Torr. High-purity α-NPD, TCTA, Tm3PyP26PyB and *m*-CBP (> 99.5 % HPLC) were purchased from Luminescence Technology Corporation and were used as received without further purification. All films were sequentially deposited at a rate of 0.1–0.2 nm s^{-1} without vacuum break. A shadow mask was used to define the cathode and to make four 0.1 cm^2 devices on each substrate. Current density–voltage–luminance characteristics and electroluminescence (EL) spectra were measured simultaneously with a programmable Keithley model 2420 power source and a Photoresearch PR-655 spectrometer. All the devices were measured under ambient conditions without encapsulation. For operational stability testing, vacuum-deposited devices with the configuration of ITO/dipyrazino[2,3-*f*:2′,3′-*h*]quinoxaline-2,3,6,7,10,11-hexacarbonitrile (HAT-CN; 10 nm)/α-NPD (40 nm)/9,9′,9″-triphenyl-9*H*,9′*H*,9″*H*-3,3′:6′,3″-tercarbazole (Tris-PCz; 10 nm)/11 v/v% **1**–**3**:*m*-CBP (25 nm)/2,4,6-tris[3-(diphenylphosphinyl)phenyl]-1,3,5-triazine (T2T; 10 nm)/2,7-di(2,2′-bipyridin-5-yl)triphenylene (BPy-TP2; 40 nm)/LiF (1 nm)/Al (150 nm) had been fabricated and encapsulated in a glovebox under nitrogen. The initial brightness of the encapsulated device was measured by a Keithley 2400 power source

and a Photoresearch PR-655 spectrometer; while the operational lifetime of the encapsulated device was measured by a McScience OLED lifetime system by accelerated lifetime testing under a constant driving current density of 20 mA cm^{-2}.

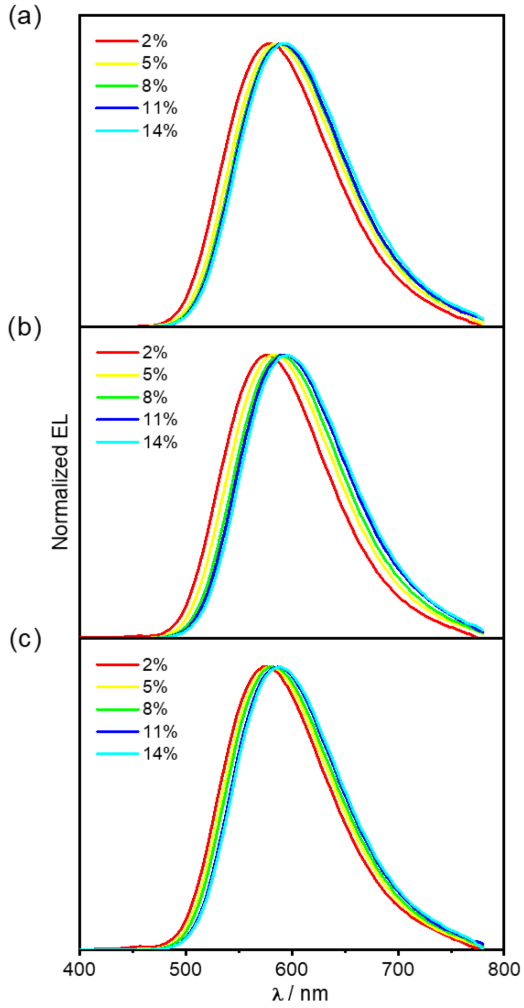

Figure S74. Normalized EL spectra of devices fabricated with (a) **1**, (b) **2** and (c) **3** doped in *m*-CBP at different concentrations.

Table S17. Key parameters of the vacuum-deposited OLEDs based on **1**–**3**

Complex	Conc. / v/v%	CE_{max}[a] / cd A^{-1}	PE_{max}[b] / lm W^{-1}	EQE_{max}[c] / %	$\Delta_{roll\text{-}off}$[d] / %	λ_{max}[e] / nm	CIE(x, y)[f]
**1**	2	21.7	17.1	8.4	27	580	0.49,0.50
	5	25.7	23.0	10.8	32	588	0.51,0.48
	8	23.6	18.8	10.2	22	588	0.52,0.47
	11	22.8	17.9	10.0	16	588	0.52,0.47
	14	21.2	14.8	9.5	10	592	0.53,0.47
**2**	2	23.6	21.2	8.5	39	576	0.48,0.50
	5	25.5	22.9	10.3	44	580	0.50,0.49
	8	24.6	22.1	10.9	27	588	0.52,0.47
	11	22.4	19.3	10.2	19	592	0.53,0.47
	14	20.8	16.9	9.9	13	586	0.54,0.46
**3**	2	17.1	13.1	6.3	13	576	0.46,0.50
	5	17.7	11.1	6.8	12	580	0.49,0.49
	8	22.6	17.8	9.0	29	580	0.50,0.49
	11	20.1	15.8	8.4	19	588	0.51,0.48
	14	18.8	13.1	7.9	13	588	0.52,0.48

[a] CE_{max} represents maximum current efficiency.

[b] PE_{max} represents maximum power efficiency.

[c] EQE_{max} represents maximum external quantum efficiency.

[d] $\Delta_{roll\text{-}off} = 1 - (CE@1000\ cd\ m^{-2} / CE_{max})$

[e] λ_{max} represents peak maximum.

[f] CIE coordinates are taken at a luminance of 100 cd m^{-2}.

Table S18. Lifetime data of devices based on 11 v/v% **1–3**

Complex	L_0[a] / cd m^{-2}	Lifetime / h at 100 cd m^{-2}		
		LT_{90}	LT_{70}	LT_{50}
**1**	2,763	982	10,836	39,873
**2**	2,732	4,030	24,065	65,314
**3**	2,104	259	5,192	24,007

[a] L_0 is defined as the initial luminance.

Table S19. Key parameters of the state-of-the-art OLEDs based on TADF/TSDP emitter with similar emission colors

Compound	λ_{max} / nm	CIE(x,y)	EQE_{max}/ %	LT_x @ L_0[a] / h
**2** (this work)	588	0.52,0.47	10.9	LT_{90}: 4,030 @ 100 cd m^{-2}<br>LT_{70}: 24,065 @ 100 cd m^{-2}<br>LT_{50}: 65,314 @ 100 cd m^{-2}
Compound **2** [Ref. 12]	592	0.55,0.45	12.3	LT_{50}: 3,594 @ 100 cd m^{-2}
Compound **8** [Ref. 13]	588	0.53,0.47	6.9	- -[b]
**Au3** [Ref. 14]	590	0.53,0.46	11.0	- -[b]
**Au-PPXZ** [Ref. 15]	586	0.51,0.48	11.3	- -[b]
**TBRb** [Ref. 16]	566	- -[b]	16.6	LT_{50}: 767 @ 100 cd m^{-2}
**BN3** [Ref. 16]	566	- -[b]	26.5	LT_{50}: 494 @ 100 cd m^{-2}
**DPPM-SXAC** [Ref. 17]	572	0.49,0.50	33.5	LT_{50}: 18,000 @ 100 cd m^{-2}

[a] LT_x (L) = LT (L_0) × (L_0/L)$^{1.7}$, where LT is lifetime, L_0 is initial luminance and L is the specific initial luminance.
[b] Not available.

Table S20. Cartesian coordinates of the optimized S_0 geometry of **1**

**1**	Au	1.090579	-0.644537	-0.603006	**48**	C	4.099046	-2.360940	-0.907501
**2**	N	3.107929	-0.101102	-0.450303	**49**	H	3.149680	-2.887630	-0.944544
**3**	N	0.498736	0.590076	-2.287631	**50**	C	-7.802663	-2.182749	-1.048392
**4**	N	-2.554960	2.818643	0.934665	**51**	C	-2.267530	4.091440	2.987193
**5**	C	5.091854	1.071420	-0.398926	**52**	H	-1.332096	4.485211	2.602909
**6**	C	0.961858	-1.999657	0.894529	**53**	C	-2.997081	3.179963	2.217497
**7**	C	-1.560475	-0.498564	-1.754477	**54**	C	3.028689	-3.724578	3.587461
**8**	C	3.673021	1.144656	-0.266076	**55**	C	-5.681773	-2.334788	0.132820
**9**	C	5.878737	2.216952	-0.310998	**56**	H	-5.162705	-2.520071	1.068209
**10**	H	6.957431	2.128859	-0.417887	**57**	C	1.284983	1.415641	-2.979660
**11**	C	-0.325306	-2.579783	1.048109	**58**	H	2.317868	1.472144	-2.650064
**12**	C	4.146091	-0.980570	-0.674044	**59**	C	-2.692457	-2.383305	-0.006476
**13**	C	-0.816594	0.444900	-2.601069	**60**	H	-3.139370	-3.128399	0.645840
**14**	C	5.396552	-0.315023	-0.637012	**61**	C	-3.517233	2.462099	-0.026566
**15**	C	-2.928277	-0.782350	-1.820600	**62**	C	-7.069579	-2.441227	0.111406
**16**	H	-3.571033	-0.228943	-2.495675	**63**	H	-7.578428	-2.717831	1.028389
**17**	C	3.068508	2.390579	-0.001177	**64**	C	-1.349011	1.156169	-3.674883
**18**	C	-0.805200	-1.144086	-0.782809	**65**	H	-2.393595	1.030961	-3.931804
**19**	C	0.970372	1.724514	1.186965	**66**	C	0.804215	2.158458	-4.048008
**20**	H	1.549625	1.009226	1.763513	**67**	H	1.469035	2.821359	-4.589313
**21**	C	2.003249	-2.361701	1.725508	**68**	C	-4.204136	2.647144	2.698559
**22**	H	2.975114	-1.901380	1.581254	**69**	C	-5.691861	-1.743975	-2.180408
**23**	C	-0.397721	1.828644	1.394895	**70**	H	-5.170771	-1.496433	-3.100190
**24**	H	-0.889544	1.183402	2.115936	**71**	C	3.574999	-2.479325	4.302697
**25**	C	3.896659	3.515173	0.056748	**72**	H	3.899009	-1.713032	3.592514
**26**	H	3.416762	4.464582	0.270856	**73**	H	4.439254	-2.747997	4.920006
**27**	C	6.587395	-1.029581	-0.804646	**74**	H	2.813305	-2.037717	4.953511
**28**	H	7.533256	-0.498684	-0.761785	**75**	C	-7.073621	-1.850316	-2.198353
**29**	C	5.291447	3.464336	-0.095892	**76**	H	-7.595782	-1.667499	-3.133628
**30**	C	1.827603	-3.330242	2.730844	**77**	C	-2.751402	4.498879	4.223674
**31**	C	0.859999	3.501576	-0.425246	**78**	H	-2.179214	5.208611	4.814008
**32**	H	1.349467	4.156443	-1.140618	**79**	C	-4.722371	1.883028	0.421536
**33**	C	-1.336337	-2.080801	0.104838	**80**	C	-4.830992	1.518646	1.895520
**34**	C	1.620392	2.537852	0.245755	**81**	C	-0.535686	2.018631	-4.397882
**35**	C	0.560012	-3.895279	2.876766	**82**	H	-0.945990	2.577620	-5.233273
**36**	H	0.383979	-4.642984	3.642416	**83**	C	7.821192	-3.250054	-1.157406
**37**	C	-0.504592	-3.524565	2.052319	**84**	C	-9.327275	-2.236583	-1.099610
**38**	H	-1.478170	-3.986402	2.197891	**85**	C	2.666703	-4.769583	4.643621
**39**	C	-1.156191	2.764500	0.685493	**86**	H	1.902525	-4.398999	5.335058
**40**	C	6.170966	4.713409	-0.012134	**87**	H	3.555250	-5.018638	5.232271
**41**	C	-3.499997	-1.723873	-0.953864	**88**	H	2.300615	-5.695823	4.188527
**42**	C	5.294653	-3.042549	-1.063641	**89**	C	-3.972352	4.019092	4.689597
**43**	H	5.252251	-4.115831	-1.229282	**90**	H	-4.362467	4.346787	5.648286
**44**	C	6.555876	-2.405372	-1.008554	**91**	C	4.120342	-4.308217	2.674908
**45**	C	-0.508026	3.629298	-0.198301	**92**	H	3.758219	-5.206927	2.164633
**46**	H	-1.071452	4.409950	-0.698740	**93**	H	5.000496	-4.582844	3.267101
**47**	C	-4.960357	-1.956988	-1.003637	**94**	H	4.438713	-3.595046	1.908591
**95**	C	-3.983453	0.250674	2.138949	**122**	H	-9.402050	-4.266709	-1.890678

**96**	H	-2.954566	0.385720	1.798878	**123**	H	-9.398286	-3.019562	-3.145753
**97**	H	-4.408582	-0.586610	1.583388	**124**	H	-10.863841	-3.309543	-2.199117
**98**	H	-3.969768	0.005371	3.206004	**125**	C	9.093010	-2.404107	-1.075818
**99**	C	-6.266186	1.219727	2.318910	**126**	H	9.971823	-3.047691	-1.186853
**100**	H	-6.300989	0.937594	3.374712	**127**	H	9.175646	-1.891080	-0.111975
**101**	H	-6.661733	0.370885	1.754020	**128**	H	9.128853	-1.651279	-1.870282
**102**	H	-6.924790	2.080521	2.167048	**129**	C	7.809133	-3.966531	-2.516153
**103**	C	-3.328306	2.702676	-1.390603	**130**	H	7.783577	-3.241152	-3.335793
**104**	H	-2.399552	3.132001	-1.742878	**131**	H	6.938577	-4.620774	-2.621212
**105**	C	-4.686417	3.099725	3.925155	**132**	H	8.707488	-4.583821	-2.632011
**106**	H	-5.625536	2.712082	4.306057	**133**	C	7.867609	-4.294713	-0.031126
**107**	C	-5.714154	1.595003	-0.512950	**134**	H	8.767290	-4.914849	-0.118731
**108**	H	-6.640395	1.135722	-0.185549	**135**	H	6.998408	-4.958694	-0.060178
**109**	C	-9.937269	-2.625341	0.248138	**136**	H	7.881041	-3.806033	0.948604
**110**	H	-9.680734	-1.905093	1.032018	**137**	C	5.354723	5.986159	0.221855
**111**	H	-9.608295	-3.618585	0.570787	**138**	H	4.635822	6.160417	-0.585786
**112**	H	-11.028229	-2.648450	0.163598	**139**	H	6.025432	6.850583	0.262292
**113**	C	-9.859932	-0.848736	-1.489271	**140**	H	4.805677	5.947745	1.168424
**114**	H	-10.954428	-0.860497	-1.535704	**141**	C	7.165585	4.561892	1.148600
**115**	H	-9.486039	-0.532251	-2.467584	**142**	H	6.635867	4.446122	2.099630
**116**	H	-9.556982	-0.095958	-0.753885	**143**	H	7.810641	5.445295	1.219694
**117**	C	-5.535092	1.851799	-1.869458	**144**	H	7.808652	3.686806	1.015611
**118**	H	-6.323542	1.610225	-2.575157	**145**	C	6.947302	4.878635	-1.327431
**119**	C	-4.334093	2.401275	-2.302586	**146**	H	7.581809	4.011300	-1.532547
**120**	H	-4.177942	2.618289	-3.356078	**147**	H	7.592359	5.763780	-1.283937
**121**	C	-9.770330	-3.268470	-2.147606	**148**	H	6.259719	4.997045	-2.171329

Table S21. Cartesian coordinates of the optimized S_1 geometry of **1**

**1**	Au	1.296118	-0.541931	-0.902180	**48**	C	3.396493	3.564594	1.132827
**2**	N	-2.870392	1.647678	0.974889	**49**	H	2.765826	4.374780	1.485840
**3**	N	3.231031	0.132285	-0.262534	**50**	C	-5.866963	3.529605	-0.045634
**4**	N	0.796578	1.023984	-2.210732	**51**	H	-6.830774	3.816558	0.360805
**5**	C	3.586301	1.315581	0.314346	**52**	C	4.782509	3.733283	1.125976
**6**	C	-1.258180	-0.198856	-2.178860	**53**	C	5.008309	1.461315	0.333855
**7**	C	-3.562466	0.609188	1.659762	**54**	C	-3.897549	-1.708393	2.221508
**8**	C	-4.781158	0.951778	2.268875	**55**	H	-3.561577	-2.739345	2.168021
**9**	C	4.395575	-0.537101	-0.571401	**56**	C	2.700291	-5.774151	3.365022
**10**	C	5.534331	0.222643	-0.208991	**57**	H	1.831334	-5.646363	4.018971
**11**	C	-0.530083	0.934548	-2.680951	**58**	H	3.537756	-6.108496	3.985821
**12**	C	2.136173	-2.754137	1.054363	**59**	H	2.477664	-6.572067	2.649055
**13**	H	3.076415	-2.222101	1.161882	**60**	C	-0.280729	-4.095795	0.792355
**14**	C	-0.224394	2.983197	-3.941049	**61**	H	-1.224650	-4.628971	0.704145
**15**	H	-0.606402	3.735418	-4.621658	**62**	C	-5.533895	-0.061089	2.858627
**16**	C	-3.730578	2.461999	0.190237	**63**	H	-6.484878	0.175963	3.322779
**17**	C	-2.639053	-0.466927	-2.283525	**64**	C	-1.500983	1.857767	1.034953
**18**	H	-3.288274	0.224411	-2.813737	**65**	C	5.821513	-2.280785	-1.353259
**19**	C	-3.137755	-0.716077	1.613615	**66**	H	5.937959	-3.263327	-1.799392
**20**	C	3.072172	-4.469680	2.658580	**67**	C	1.165813	-2.245785	0.208421
**21**	C	5.593337	2.643766	0.732728	**68**	C	0.744114	1.121495	1.586130
**22**	H	6.673086	2.741390	0.719709	**69**	H	1.391337	0.381541	2.047510
**23**	C	-1.011084	1.916124	-3.553282	**70**	C	3.405459	-3.418140	3.728110
**24**	H	-2.025689	1.817295	-3.930839	**71**	H	3.723546	-2.472287	3.279435
**25**	C	1.111286	3.055118	-3.444081	**72**	H	4.218651	-3.773877	4.370790
**26**	H	1.776430	3.863877	-3.723554	**73**	H	2.533808	-3.215770	4.358952
**27**	C	-0.517272	-1.101877	-1.396197	**74**	C	-2.404070	-2.506046	-0.963403
**28**	C	0.433987	3.212934	0.454912	**75**	H	-2.876490	-3.332098	-0.440524
**29**	H	0.836111	4.103508	-0.018579	**76**	C	-6.710198	-2.966943	-1.194784
**30**	C	-3.210577	-1.573408	-1.652486	**77**	C	5.375910	5.069110	1.567483
**31**	C	0.720538	-4.605669	1.624121	**78**	C	-1.043446	-2.245162	-0.799647
**32**	H	0.529200	-5.529429	2.159521	**79**	C	4.536934	-1.795392	-1.164301
**33**	C	-0.075589	-2.923671	0.075222	**80**	H	3.665030	-2.375843	-1.447756
**34**	C	1.941786	-3.949752	1.771808	**81**	C	6.972198	-1.552558	-0.985532
**35**	C	1.312129	2.229496	0.935994	**82**	C	-5.348813	-2.898550	-1.480492
**36**	C	1.561142	2.066682	-2.609331	**83**	H	-4.796751	-3.815498	-1.670009
**37**	H	2.573466	2.080426	-2.218696	**84**	C	-5.437400	-0.514272	-1.301466
**38**	C	-0.625276	0.957322	1.676389	**85**	H	-4.946364	0.453823	-1.297551
**39**	C	-4.679934	-1.673256	-1.523304	**86**	C	-6.791653	-0.588172	-1.018254
**40**	C	-4.332594	3.484459	-1.904822	**87**	H	-7.327558	0.337103	-0.822480
**41**	H	-4.088306	3.721442	-2.936003	**88**	C	-5.093423	-1.382753	2.849542
**42**	C	6.810618	-0.279704	-0.418789	**89**	H	-5.698752	-2.156071	3.312697
**43**	H	7.675497	0.310298	-0.136185	**90**	C	8.347051	-2.177962	-1.206865
**44**	C	-4.960395	2.832315	0.752282	**91**	C	4.817216	6.188361	0.674724
**45**	C	-5.557055	3.863799	-1.361716	**92**	H	5.080909	6.019341	-0.374035
**46**	H	-6.277286	4.409710	-1.963384	**93**	H	5.232434	7.154334	0.981079
**47**	C	-3.422455	2.771028	-1.134824	**94**	H	3.727693	6.256893	0.741574
**95**	C	-7.459914	-1.816521	-0.937937	**122**	H	-8.490710	-1.823823	1.593002

**96**	C	-9.757806	-1.019983	-1.535924	**123**	H	-10.127003	-1.264150	1.184041
**97**	H	-9.424119	0.021360	-1.567494	**124**	H	-8.727182	-0.215505	0.900371
**98**	H	-10.816062	-1.024509	-1.251441	**125**	C	8.436521	-3.484989	-0.402213
**99**	H	-9.673468	-1.429916	-2.547406	**126**	H	8.303823	-3.293282	0.667380
**100**	C	4.316145	-4.725140	1.791806	**127**	H	9.417218	-3.950502	-0.548341
**101**	H	4.104806	-5.476752	1.024119	**128**	H	7.673997	-4.205795	-0.711031
**102**	H	5.142480	-5.091837	2.411419	**129**	C	2.755732	2.365729	0.785551
**103**	H	4.653653	-3.815815	1.285212	**130**	C	-0.932663	3.032457	0.490116
**104**	C	4.986177	5.336709	3.030170	**131**	H	-7.183355	-3.942796	-1.164315
**105**	H	3.901102	5.384056	3.158928	**132**	H	-2.257378	-0.983041	1.044789
**106**	H	5.406085	6.292582	3.361102	**133**	H	-1.573372	3.798897	0.074189
**107**	H	5.369374	4.548908	3.686278	**134**	H	-1.008820	0.106375	2.220314
**108**	C	-8.934056	-1.850638	-0.541165	**135**	H	-2.485684	2.429879	-1.561016
**109**	C	6.902350	5.088291	1.464352	**136**	C	8.534773	-2.481388	-2.701648
**110**	H	7.364391	4.337478	2.113713	**137**	H	7.776226	-3.174957	-3.075299
**111**	H	7.275200	6.068097	1.777389	**138**	H	9.516639	-2.935982	-2.872058
**112**	H	7.242835	4.917068	0.437832	**139**	H	8.474132	-1.564156	-3.295855
**113**	C	-9.496484	-3.272736	-0.519249	**140**	C	-5.224109	2.408274	2.191773
**114**	H	-9.430659	-3.748364	-1.503327	**141**	C	-6.686644	2.588223	2.585651
**115**	H	-10.552572	-3.246698	-0.231990	**142**	H	-6.842612	2.284321	3.624269
**116**	H	-8.970981	-3.904808	0.204055	**143**	H	-6.974138	3.640922	2.515648
**117**	C	9.483308	-1.257842	-0.756993	**144**	H	-7.355834	2.001259	1.948769
**118**	H	9.487685	-0.315377	-1.314485	**145**	C	-4.357156	3.262894	3.141722
**119**	H	10.443749	-1.751218	-0.934428	**146**	H	-3.293676	3.180348	2.907171
**120**	H	9.421659	-1.028706	0.311983	**147**	H	-4.644835	4.316066	3.063807
**121**	C	-9.077855	-1.251673	0.867428	**148**	H	-4.505745	2.936139	4.175557

Table S22. Cartesian coordinates of the optimized T_1 geometry of **1**

**1**	Au	1.299121	-0.542352	-0.901236	**48**	C	3.389047	3.568857	1.126383
**2**	N	-2.875018	1.647252	0.974334	**49**	H	2.756724	4.378963	1.476663
**3**	N	3.230609	0.132610	-0.261537	**50**	C	-5.873930	3.531282	-0.035321
**4**	N	0.797693	1.029274	-2.203394	**51**	H	-6.837173	3.816636	0.373559
**5**	C	3.583176	1.317778	0.313524	**52**	C	4.774694	3.739366	1.121856
**6**	C	-1.256173	-0.194318	-2.174897	**53**	C	5.004807	1.465683	0.335191
**7**	C	-3.565513	0.606415	1.657538	**54**	C	-3.897702	-1.712766	2.213987
**8**	C	-4.783225	0.946689	2.269778	**55**	H	-3.561114	-2.743323	2.157071
**9**	C	4.397258	-0.535610	-0.566981	**56**	C	2.704377	-5.791346	3.345065
**10**	C	5.533775	0.226932	-0.204120	**57**	H	1.834369	-5.667984	3.998470
**11**	C	-0.528692	0.940984	-2.673484	**58**	H	3.541521	-6.127385	3.965379
**12**	C	2.139073	-2.761542	1.047570	**59**	H	2.483899	-6.586181	2.625014
**13**	H	3.078556	-2.228901	1.158529	**60**	C	-0.276084	-4.104768	0.777397
**14**	C	-0.222430	2.990760	-3.931453	**61**	H	-1.219371	-4.638529	0.685981
**15**	H	-0.604099	3.743797	-4.611360	**62**	C	-5.533985	-0.068256	2.858520
**16**	C	-3.736895	2.463683	0.193619	**63**	H	-6.484230	0.166906	3.325127
**17**	C	-2.637074	-0.461810	-2.279579	**64**	C	-1.505979	1.857547	1.032941
**18**	H	-3.286788	0.232406	-2.805387	**65**	C	5.828145	-2.277414	-1.343862
**19**	C	-3.139940	-0.718376	1.607011	**66**	H	5.947254	-3.260390	-1.788401
**20**	C	3.075283	-4.482980	2.645342	**67**	C	1.168946	-2.250620	0.202834
**21**	C	5.587555	2.649959	0.732651	**68**	C	0.739898	1.120478	1.579333
**22**	H	6.667224	2.748804	0.721506	**69**	H	1.387959	0.380624	2.039642
**23**	C	-1.009624	1.923724	-3.544558	**70**	C	3.405376	-3.435994	3.720314
**24**	H	-2.024222	1.825502	-3.922264	**71**	H	3.722637	-2.487547	3.276570
**25**	C	1.113513	3.060846	-3.435592	**72**	H	4.218205	-3.793516	4.362462
**26**	H	1.779154	3.869296	-3.714858	**73**	H	2.532505	-3.237983	4.350857
**27**	C	-0.513537	-1.101502	-1.398345	**74**	C	-2.400318	-2.507638	-0.970208
**28**	C	0.427839	3.214694	0.453237	**75**	H	-2.872133	-3.336452	-0.451150
**29**	H	0.829104	4.106390	-0.018814	**76**	C	-6.706699	-2.968248	-1.199744
**30**	C	-3.207861	-1.571773	-1.654291	**77**	C	5.365526	5.076942	1.561812
**31**	C	0.725013	-4.617325	1.607608	**78**	C	-1.039825	-2.247444	-0.806229
**32**	H	0.534269	-5.543705	2.138670	**79**	C	4.542269	-1.794338	-1.157862
**33**	C	-0.071685	-2.929141	0.065752	**80**	H	3.672083	-2.376828	-1.442459
**34**	C	1.945352	-3.960571	1.759425	**81**	C	6.976872	-1.546337	-0.975368
**35**	C	1.307238	2.230377	0.930951	**82**	C	-5.345429	-2.898390	-1.485608
**36**	C	1.563344	2.071397	-2.602101	**83**	H	-4.793117	-3.814543	-1.678177
**37**	H	2.575941	2.083865	-2.212221	**84**	C	-5.435079	-0.514743	-1.299161
**38**	C	-0.629198	0.956065	1.671554	**85**	H	-4.944599	0.453600	-1.292289
**39**	C	-4.677060	-1.672659	-1.524825	**86**	C	-6.789191	-0.590088	-1.015823
**40**	C	-4.342575	3.492620	-1.897173	**87**	H	-7.325491	0.334337	-0.817147
**41**	H	-4.100152	3.733063	-2.927980	**88**	C	-5.092469	-1.389523	2.845422
**42**	C	6.811720	-0.273209	-0.411144	**89**	H	-5.696242	-2.164414	3.307965
**43**	H	7.674815	0.319289	-0.128250	**90**	C	8.353330	-2.169479	-1.193541
**44**	C	-4.965949	2.831616	0.758879	**91**	C	4.806682	6.193868	0.666287
**45**	C	-5.566238	3.869796	-1.350812	**92**	H	5.072110	6.023297	-0.381787
**46**	H	-6.287607	4.417428	-1.949539	**93**	H	5.220092	7.161007	0.971454
**47**	C	-3.430896	2.777145	-1.130894	**94**	H	3.716957	6.261033	0.731405
**95**	C	-7.456911	-1.818962	-0.939221	**122**	H	-8.488420	-1.829278	1.591348

**96**	C	-9.755107	-1.023881	-1.537301	**123**	H	-10.125373	-1.271549	1.182387
**97**	H	-9.422170	0.017743	-1.567692	**124**	H	-8.726985	-0.220675	0.900159
**98**	H	-10.813496	-1.029514	-1.253326	**125**	C	8.444019	-3.475276	-0.387071
**99**	H	-9.669959	-1.432771	-2.549136	**126**	H	8.309150	-3.282409	0.682046
**100**	C	4.320950	-4.732399	1.779248	**127**	H	9.425821	-3.939203	-0.530923
**101**	H	4.111829	-5.480342	1.007376	**128**	H	7.683333	-4.197914	-0.696180
**102**	H	5.146733	-5.101229	2.398332	**129**	C	2.750378	2.368029	0.780948
**103**	H	4.658161	-3.820060	1.277922	**130**	C	-0.938492	3.033862	0.489794
**104**	C	4.973389	5.346664	3.023440	**131**	H	-7.179419	-3.944401	-1.172215
**105**	H	3.888046	5.392597	3.150549	**132**	H	-2.260572	-0.983248	1.035618
**106**	H	5.391389	6.303736	3.353366	**133**	H	-1.579978	3.801072	0.076534
**107**	H	5.356768	4.560554	3.681478	**134**	H	-1.011813	0.104181	2.214638
**108**	C	-8.931127	-1.854818	-0.542945	**135**	H	-2.494544	2.437878	-1.559503
**109**	C	6.892085	5.098050	1.460831	**136**	C	8.544357	-2.474492	-2.687560
**110**	H	7.354219	4.349155	2.112338	**137**	H	7.787689	-3.169882	-3.061661
**111**	H	7.263183	6.078996	1.772422	**138**	H	9.527342	-2.927538	-2.855738
**112**	H	7.234245	4.925163	0.435146	**139**	H	8.483066	-1.558122	-3.283036
**113**	C	-9.492249	-3.277448	-0.522666	**140**	C	-5.227291	2.403074	2.197452
**114**	H	-9.426014	-3.751903	-1.507281	**141**	C	-6.689394	2.580850	2.593932
**115**	H	-10.548365	-3.252710	-0.235400	**142**	H	-6.843675	2.273707	3.631841
**116**	H	-8.966140	-3.909852	0.199911	**143**	H	-6.977690	3.633562	2.527586
**117**	C	9.487008	-1.246591	-0.742854	**144**	H	-7.359084	1.995371	1.956212
**118**	H	9.490510	-0.304841	-1.301562	**145**	C	-4.359534	3.255491	3.148679
**119**	H	10.448768	-1.738294	-0.917866	**146**	H	-3.296311	3.174243	2.912487
**120**	H	9.422886	-1.016122	0.325692	**147**	H	-4.647909	4.308715	3.074189
**121**	C	-9.076146	-1.257314	0.866124	**148**	H	-4.506521	2.925663	4.181760

Table S23. Cartesian coordinates of the optimized T_1' (^{3}CT) geometry of **1**

**1**	Au	1.168751	-0.851608	-0.665681	**48**	C	4.302475	-2.362423	-0.580405
**2**	N	3.151080	-0.145996	-0.399935	**49**	H	3.389852	-2.950947	-0.552113
**3**	N	0.696630	0.076259	-2.495378	**50**	C	-7.707016	-2.095374	-1.063947
**4**	N	-2.665690	2.570438	0.900836	**51**	C	-2.104239	3.963236	2.791534
**5**	C	5.045574	1.167765	-0.474543	**52**	H	-1.117002	4.114315	2.374293
**6**	C	0.983712	-1.918950	1.050954	**53**	C	-3.029109	3.145224	2.112602
**7**	C	-1.380509	-1.003399	-2.002828	**54**	C	2.898269	-3.103395	4.131364
**8**	C	3.621944	1.144508	-0.351557	**55**	C	-5.592778	-2.167039	0.137102
**9**	C	5.741625	2.371439	-0.509897	**56**	H	-5.070534	-2.162612	1.088393
**10**	H	6.824553	2.355995	-0.609514	**57**	C	1.474449	0.887928	-3.242029
**11**	C	-0.311663	-2.469732	1.252926	**58**	H	2.481086	1.040138	-2.864019
**12**	C	4.251947	-0.963508	-0.516993	**59**	C	-2.634148	-2.466392	0.099654
**13**	C	-0.628864	-0.175677	-2.904051	**60**	H	-3.123480	-3.033702	0.885471
**14**	C	5.453166	-0.210949	-0.557077	**61**	C	-3.614454	2.089196	0.018057
**15**	C	-2.748365	-1.338756	-2.061500	**62**	C	-6.984200	-2.136010	0.130408
**16**	H	-3.350176	-0.983721	-2.890785	**63**	H	-7.504613	-2.129854	1.082760
**17**	C	2.926215	2.363539	-0.209628	**64**	C	-1.091968	0.407407	-4.092110
**18**	C	-0.688798	-1.452901	-0.864868	**65**	H	-2.102418	0.179886	-4.421181
**19**	C	0.898783	1.600185	1.030643	**66**	C	1.042404	1.491916	-4.395742
**20**	H	1.526460	0.918082	1.594028	**67**	H	1.716593	2.131673	-4.952807
**21**	C	1.974552	-2.120880	1.994719	**68**	C	-4.328672	2.937131	2.627235
**22**	H	2.956731	-1.692152	1.818519	**69**	C	-5.577413	-2.170979	-2.247517
**23**	C	-0.465064	1.635894	1.282064	**70**	H	-5.047285	-2.218661	-3.194253
**24**	H	-0.908790	0.967893	2.013311	**71**	C	3.413897	-1.744923	4.630744
**25**	C	3.665515	3.548568	-0.270847	**72**	H	3.769540	-1.120245	3.806208
**26**	H	3.117719	4.477248	-0.148172	**73**	H	4.248655	-1.887540	5.326290
**27**	C	6.691185	-0.852931	-0.647151	**74**	H	2.622692	-1.197152	5.153615
**28**	H	7.597748	-0.256274	-0.672530	**75**	C	-6.964708	-2.136120	-2.252039
**29**	C	5.059862	3.587885	-0.425713	**76**	H	-7.480034	-2.143726	-3.209235
**30**	C	1.743425	-2.881281	3.156616	**77**	C	-2.472300	4.588013	3.966569
**31**	C	0.650787	3.329365	-0.626424	**78**	H	-1.761824	5.226075	4.480393
**32**	H	1.080573	3.967617	-1.391526	**79**	C	-4.941760	1.870544	0.474600
**33**	C	-1.270909	-2.174264	0.175312	**80**	C	-5.236776	1.926725	1.959755
**34**	C	1.477722	2.428469	0.057481	**81**	C	-0.288312	1.238802	-4.843638
**35**	C	0.466927	-3.410313	3.345065	**82**	H	-0.656641	1.680114	-5.763248
**36**	H	0.246963	-4.001202	4.228040	**83**	C	8.078647	-3.006600	-0.765794
**37**	C	-0.551054	-3.204500	2.408304	**84**	C	-9.230236	-2.004642	-1.115076
**38**	H	-1.533616	-3.636640	2.585210	**85**	C	2.481761	-3.936525	5.344292
**39**	C	-1.262928	2.530913	0.576752	**86**	H	1.685165	-3.448489	5.916071
**40**	C	5.841762	4.901157	-0.482936	**87**	H	3.338908	-4.068434	6.012564
**41**	C	-3.376959	-2.024514	-1.020733	**88**	H	2.133353	-4.932051	5.050194
**42**	C	5.543927	-2.970901	-0.661039	**89**	C	-3.757231	4.403487	4.477438
**43**	H	5.579256	-4.056463	-0.699498	**90**	H	-4.054855	4.895926	5.397182
**44**	C	6.756376	-2.243314	-0.693095	**91**	C	4.033409	-3.840149	3.401147
**45**	C	-0.711335	3.401040	-0.359441	**92**	H	3.692963	-4.818976	3.047546
**46**	H	-1.342726	4.114086	-0.880425	**93**	H	4.882124	-3.996297	4.076942
**47**	C	-4.850371	-2.153491	-1.047822	**94**	H	4.391742	-3.278422	2.533158
**95**	C	-4.868799	0.536874	2.545973	**122**	H	-9.522481	-4.152238	-1.340261

**96**	H	-3.832200	0.267238	2.324489	**123**	H	-9.412247	-3.289098	-2.879998
**97**	H	-5.518720	-0.221333	2.104079	**124**	H	-10.886517	-3.169390	-1.908843
**98**	H	-5.005648	0.544486	3.631069	**125**	C	9.286763	-2.068680	-0.795309
**99**	C	-6.714923	2.196787	2.245472	**126**	H	10.208887	-2.656801	-0.848034
**100**	H	-6.911884	2.172448	3.318943	**127**	H	9.338582	-1.448140	0.105482
**101**	H	-7.339053	1.415687	1.806975	**128**	H	9.263105	-1.407163	-1.667641
**102**	H	-7.032406	3.165561	1.850441	**129**	C	8.108843	-3.865814	-2.039072
**103**	C	-3.268053	1.862676	-1.330452	**130**	H	8.023476	-3.237672	-2.931626
**104**	H	-2.241783	1.964924	-1.655926	**131**	H	7.287310	-4.587885	-2.059289
**105**	C	-4.664593	3.582712	3.811355	**132**	H	9.049192	-4.425815	-2.101264
**106**	H	-5.651106	3.443326	4.236257	**133**	C	8.209380	-3.916704	0.465591
**107**	C	-5.885914	1.469827	-0.457169	**134**	H	9.150053	-4.478535	0.429791
**108**	H	-6.900024	1.258877	-0.142982	**135**	H	7.389398	-4.638717	0.522746
**109**	C	-9.853146	-1.958220	0.281195	**136**	H	8.197569	-3.324748	1.386626
**110**	H	-9.505067	-1.089929	0.851402	**137**	C	4.932144	6.126538	-0.373884
**111**	H	-9.625231	-2.861031	0.856952	**138**	H	4.198774	6.159432	-1.186586
**112**	H	-10.941872	-1.884446	0.196203	**139**	H	5.535254	7.038426	-0.432955
**113**	C	-9.631553	-0.724052	-1.863791	**140**	H	4.392076	6.147256	0.578615
**114**	H	-10.722490	-0.640146	-1.920887	**141**	C	6.850818	4.949167	0.674367
**115**	H	-9.241126	-0.714229	-2.885776	**142**	H	6.336591	4.895213	1.639418
**116**	H	-9.249318	0.164241	-1.348480	**143**	H	7.426905	5.881264	0.643346
**117**	C	-5.558741	1.309357	-1.804132	**144**	H	7.558465	4.116260	0.626088
**118**	H	-6.325345	0.998323	-2.504786	**145**	C	6.596804	4.983996	-1.818404
**119**	C	-4.248312	1.508701	-2.234123	**146**	H	7.293559	4.149262	-1.939067
**120**	H	-3.982024	1.368299	-3.274912	**147**	H	7.173357	5.914493	-1.876690
**121**	C	-9.793572	-3.226117	-1.856662	**148**	H	5.897900	4.959643	-2.660743

Table S24. Cartesian coordinates of the optimized T_1' (^{3}IL) geometry of **1**

**1**	Au	1.117198	-0.657804	-0.621105	**48**	C	4.150143	-2.344160	-0.910433
**2**	N	3.128162	-0.100678	-0.438949	**49**	H	3.207587	-2.882504	-0.952506
**3**	N	0.538317	0.588645	-2.301437	**50**	C	-7.809141	-2.148537	-1.076485
**4**	N	-2.571935	2.762561	0.961442	**51**	C	-2.288346	4.015373	3.027628
**5**	C	5.096421	1.097140	-0.375004	**52**	H	-1.345951	4.403405	2.654867
**6**	C	0.980553	-2.008404	0.866776	**53**	C	-3.020513	3.118091	2.243759
**7**	C	-1.518375	-0.499830	-1.771165	**54**	C	3.002023	-3.771953	3.566002
**8**	C	3.676370	1.151098	-0.244388	**55**	C	-5.694574	-2.392780	0.101739
**9**	C	5.869101	2.251412	-0.276882	**56**	H	-5.180581	-2.632150	1.027612
**10**	H	6.948993	2.177291	-0.382396	**57**	C	1.317014	1.422877	-2.986064
**11**	C	-0.361739	-2.578805	1.019521	**58**	H	2.351127	1.480277	-2.660164
**12**	C	4.178133	-0.964936	-0.666883	**59**	C	-2.719123	-2.418857	-0.021326
**13**	C	-0.782106	0.442519	-2.611011	**60**	H	-3.160709	-3.178549	0.615904
**14**	C	5.419635	-0.283200	-0.623234	**61**	C	-3.530627	2.429826	-0.011328
**15**	C	-2.916071	-0.774048	-1.832914	**62**	C	-7.081392	-2.482923	0.069579
**16**	H	-3.564508	-0.172743	-2.457021	**63**	H	-7.596973	-2.804428	0.967958
**17**	C	3.055881	2.387526	0.029166	**64**	C	-1.320573	1.164971	-3.677216
**18**	C	-0.771852	-1.148056	-0.814761	**65**	H	-2.365656	1.038978	-3.931971
**19**	C	0.960722	1.692530	1.208512	**66**	C	0.829218	2.178787	-4.045702
**20**	H	1.544166	0.974443	1.776964	**67**	H	1.490024	2.851022	-4.580183
**21**	C	1.999477	-2.382153	1.699943	**68**	C	-4.237237	2.593977	2.710295
**22**	H	2.980607	-1.940042	1.560907	**69**	C	-5.690639	-1.660385	-2.173781
**23**	C	-0.408313	1.785191	1.416231	**70**	H	-5.167293	-1.356804	-3.074725
**24**	H	-0.897630	1.128358	2.128675	**71**	C	3.583761	-2.537091	4.274248
**25**	C	3.870483	3.521559	0.097221	**72**	H	3.914871	-1.777287	3.560470
**26**	H	3.378589	4.463248	0.318048	**73**	H	4.450358	-2.825033	4.879501
**27**	C	6.620047	-0.980047	-0.796753	**74**	H	2.840423	-2.080433	4.935692
**28**	H	7.558510	-0.436502	-0.749855	**75**	C	-7.072684	-1.755204	-2.202345
**29**	C	5.265996	3.489639	-0.053351	**76**	H	-7.590106	-1.508013	-3.125358
**30**	C	1.807315	-3.353637	2.721309	**77**	C	-2.778316	4.416642	4.263776
**31**	C	0.839563	3.484312	-0.386385	**78**	H	-2.203791	5.115388	4.864864
**32**	H	1.324918	4.149053	-1.095404	**79**	C	-4.747884	1.864011	0.422175
**33**	C	-1.316522	-2.112019	0.109275	**80**	C	-4.870608	1.481430	1.890497
**34**	C	1.605997	2.518993	0.275561	**81**	C	-0.511314	2.038011	-4.392681
**35**	C	0.504454	-3.900853	2.879573	**82**	H	-0.925993	2.605645	-5.220129
**36**	H	0.328422	-4.633613	3.659747	**83**	C	7.884212	-3.179481	-1.172765
**37**	C	-0.548775	-3.533442	2.074626	**84**	C	-9.333891	-2.177319	-1.132870
**38**	H	-1.530571	-3.971167	2.231519	**85**	C	2.628519	-4.807628	4.627908
**39**	C	-1.172208	2.722257	0.714947	**86**	H	1.880101	-4.420011	5.327191
**40**	C	6.129515	4.749019	0.041475	**87**	H	3.517693	-5.074235	5.207716
**41**	C	-3.505177	-1.749898	-0.945713	**88**	H	2.238684	-5.727015	4.178711
**42**	C	5.354986	-3.008178	-1.072371	**89**	C	-4.007619	3.945108	4.715786
**43**	H	5.327102	-4.080439	-1.247675	**90**	H	-4.402061	4.267989	5.674341
**44**	C	6.607424	-2.354289	-1.012858	**91**	C	4.074992	-4.379864	2.644626
**45**	C	-0.529121	3.601108	-0.158860	**92**	H	3.691401	-5.273118	2.140639
**46**	H	-1.097108	4.383220	-0.651732	**93**	H	4.954254	-4.669317	3.231119
**47**	C	-4.961425	-1.957131	-1.010699	**94**	H	4.401305	-3.674717	1.874217
**95**	C	-4.039376	0.201110	2.125798	**122**	H	-9.435838	-4.152334	-2.050594

**96**	H	-3.005327	0.329651	1.798298	**123**	H	-9.411084	-2.828590	-3.224478
**97**	H	-4.468392	-0.625541	1.556924	**124**	H	-10.882906	-3.156456	-2.300263
**98**	H	-4.038630	-0.056100	3.190170	**125**	C	9.144288	-2.316980	-1.082566
**99**	C	-6.311919	1.192983	2.299988	**126**	H	10.031813	-2.947069	-1.201476
**100**	H	-6.357442	0.899331	3.352251	**127**	H	9.220646	-1.813663	-0.113095
**101**	H	-6.711605	0.354466	1.722586	**128**	H	9.169109	-1.554884	-1.868560
**102**	H	-6.960586	2.062176	2.153181	**129**	C	7.880271	-3.879643	-2.540078
**103**	C	-3.328331	2.679916	-1.372012	**130**	H	7.843876	-3.144809	-3.350841
**104**	H	-2.388539	3.091854	-1.715310	**131**	H	7.018443	-4.544218	-2.652099
**105**	C	-4.724777	3.040246	3.937034	**132**	H	8.786774	-4.483254	-2.664345
**106**	H	-5.671089	2.658930	4.306369	**133**	C	7.946921	-4.236911	-0.059368
**107**	C	-5.740165	1.608573	-0.521004	**134**	H	8.854994	-4.843345	-0.155826
**108**	H	-6.676191	1.161515	-0.204624	**135**	H	7.087077	-4.912634	-0.095327
**109**	C	-9.952600	-2.641226	0.186819	**136**	H	7.955203	-3.760030	0.926213
**110**	H	-9.689628	-1.974134	1.014375	**137**	C	5.296836	6.009635	0.283135
**111**	H	-9.636716	-3.656551	0.447967	**138**	H	4.577295	6.180681	-0.524616
**112**	H	-11.043493	-2.645343	0.098549	**139**	H	5.956505	6.882132	0.331221
**113**	C	-9.846040	-0.760129	-1.435335	**140**	H	4.746517	5.957347	1.228304
**114**	H	-10.940514	-0.753420	-1.484104	**141**	C	7.123694	4.601466	1.203100
**115**	H	-9.465180	-0.388014	-2.391145	**142**	H	6.593587	4.472328	2.152199
**116**	H	-9.534494	-0.059567	-0.653372	**143**	H	7.757636	5.492241	1.281873
**117**	C	-5.550186	1.882378	-1.872817	**144**	H	7.777772	3.735355	1.065099
**118**	H	-6.340090	1.667145	-2.585375	**145**	C	6.906279	4.933681	-1.270979
**119**	C	-4.335535	2.411176	-2.292812	**146**	H	7.552065	4.075923	-1.481100
**120**	H	-4.167718	2.635858	-3.342884	**147**	H	7.540025	5.826556	-1.219740
**121**	C	-9.789054	-3.134636	-2.244611	**148**	H	6.218889	5.049537	-2.115382

Table S25. Cartesian coordinates of the optimized S_0 geometry of **2**

**1**	Au	1.007311	-0.551684	-0.733794	**48**	C	4.093241	-2.076852	-1.327884
**2**	N	3.003769	0.025168	-0.504404	**49**	H	3.164800	-2.616396	-1.494807
**3**	N	0.381195	0.883327	-2.231810	**50**	C	-7.930206	-1.692733	-0.796899
**4**	N	-2.818614	2.486638	1.215446	**51**	C	-2.633044	2.625690	3.658494
**5**	C	4.935308	1.226610	-0.141169	**52**	H	-1.683349	3.140004	3.556211
**6**	C	0.913361	-2.063532	0.606247	**53**	C	-3.316046	2.233234	2.506452
**7**	C	-1.678384	-0.232782	-1.759529	**54**	C	3.052491	-4.132131	2.980327
**8**	C	3.510756	1.226919	-0.053057	**55**	C	-5.771996	-1.728455	0.324788
**9**	C	5.673442	2.356849	0.200685	**56**	H	-5.210761	-1.751831	1.254038
**10**	H	6.757561	2.327090	0.119480	**57**	C	1.154840	1.791246	-2.827929
**11**	C	-0.389010	-2.600040	0.785531	**58**	H	2.195786	1.799654	-2.519226
**12**	C	4.081019	-0.762348	-0.844825	**59**	C	-2.806757	-2.171982	-0.065091
**13**	C	-0.944093	0.787367	-2.521517	**60**	H	-3.269003	-2.915663	0.578861
**14**	C	5.302453	-0.076531	-0.631039	**61**	C	-3.736684	2.427248	0.149199
**15**	C	-3.061440	-0.445160	-1.764464	**62**	C	-7.161852	-1.789051	0.365766
**16**	H	-3.721540	0.176131	-2.362058	**63**	H	-7.645077	-1.891956	1.331113
**17**	C	2.848359	2.373895	0.431991	**64**	C	-1.499246	1.632934	-3.480341
**18**	C	-0.901672	-1.004036	-0.901688	**65**	H	-2.552639	1.550398	-3.717881
**19**	C	0.729770	1.429616	1.383174	**66**	C	0.650862	2.669662	-3.775915
**20**	H	1.310602	0.630647	1.834549	**67**	H	1.304509	3.397722	-4.241867
**21**	C	1.984527	-2.551650	1.327372	**68**	C	-4.547199	1.586245	2.653047
**22**	H	2.966992	-2.119700	1.168614	**69**	C	-5.856747	-1.491806	-2.056198
**23**	C	-0.648926	1.444963	1.542253	**70**	H	-5.354741	-1.389176	-3.014349
**24**	H	-1.142664	0.639696	2.077064	**71**	C	3.693556	-2.987331	3.780108
**25**	C	3.631089	3.489393	0.744307	**72**	H	4.008673	-2.165565	3.130441
**26**	H	3.108564	4.360661	1.125511	**73**	H	4.579640	-3.349746	4.312777
**27**	C	6.524198	-0.708941	-0.884100	**74**	H	2.990974	-2.585800	4.517626
**28**	H	7.447559	-0.166881	-0.705474	**75**	C	-7.241001	-1.553418	-2.010218
**29**	C	5.030774	3.516031	0.636710	**76**	H	-7.796775	-1.491488	-2.941994
**30**	C	1.822324	-3.606546	2.243987	**77**	C	-3.147504	2.339048	4.920237
**31**	C	0.618042	3.488694	0.157427	**78**	H	-2.590536	2.639715	5.801939
**32**	H	1.107513	4.287793	-0.392175	**79**	C	-4.966239	1.779797	0.361115
**33**	C	-1.427078	-1.973048	-0.045459	**80**	C	-0.698185	2.578258	-4.107230
**34**	C	1.387891	2.429053	0.648041	**81**	H	-1.125982	3.242084	-4.852311
**35**	C	0.539101	-4.126360	2.418935	**82**	C	7.852817	-2.781007	-1.603634
**36**	H	0.372940	-4.937468	3.119561	**83**	C	-9.457194	-1.721661	-0.788945
**37**	C	-0.554188	-3.629842	1.705344	**84**	C	2.706332	-5.264858	3.947599
**38**	H	-1.538606	-4.061794	1.868559	**85**	H	2.002150	-4.937348	4.719759
**39**	C	-1.411749	2.495189	1.021117	**86**	H	3.615697	-5.607972	4.451019
**40**	C	5.856810	4.752003	0.998569	**87**	H	2.271704	-6.124078	3.426140
**41**	C	-3.625274	-1.407507	-0.916740	**88**	C	-4.365389	1.679581	5.048921
**42**	C	5.318477	-2.679303	-1.561653	**89**	H	-4.770292	1.453161	6.029905
**43**	H	5.323345	-3.704106	-1.923473	**90**	C	4.063515	-4.660657	1.949107
**44**	C	6.551600	-2.021941	-1.343820	**91**	H	3.633000	-5.486527	1.373131
**45**	C	-0.757881	3.541233	0.365443	**92**	H	4.961897	-5.029766	2.456511
**46**	H	-1.321495	4.405537	0.031139	**93**	H	4.371790	-3.883946	1.242543
**47**	C	-5.093656	-1.565483	-0.884390	**94**	C	-3.505657	2.976130	-1.112395
**95**	H	-2.563911	3.465574	-1.320360	**118**	H	9.124311	-1.598580	-0.276984

**96**	C	-5.072674	1.313091	3.905928	**119**	H	9.115487	-1.033706	-1.959865
**97**	H	-6.028912	0.802990	3.962746	**120**	C	7.906258	-3.229207	-3.072018
**98**	C	-5.914969	1.674438	-0.641387	**121**	H	7.874496	-2.363944	-3.742062
**99**	H	-6.826797	1.126561	-0.427180	**122**	H	7.065428	-3.880977	-3.327237
**100**	C	-10.025686	-1.871644	0.623109	**123**	H	8.831139	-3.783084	-3.270326
**101**	H	-9.730818	-1.038298	1.269253	**124**	C	7.909534	-4.016732	-0.691646
**102**	H	-9.700547	-2.805153	1.093864	**125**	H	8.834795	-4.578989	-0.863169
**103**	H	-11.119199	-1.885867	0.578158	**126**	H	7.068141	-4.692053	-0.873881
**104**	C	-9.986888	-0.408363	-1.385399	**127**	H	7.877485	-3.720948	0.362075
**105**	H	-11.082444	-0.409718	-1.395505	**128**	C	4.984103	5.923666	1.452986
**106**	H	-9.642732	-0.262789	-2.413665	**129**	H	4.281353	6.232074	0.671671
**107**	H	-9.652464	0.450160	-0.793408	**130**	H	5.618267	6.784528	1.688539
**108**	C	-5.664759	2.221035	-1.898009	**131**	H	4.411073	5.677575	2.352998
**109**	H	-6.407703	2.129456	-2.683515	**132**	C	6.825051	4.405224	2.139527
**110**	C	-4.460032	2.873422	-2.125194	**133**	H	6.275128	4.082564	3.029420
**111**	H	-4.250059	3.317841	-3.093746	**134**	H	7.431183	5.278272	2.407757
**112**	C	-9.949194	-2.904045	-1.637228	**135**	H	7.506933	3.597625	1.857045
**113**	H	-9.585200	-3.852649	-1.230004	**136**	C	6.661359	5.203570	-0.229805
**114**	H	-9.606544	-2.829861	-2.673484	**137**	H	7.335439	4.416719	-0.581162
**115**	H	-11.044320	-2.933379	-1.647629	**138**	H	7.268951	6.083480	0.011244
**116**	C	9.087783	-1.923433	-1.322041	**139**	H	5.992789	5.464999	-1.056631
**117**	H	9.993828	-2.504853	-1.521722	**140**	O	-5.266755	1.182865	1.558881

Table S26. Cartesian coordinates of the optimized S_1 geometry of **2**

**1**	Au	0.992236	-0.523817	-0.812533	**48**	C	4.067606	-2.026275	-1.376041
**2**	N	3.033407	0.048484	-0.459340	**49**	H	3.122836	-2.531609	-1.549208
**3**	N	0.462636	0.894525	-2.259851	**50**	C	-7.940331	-1.478637	-0.938901
**4**	N	-2.750848	2.223377	1.412640	**51**	C	-2.552674	2.056422	3.854209
**5**	C	4.971389	1.222969	-0.067741	**52**	H	-1.561641	2.493462	3.808336
**6**	C	0.868105	-2.035358	0.547100	**53**	C	-3.270370	1.871440	2.668941
**7**	C	-1.644355	-0.183590	-1.908284	**54**	C	2.903717	-4.094852	3.035122
**8**	C	3.543685	1.213703	0.040592	**55**	C	-5.778176	-1.636693	0.164696
**9**	C	5.698104	2.340629	0.269360	**56**	H	-5.213302	-1.716370	1.088782
**10**	H	6.779332	2.334369	0.166596	**57**	C	1.240605	1.843235	-2.834056
**11**	C	-0.441111	-2.565911	0.697277	**58**	H	2.276966	1.854999	-2.511719
**12**	C	4.097258	-0.733818	-0.840465	**59**	C	-2.831666	-2.127003	-0.195591
**13**	C	-0.899259	0.820152	-2.620380	**60**	H	-3.307510	-2.862781	0.446197
**14**	C	5.329881	-0.073787	-0.612366	**61**	C	-3.649071	2.314247	0.328436
**15**	C	-3.034893	-0.410857	-1.921000	**62**	C	-7.169286	-1.644475	0.213818
**16**	H	-3.680522	0.215118	-2.528884	**63**	H	-7.650254	-1.761397	1.179065
**17**	C	2.868122	2.339562	0.569022	**64**	C	-1.396181	1.700151	-3.583617
**18**	C	-0.903373	-0.980700	-1.021378	**65**	H	-2.439898	1.611030	-3.868652
**19**	C	0.817303	1.274190	1.504055	**66**	C	0.774475	2.736955	-3.760732
**20**	H	1.421415	0.444862	1.857501	**67**	H	1.450126	3.471830	-4.183081
**21**	C	1.904485	-2.532515	1.318001	**68**	C	-4.568735	1.352127	2.757697
**22**	H	2.899706	-2.114511	1.200076	**69**	C	-5.865176	-1.321842	-2.203669
**23**	C	-0.552951	1.243364	1.695637	**70**	H	-5.365028	-1.211304	-3.161811
**24**	H	-1.016093	0.376548	2.154626	**71**	C	3.507089	-2.946882	3.859217
**25**	C	3.649697	3.470260	0.864871	**72**	H	3.843582	-2.124731	3.220323
**26**	H	3.131298	4.332147	1.268389	**73**	H	4.371844	-3.302234	4.430930
**27**	C	6.529541	-0.704459	-0.905386	**74**	H	2.771377	-2.546590	4.564410
**28**	H	7.467538	-0.191285	-0.724453	**75**	C	-7.250805	-1.327648	-2.150480
**29**	C	5.035914	3.504330	0.732408	**76**	H	-7.807777	-1.213887	-3.077029
**30**	C	1.705335	-3.574173	2.243295	**77**	C	-3.089826	1.673062	5.078151
**31**	C	0.631839	3.441089	0.467724	**78**	H	-2.505898	1.815282	5.981674
**32**	H	1.087960	4.294261	-0.025217	**79**	C	-4.946581	1.799420	0.491868
**33**	C	-1.450935	-1.936094	-0.166710	**80**	C	-0.593510	2.665249	-4.161970
**34**	C	1.429654	2.353223	0.845718	**81**	H	-0.989826	3.341428	-4.910889
**35**	C	0.414616	-4.083348	2.379979	**82**	C	7.806532	-2.770648	-1.732140
**36**	H	0.216359	-4.884932	3.083477	**83**	C	-9.467436	-1.448116	-0.922657
**37**	C	-0.647837	-3.583253	1.621047	**84**	C	2.519188	-5.224551	3.991540
**38**	H	-1.642196	-4.002928	1.754302	**85**	H	1.782864	-4.894407	4.731736
**39**	C	-1.350932	2.303429	1.249913	**86**	H	3.406575	-5.566276	4.534111
**40**	C	5.863238	4.734208	1.089770	**87**	H	2.105390	-6.084537	3.454903
**41**	C	-3.621707	-1.351318	-1.072460	**88**	C	-4.366121	1.121686	5.143758
**42**	C	5.279006	-2.639581	-1.650834	**89**	H	-4.788625	0.817678	6.095765
**43**	H	5.264658	-3.647261	-2.053645	**90**	C	3.962401	-4.627444	2.055501
**44**	C	6.520839	-2.009074	-1.422207	**91**	H	3.557862	-5.452141	1.459684
**45**	C	-0.739492	3.425299	0.676213	**92**	H	4.836213	-4.997549	2.603929
**46**	H	-1.339174	4.284692	0.398968	**93**	H	4.303708	-3.851305	1.363462
**47**	C	-5.095431	-1.460534	-1.041162	**94**	C	-3.318418	2.851730	-0.916825
**95**	H	-2.311782	3.192435	-1.115317	**118**	H	9.118354	-1.669967	-0.371673

**96**	C	-5.110187	0.974529	3.977425	**119**	H	9.096754	-1.030408	-2.029987
**97**	H	-6.115578	0.566555	3.985745	**120**	C	7.826698	-3.150945	-3.221123
**98**	C	-5.877236	1.834432	-0.533041	**121**	H	7.796635	-2.256967	-3.851930
**99**	H	-6.846555	1.380300	-0.356999	**122**	H	6.976303	-3.782891	-3.492185
**100**	C	-10.034627	-1.619438	0.487521	**123**	H	8.742587	-3.704469	-3.454381
**101**	H	-9.701993	-0.819518	1.157280	**124**	C	7.850215	-4.046612	-0.875315
**102**	H	-9.744143	-2.579345	0.926813	**125**	H	8.767889	-4.607380	-1.082991
**103**	H	-11.128247	-1.588322	0.449900	**126**	H	7.001454	-4.704777	-1.081725
**104**	C	-9.950407	-0.098098	-1.474983	**127**	H	7.832709	-3.800223	0.191157
**105**	H	-11.045473	-0.058227	-1.482569	**128**	C	4.997467	5.900722	1.567889
**106**	H	-9.601717	0.067955	-2.498563	**129**	H	4.281622	6.215303	0.801551
**107**	H	-9.583839	0.728266	-0.856583	**130**	H	5.637008	6.758576	1.796219
**108**	C	-5.539235	2.397249	-1.758423	**131**	H	4.443593	5.649935	2.478330
**109**	H	-6.268146	2.418620	-2.561761	**132**	C	6.850828	4.370606	2.210078
**110**	C	-4.257949	2.903429	-1.942490	**133**	H	6.317768	4.042827	3.108002
**111**	H	-3.964427	3.330055	-2.896039	**134**	H	7.458429	5.243469	2.471342
**112**	C	-10.010950	-2.583131	-1.803528	**135**	H	7.531652	3.568514	1.910194
**113**	H	-9.682400	-3.557316	-1.427744	**136**	C	6.643635	5.189004	-0.154490
**114**	H	-9.669408	-2.490615	-2.838670	**137**	H	7.318406	4.410277	-0.521769
**115**	H	-11.106655	-2.569403	-1.809067	**138**	H	7.249618	6.069020	0.085670
**116**	C	9.058621	-1.945457	-1.429769	**139**	H	5.960681	5.454619	-0.967391
**117**	H	9.949331	-2.534518	-1.668332	**140**	O	-5.350310	1.194356	1.652623

Table S27. Cartesian coordinates of the optimized T_1 geometry of **2**

**1**	Au	0.994188	-0.519780	-0.812114	**48**	C	4.070314	-2.018896	-1.377225
**2**	N	3.032321	0.052223	-0.455327	**49**	H	3.126413	-2.525226	-1.552264
**3**	N	0.462692	0.912677	-2.245931	**50**	C	-7.938566	-1.472810	-0.944353
**4**	N	-2.754271	2.215667	1.422479	**51**	C	-2.556486	2.025016	3.862345
**5**	C	4.968822	1.227884	-0.059940	**52**	H	-1.565699	2.463044	3.820875
**6**	C	0.869916	-2.043640	0.533791	**53**	C	-3.273940	1.851241	2.675233
**7**	C	-1.643098	-0.169787	-1.904017	**54**	C	2.905918	-4.125827	3.002480
**8**	C	3.541200	1.216798	0.048037	**55**	C	-5.775987	-1.640685	0.156976
**9**	C	5.694533	2.345595	0.279797	**56**	H	-5.210713	-1.728700	1.080078
**10**	H	6.775797	2.340443	0.177372	**57**	C	1.241329	1.864612	-2.814115
**11**	C	-0.438804	-2.577160	0.677576	**58**	H	2.277431	1.874520	-2.491039
**12**	C	4.097719	-0.728061	-0.837884	**59**	C	-2.829318	-2.129948	-0.210614
**13**	C	-0.898218	0.839895	-2.608137	**60**	H	-3.305007	-2.871863	0.424225
**14**	C	5.329104	-0.066868	-0.607702	**61**	C	-3.651954	2.315053	0.338583
**15**	C	-3.033763	-0.395923	-1.917778	**62**	C	-7.167079	-1.648651	0.206581
**16**	H	-3.679662	0.237031	-2.518141	**63**	H	-7.647685	-1.773862	1.170964
**17**	C	2.864372	2.341038	0.579169	**64**	C	-1.394803	1.725055	-3.566693
**18**	C	-0.900975	-0.976085	-1.026266	**65**	H	-2.438133	1.636923	-3.853345
**19**	C	0.814265	1.267336	1.505772	**66**	C	0.775421	2.763535	-3.735950
**20**	H	1.418865	0.436117	1.853856	**67**	H	1.451165	3.501123	-4.153440
**21**	C	1.906229	-2.546922	1.300849	**68**	C	-4.572171	1.330778	2.758799
**22**	H	2.900941	-2.126302	1.188288	**69**	C	-5.863944	-1.305302	-2.208582
**23**	C	-0.556011	1.234443	1.697447	**70**	H	-5.364104	-1.186442	-3.165890
**24**	H	-1.018642	0.363597	2.149286	**71**	C	3.508068	-2.985723	3.838281
**25**	C	3.645107	3.472109	0.877275	**72**	H	3.844638	-2.157149	3.207814
**26**	H	3.125970	4.332558	1.282930	**73**	H	4.372596	-3.346292	4.407055
**27**	C	6.530060	-0.694748	-0.902679	**74**	H	2.771663	-2.592799	4.546883
**28**	H	7.467107	-0.180298	-0.720399	**75**	C	-7.249542	-1.311449	-2.154893
**29**	C	5.031105	3.507811	0.745104	**76**	H	-7.806885	-1.189603	-3.080186
**30**	C	1.707551	-3.598290	2.215173	**77**	C	-3.093577	1.629475	5.082409
**31**	C	0.627771	3.443187	0.488326	**78**	H	-2.509837	1.763186	5.987345
**32**	H	1.083366	4.300764	0.002599	**79**	C	-4.949323	1.798134	0.496852
**33**	C	-1.448689	-1.939681	-0.180549	**80**	C	-0.591842	2.693962	-4.138497
**34**	C	1.426553	2.352418	0.856876	**81**	H	-0.987608	3.374204	-4.884034
**35**	C	0.417366	-4.110566	2.345472	**82**	C	7.810541	-2.756227	-1.735542
**36**	H	0.219565	-4.919579	3.040536	**83**	C	-9.465662	-1.442288	-0.927190
**37**	C	-0.645050	-3.604044	1.590857	**84**	C	2.521683	-5.265240	3.947439
**38**	H	-1.639014	-4.026248	1.718969	**85**	H	1.784631	-4.943038	4.690410
**39**	C	-1.354489	2.297809	1.260795	**86**	H	3.409014	-5.611596	4.487152
**40**	C	5.857276	4.737708	1.105236	**87**	H	2.108942	-6.120224	3.402070
**41**	C	-3.620072	-1.345154	-1.078864	**88**	C	-4.369612	1.076832	5.142456
**42**	C	5.282770	-2.629480	-1.653573	**89**	H	-4.792056	0.763287	6.091389
**43**	H	5.270009	-3.636062	-2.059230	**90**	C	3.965438	-4.647937	2.018121
**44**	C	6.523618	-1.997603	-1.423175	**91**	H	3.561751	-5.467005	1.414006
**45**	C	-0.743336	3.425122	0.696630	**92**	H	4.839237	-5.022863	2.563286
**46**	H	-1.343421	4.286587	0.426801	**93**	H	4.306529	-3.864780	1.333938
**47**	C	-5.093772	-1.454219	-1.047616	**94**	C	-3.320497	2.862461	-0.902148
**95**	H	-2.313960	3.205592	-1.096878	**118**	H	9.120404	-1.657688	-0.371524

**96**	C	-5.113532	0.940861	3.974707	**119**	H	9.097924	-1.012976	-2.027785
**97**	H	-6.118778	0.532470	3.978905	**120**	C	7.831413	-3.131679	-3.225729
**98**	C	-5.878974	1.840270	-0.528722	**121**	H	7.799909	-2.235687	-3.853608
**99**	H	-6.848070	1.383969	-0.357113	**122**	H	6.981978	-3.764039	-3.498845
**100**	C	-10.032261	-1.625894	0.481675	**123**	H	8.748161	-3.683012	-3.460853
**101**	H	-9.699348	-0.831836	1.158231	**124**	C	7.856361	-4.034882	-0.882888
**102**	H	-9.741605	-2.589598	0.912454	**125**	H	8.774829	-4.593662	-1.092476
**103**	H	-11.125893	-1.594444	0.444778	**126**	H	7.008525	-4.693639	-1.091241
**104**	C	-9.948722	-0.087423	-1.467459	**127**	H	7.838633	-3.791945	0.184374
**105**	H	-11.043786	-0.047439	-1.474347	**128**	C	4.990344	5.902590	1.585224
**106**	H	-9.600350	0.087607	-2.489648	**129**	H	4.274474	6.217932	0.819215
**107**	H	-9.581961	0.733453	-0.841901	**130**	H	5.629033	6.760601	1.815380
**108**	C	-5.540222	2.412961	-1.749337	**131**	H	4.436357	5.649616	2.494996
**109**	H	-6.268379	2.440069	-2.553191	**132**	C	6.844674	4.372813	2.225272
**110**	C	-4.259191	2.921644	-1.928229	**133**	H	6.311462	4.042923	3.122335
**111**	H	-3.965212	3.356041	-2.878129	**134**	H	7.451546	5.245613	2.488478
**112**	C	-10.009673	-2.569468	-1.817763	**135**	H	7.526176	3.571760	1.924160
**113**	H	-9.681015	-3.546952	-1.450751	**136**	C	6.637863	5.195624	-0.137738
**114**	H	-9.668659	-2.467856	-2.852227	**137**	H	7.313272	4.418082	-0.506353
**115**	H	-11.105377	-2.555613	-1.822634	**138**	H	7.243170	6.075572	0.104406
**116**	C	9.061282	-1.929990	-1.430479	**139**	H	5.955035	5.462407	-0.950370
**117**	H	9.953010	-2.516812	-1.670801	**140**	O	-5.353961	1.184198	1.652439

Table S28. Cartesian coordinates of the optimized T_1' (^{3}CT) geometry of **2**

**1**	Au	1.143897	-0.737983	-0.993994	**48**	C	4.418838	-1.658492	-1.589892
**2**	N	3.024480	0.206161	-0.700034	**49**	H	3.577371	-2.240076	-1.955201
**3**	N	0.414475	0.267486	-2.687867	**50**	C	-7.609211	-1.625340	0.202437
**4**	N	-3.235192	2.020387	0.513307	**51**	C	-3.324849	2.004843	2.965548
**5**	C	4.735376	1.514108	0.116151	**52**	H	-2.265742	2.222200	3.021829
**6**	C	1.247662	-1.958536	0.627603	**53**	C	-3.935114	1.886586	1.708889
**7**	C	-1.545450	-0.912734	-1.993776	**54**	C	3.616483	-3.276506	3.309101
**8**	C	3.320125	1.367309	-0.017550	**55**	C	-5.294200	-1.751914	0.933070
**9**	C	5.290293	2.625800	0.726080	**56**	H	-4.577603	-1.708112	1.748484
**10**	H	6.370781	2.709445	0.807602	**57**	C	1.087057	1.080597	-3.533374
**11**	C	0.008826	-2.561899	0.970745	**58**	H	2.108706	1.306565	-3.242175
**12**	C	4.212678	-0.418455	-0.970111	**59**	C	-2.471193	-2.410509	0.245012
**13**	C	-0.921707	-0.071267	-2.979764	**60**	H	-2.847496	-2.976262	1.092243
**14**	C	5.313994	0.332231	-0.485612	**61**	C	-3.936606	2.000415	-0.690776
**15**	C	-2.923039	-1.134578	-1.789239	**62**	C	-6.652301	-1.650825	1.218059
**16**	H	-3.647159	-0.649539	-2.437092	**63**	H	-6.954157	-1.558680	2.255758
**17**	C	2.470299	2.355908	0.522429	**64**	C	-1.498312	0.400327	-4.163221
**18**	C	-0.681873	-1.461792	-1.029581	**65**	H	-2.510480	0.086560	-4.400209
**19**	C	0.329597	1.199165	1.129108	**66**	C	0.534830	1.594594	-4.678173
**20**	H	0.906118	0.420636	1.616415	**67**	H	1.122669	2.248398	-5.311801
**21**	C	2.371889	-2.198858	1.396179	**68**	C	-5.322472	1.644524	1.669732
**22**	H	3.309486	-1.725614	1.122027	**69**	C	-5.792433	-1.865698	-1.402141
**23**	C	-1.056475	1.100137	1.096540	**70**	H	-5.472165	-1.970050	-2.434926
**24**	H	-1.551929	0.220734	1.495169	**71**	C	4.125602	-1.931549	3.851691
**25**	C	3.073758	3.467149	1.120462	**72**	H	4.335092	-1.223303	3.044305
**26**	H	2.410242	4.217343	1.536462	**73**	H	5.051856	-2.076768	4.419226
**27**	C	6.608847	-0.157205	-0.608799	**74**	H	3.384903	-1.475233	4.516637
**28**	H	7.440229	0.421950	-0.221244	**75**	C	-7.143907	-1.749676	-1.114109
**29**	C	4.460575	3.637033	1.232563	**76**	H	-7.852730	-1.753237	-1.938135
**30**	C	2.329464	-3.044798	2.519413	**77**	C	-4.065685	1.843679	4.126599
**31**	C	0.216915	3.303089	-0.028354	**78**	H	-3.568700	1.934094	5.086598
**32**	H	0.711735	4.153709	-0.486803	**79**	C	-5.328895	1.779131	-0.665651
**33**	C	-1.101673	-2.205947	0.072289	**80**	C	-0.802621	1.231502	-5.019034
**34**	C	0.989910	2.275844	0.524787	**81**	H	-1.253305	1.579391	-5.942000
**35**	C	1.105286	-3.625795	2.850371	**82**	C	8.223845	-2.013823	-1.334731
**36**	H	1.028699	-4.282649	3.710438	**83**	C	-9.099426	-1.428186	0.469669
**37**	C	-0.043467	-3.385549	2.089415	**84**	C	3.408475	-4.227440	4.488350
**38**	H	-0.980595	-3.858669	2.374119	**85**	H	2.679393	-3.831552	5.203175
**39**	C	-1.808144	2.127141	0.531965	**86**	H	4.354597	-4.367148	5.021215
**40**	C	5.085209	4.863589	1.893572	**87**	H	3.064359	-5.212491	4.156349
**41**	C	-3.381320	-1.837882	-0.673351	**88**	C	-5.433696	1.572123	4.066981
**42**	C	5.717885	-2.125246	-1.695915	**89**	H	-6.009277	1.441080	4.976894
**43**	H	5.879837	-3.091938	-2.163072	**90**	C	4.680321	-3.880787	2.377814
**44**	C	6.830675	-1.402226	-1.210491	**91**	H	4.344337	-4.843032	1.978067
**45**	C	-1.172446	3.250513	0.003291	**92**	H	5.614917	-4.045438	2.926363
**46**	H	-1.762735	4.065258	-0.403594	**93**	H	4.899892	-3.224388	1.530134
**47**	C	-4.830742	-1.848074	-0.382814	**94**	C	-3.325730	2.193031	-1.936071
**95**	H	-2.252209	2.309434	-1.998361	**118**	H	9.164911	-0.901755	0.295677

**96**	C -6.061274	1.481389	2.832437		**119**	H	9.348079	-0.139172	-1.298435
**97**	H -7.124041	1.287709	2.737999		**120**	C	8.538676	-2.280853	-2.814886
**98**	C -6.082785	1.758857	-1.828701		**121**	H	8.523378	-1.349306	-3.389501
**99**	H -7.145825	1.562991	-1.744567		**122**	H	7.816724	-2.966511	-3.267485
**100**	C -9.415806	-1.362363	1.964852		**123**	H	9.533446	-2.729058	-2.914552
**101**	H -8.914346	-0.518322	2.450361		**124**	C	8.258680	-3.339338	-0.556227
**102**	H -9.118668	-2.280515	2.481930		**125**	H	9.252232	-3.795607	-0.628828
**103**	H -10.493169	-1.231863	2.107684		**126**	H	7.530502	-4.057019	-0.944988
**104**	C -9.540649	-0.105236	-0.176784		**127**	H	8.033011	-3.173612	0.502252
**105**	H -10.605422	0.077305	0.006998		**128**	C	4.034317	5.858756	2.388835
**106**	H -9.384744	-0.118880	-1.260191		**129**	H	3.411439	6.232062	1.569307
**107**	H -8.969818	0.733751	0.235254		**130**	H	4.532816	6.719529	2.845467
**108**	C -5.462935	1.978138	-3.051078		**131**	H	3.379665	5.414906	3.145980
**109**	H -6.049671	1.971691	-3.963441		**132**	C	5.930187	4.415840	3.096625
**110**	C -4.085300	2.197150	-3.096030		**133**	H	5.309599	3.902030	3.837600
**111**	H -3.582953	2.354076	-4.043676		**134**	H	6.392862	5.284168	3.578736
**112**	C -9.898917	-2.589142	-0.139275		**135**	H	6.731070	3.733100	2.797941
**113**	H -9.602178	-3.542465	0.309317		**136**	C	5.986268	5.580855	0.875996
**114**	H -9.746592	-2.665215	-1.219718		**137**	H	6.786788	4.929294	0.513527
**115**	H -10.970757	-2.446108	0.037299		**138**	H	6.451695	6.459931	1.335445
**116**	C 9.312337	-1.098363	-0.771369		**139**	H	5.405869	5.913484	0.009576
**117**	H 10.289440	-1.577591	-0.887539		**140**	O	-6.001957	1.573747	0.497587

Table S29. Cartesian coordinates of the optimized T_1' (^{3}IL) geometry of **2**

**1**	Au	1.016472	-0.544627	-0.734699	**48**	C	4.099873	-2.087968	-1.329615
**2**	N	3.017042	0.011052	-0.488895	**49**	H	3.169987	-2.627650	-1.487591
**3**	N	0.414115	0.905149	-2.227857	**50**	C	-7.958019	-1.640168	-0.878799
**4**	N	-2.791603	2.436910	1.276051	**51**	C	-2.608505	2.536997	3.721804
**5**	C	4.951341	1.213185	-0.143125	**52**	H	-1.655824	3.047550	3.629541
**6**	C	0.905007	-2.052311	0.594046	**53**	C	-3.291804	2.166811	2.562507
**7**	C	-1.649818	-0.200354	-1.763291	**54**	C	2.976343	-4.164938	2.985529
**8**	C	3.527786	1.212024	-0.039698	**55**	C	-5.820943	-1.749153	0.278592
**9**	C	5.692481	2.342871	0.194061	**56**	H	-5.276331	-1.821350	1.215009
**10**	H	6.775677	2.314064	0.100904	**57**	C	1.186937	1.814778	-2.815965
**11**	C	-0.457840	-2.566970	0.775119	**58**	H	2.228804	1.818299	-2.509987
**12**	C	4.091180	-0.774749	-0.842809	**59**	C	-2.864437	-2.167449	-0.072794
**13**	C	-0.916542	0.815176	-2.515455	**60**	H	-3.329148	-2.924095	0.552159
**14**	C	5.314269	-0.088091	-0.640815	**61**	C	-3.709325	2.405494	0.208743
**15**	C	-3.062447	-0.408728	-1.777684	**62**	C	-7.210889	-1.801792	0.291304
**16**	H	-3.718142	0.242955	-2.343646	**63**	H	-7.712466	-1.948996	1.241458
**17**	C	2.870016	2.356751	0.457555	**64**	C	-1.470699	1.673691	-3.466965
**18**	C	-0.890294	-0.971193	-0.913780	**65**	H	-2.524499	1.596316	-3.704521
**19**	C	0.763659	1.403201	1.426081	**66**	C	0.683265	2.706929	-3.755388
**20**	H	1.350591	0.601140	1.863319	**67**	H	1.338514	3.438162	-4.213811
**21**	C	1.947842	-2.557974	1.318677	**68**	C	-4.527630	1.525931	2.697330
**22**	H	2.941923	-2.150947	1.165226	**69**	C	-5.860963	-1.384386	-2.088486
**23**	C	-0.613703	1.411693	1.595631	**70**	H	-5.343827	-1.229173	-3.031241
**24**	H	-1.100607	0.599124	2.125757	**71**	C	3.647795	-3.032763	3.781297
**25**	C	3.655505	3.471804	0.764410	**72**	H	3.973413	-2.216339	3.130134
**26**	H	3.136743	4.341256	1.154789	**73**	H	4.531482	-3.414303	4.304588
**27**	C	6.533821	-0.717429	-0.911321	**74**	H	2.960475	-2.619061	4.526324
**28**	H	7.458343	-0.174136	-0.742656	**75**	C	-7.245529	-1.441503	-2.070575
**29**	C	5.053824	3.500239	0.640601	**76**	H	-7.783935	-1.324094	-3.007181
**30**	C	1.760326	-3.613914	2.256003	**77**	C	-3.126669	2.233338	4.978056
**31**	C	0.635212	3.471266	0.217997	**78**	H	-2.569258	2.516900	5.865139
**32**	H	1.117119	4.276567	-0.329228	**79**	C	-4.946700	1.769098	0.411100
**33**	C	-1.433259	-1.971711	-0.029538	**80**	C	-0.666735	2.622681	-4.084942
**34**	C	1.412325	2.409376	0.691710	**81**	H	-1.093545	3.295901	-4.822227
**35**	C	0.438453	-4.103943	2.447948	**82**	C	7.857217	-2.782848	-1.658996
**36**	H	0.267211	-4.898190	3.166707	**83**	C	-9.484826	-1.654869	-0.899417
**37**	C	-0.637924	-3.606563	1.751458	**84**	C	2.611407	-5.289457	3.956067
**38**	H	-1.631981	-4.007297	1.928239	**85**	H	1.921759	-4.946008	4.734356
**39**	C	-1.384867	2.460463	1.084394	**86**	H	3.516610	-5.653113	4.452365
**40**	C	5.882786	4.736018	0.996358	**87**	H	2.153309	-6.138800	3.438536
**41**	C	-3.649918	-1.397326	-0.910974	**88**	C	-4.348494	1.579024	5.094648
**42**	C	5.323239	-2.687330	-1.581001	**89**	H	-4.756137	1.339264	6.071317
**43**	H	5.325342	-3.710646	-1.947152	**90**	C	3.973715	-4.715935	1.950614
**44**	C	6.557874	-2.028389	-1.377015	**91**	H	3.525720	-5.535573	1.379268
**45**	C	-0.739268	3.516592	0.436348	**92**	H	4.866611	-5.099920	2.456681
**46**	H	-1.308142	4.381698	0.113584	**93**	H	4.294681	-3.947297	1.240925
**47**	C	-5.117009	-1.531328	-0.909175	**94**	C	-3.474177	2.972923	-1.044392
**95**	H	-2.525040	3.448903	-1.249050	**118**	H	9.145199	-1.602462	-0.346389

**96**	C	-5.056499	1.235836	3.944871	**119**	H	9.109578	-1.030484	-2.026497
**97**	H	-6.016300	0.731506	3.992024	**120**	C	7.890179	-3.224553	-3.129957
**98**	C	-5.902249	1.701740	-0.588068	**121**	H	7.845751	-2.356504	-3.795650
**99**	H	-6.821356	1.163355	-0.381065	**122**	H	7.047632	-3.877816	-3.375499
**100**	C	-10.080323	-1.880471	0.491282	**123**	H	8.813710	-3.774664	-3.344422
**101**	H	-9.792089	-1.087507	1.189135	**124**	C	7.931171	-4.022393	-0.753549
**102**	H	-9.770040	-2.841727	0.913768	**125**	H	8.854883	-4.581706	-0.942197
**103**	H	-11.172891	-1.883671	0.425819	**126**	H	7.088542	-4.698847	-0.925599
**104**	C	-9.992378	-0.304471	-1.428754	**127**	H	7.915291	-3.731320	0.301840
**105**	H	-11.087557	-0.294682	-1.457987	**128**	C	5.014120	5.906166	1.462297
**106**	H	-9.628499	-0.103870	-2.440838	**129**	H	4.302670	6.215288	0.689185
**107**	H	-9.661155	0.515582	-0.782878	**130**	H	5.650102	6.767151	1.692474
**108**	C	-5.650758	2.273234	-1.833271	**131**	H	4.451070	5.658077	2.368043
**109**	H	-6.399616	2.211675	-2.616053	**132**	C	6.863266	4.387576	2.126281
**110**	C	-4.435540	2.908013	-2.053558	**133**	H	6.322988	4.062658	3.021250
**111**	H	-4.221346	3.367153	-3.014211	**134**	H	7.471650	5.260456	2.389949
**112**	C	-9.971440	-2.781758	-1.823141	**135**	H	7.542608	3.581017	1.834846
**113**	H	-9.623352	-3.755743	-1.464902	**136**	C	6.674041	5.190720	-0.239485
**114**	H	-9.609482	-2.651585	-2.847255	**137**	H	7.344977	4.405117	-0.599538
**115**	H	-11.066409	-2.799600	-1.854579	**138**	H	7.283472	6.070615	-0.003040
**116**	C	9.093897	-1.922993	-1.392165	**139**	H	5.996611	5.453311	-1.058692
**117**	H	9.998472	-2.500986	-1.607860	**140**	O	-5.248653	1.146506	1.595545

Table S30. Cartesian coordinates of the optimized S_0 geometry of **3**

**1**	Au	1.146425	-0.431848	-0.919906	**48**	H	-5.383538	-0.477689	1.223050
**2**	O	-1.099894	-0.740501	2.801040	**49**	C	-3.824366	-3.146890	2.617247
**3**	O	-2.218952	3.106101	0.195119	**50**	H	-3.995632	-4.187990	2.871201
**4**	N	-3.057463	0.884722	1.675003	**51**	C	-2.357505	-1.240976	2.544382
**5**	N	3.061471	0.012028	-0.220085	**52**	C	3.021817	3.265054	1.581849
**6**	N	0.805351	1.394051	-2.044285	**53**	H	2.343524	3.958708	2.072636
**7**	C	3.349804	1.187383	0.439834	**54**	C	-4.831357	-2.379912	2.043085
**8**	C	-1.382224	0.434668	-2.000611	**55**	H	-5.800596	-2.815684	1.821921
**9**	C	-3.964914	1.913554	1.402421	**56**	C	4.398620	3.570519	1.525492
**10**	C	-5.273648	1.945864	1.887509	**57**	C	4.744720	1.459070	0.398244
**11**	H	-5.614895	1.157584	2.546841	**58**	C	-4.382466	4.026072	0.275606
**12**	C	4.258634	-0.498293	-0.666383	**59**	H	-3.991991	4.835914	-0.332197
**13**	C	5.338447	0.347690	-0.300523	**60**	C	1.986356	-6.336679	2.452273
**14**	C	-0.504291	1.552725	-2.375988	**61**	H	1.170635	-6.199810	3.170012
**15**	C	1.750594	-2.968825	0.618815	**62**	H	2.796127	-6.862998	2.967363
**16**	H	2.756984	-2.574652	0.719819	**63**	H	1.630055	-6.983999	1.644088
**17**	C	0.010985	3.725562	-3.265046	**64**	C	-0.856942	-3.916055	0.363224
**18**	H	-0.306717	4.648070	-3.741004	**65**	H	-1.873861	-4.291839	0.288134
**19**	C	-3.374502	-0.445125	1.990239	**66**	C	-6.143592	2.970762	1.524205
**20**	C	-2.777381	0.403840	-2.101351	**67**	H	-7.162735	2.955937	1.897544
**21**	H	-3.321357	1.228940	-2.551017	**68**	C	-1.696416	1.191090	1.531655
**22**	C	-3.519696	3.002634	0.631544	**69**	C	5.811887	-1.989420	-1.704804
**23**	C	2.501954	-4.998981	1.919856	**70**	H	5.998350	-2.907608	-2.255665
**24**	C	5.255279	2.641550	0.943060	**71**	C	0.808484	-2.227842	-0.065667
**25**	H	6.323359	2.828202	0.894046	**72**	C	0.624660	0.648247	1.939434
**26**	C	-0.919213	2.730328	-2.993715	**73**	H	1.355699	-0.033585	2.359235
**27**	H	-1.963552	2.864852	-3.249576	**74**	C	3.004769	-4.163212	3.107394
**28**	C	1.346813	3.534355	-2.923928	**75**	H	3.427676	-3.209028	2.779739
**29**	H	2.099317	4.289030	-3.120184	**76**	H	3.785891	-4.707435	3.649666
**30**	C	-0.745274	-0.651582	-1.408560	**77**	H	2.188589	-3.948453	3.804864
**31**	C	0.053152	2.647876	0.710928	**78**	C	-2.813934	-1.767378	-0.999884
**32**	H	0.333137	3.531566	0.147375	**79**	H	-3.391179	-2.562506	-0.537286
**33**	C	-3.495095	-0.684459	-1.589660	**80**	C	-7.115405	-1.790362	-1.474123
**34**	C	0.107155	-4.668622	1.037510	**81**	H	-7.662690	-2.724553	-1.539110
**35**	H	-0.185337	-5.625046	1.457177	**82**	C	4.888062	4.891664	2.119624
**36**	C	-0.527072	-2.688030	-0.197537	**83**	C	-1.424982	-1.758852	-0.898739
**37**	C	1.419153	-4.214938	1.182496	**84**	C	4.501324	-1.675250	-1.385067
**38**	C	1.029019	1.799749	1.248019	**85**	H	3.681579	-2.326820	-1.676816
**39**	C	1.703890	2.343002	-2.308326	**86**	C	6.907662	-1.169250	-1.346719
**40**	H	2.721654	2.133997	-1.994453	**87**	C	-5.733657	-1.819025	-1.637438
**41**	C	-0.725762	0.370467	2.094278	**88**	H	-5.241267	-2.768043	-1.830320
**42**	C	-4.971378	-0.650798	-1.556806	**89**	C	-5.652895	0.554902	-1.342029
**43**	C	-2.576866	-2.572165	2.852802	**90**	H	-5.096838	1.481504	-1.235527
**44**	H	-1.750577	-3.143241	3.262064	**91**	C	-7.029754	0.577523	-1.181156
**45**	C	6.650587	0.004457	-0.645028	**92**	H	-7.507464	1.532344	-0.981524
**46**	H	7.463119	0.662792	-0.353935	**93**	C	-5.710528	4.002127	0.699694
**47**	C	-4.607056	-1.043538	1.721565	**94**	H	-6.385969	4.798684	0.405584
**95**	C	8.320018	-1.604693	-1.739044	**118**	C	-9.969141	-1.897408	-1.093264

**96**	C	4.195752	6.060691	1.402210	**119**	H	-9.838288	-2.332759	-2.089381
**97**	H	4.432400	6.053074	0.333031	**120**	H	-11.044047	-1.797317	-0.912963
**98**	H	4.527697	7.018171	1.819787	**121**	H	-9.573593	-2.601865	-0.354202
**99**	H	3.107582	6.010056	1.505478	**122**	C	9.381996	-0.604097	-1.279525
**100**	C	-7.795572	-0.594718	-1.226960	**123**	H	9.228015	0.383109	-1.727778
**101**	C	-9.946694	0.411625	-2.014243	**124**	H	10.374267	-0.955450	-1.580914
**102**	H	-9.537600	1.424351	-1.953658	**125**	H	9.385685	-0.490353	-0.190464
**103**	H	-11.026801	0.476308	-1.842940	**126**	C	-9.547713	0.021560	0.431704
**104**	H	-9.783047	0.042816	-3.031781	**127**	H	-9.098115	-0.633718	1.184999
**105**	C	3.669694	-5.274338	0.958801	**128**	H	-10.622691	0.087664	0.632622
**106**	H	3.335722	-5.870982	0.103579	**129**	H	-9.119312	1.020566	0.557714
**107**	H	4.460531	-5.829530	1.475284	**130**	C	8.633519	-2.963988	-1.095035
**108**	H	4.108222	-4.348924	0.573193	**131**	H	8.582744	-2.895382	-0.003568
**109**	C	4.548274	4.941488	3.617084	**132**	H	9.640558	-3.296909	-1.371973
**110**	H	3.470962	4.862405	3.790560	**133**	H	7.926198	-3.735122	-1.414604
**111**	H	4.890144	5.885723	4.056403	**134**	C	2.472776	2.090515	1.078336
**112**	H	5.034239	4.118746	4.151180	**135**	C	8.413721	-1.732607	-3.267213
**113**	C	-9.301073	-0.525974	-0.983233	**136**	H	7.702026	-2.466783	-3.656355
**114**	C	6.400021	5.066702	1.967295	**137**	H	9.419604	-2.051183	-3.564354
**115**	H	6.952547	4.278157	2.488606	**138**	H	8.201616	-0.772877	-3.749502
**116**	H	6.705075	6.026251	2.397343	**139**	C	-1.289217	2.317158	0.825331
**117**	H	6.703682	5.061062	0.915170					

Table S31. Cartesian coordinates of the optimized S_1 geometry of **3**

**1**	Au	1.107975	-0.376948	-0.971996	**48**	H	-5.281656	-0.503065	1.228620
**2**	O	-0.978151	-0.787527	2.724761	**49**	C	-3.735974	-3.135555	2.703972
**3**	O	-2.168566	3.166494	0.313683	**50**	H	-3.915331	-4.169816	2.978183
**4**	N	-2.958862	0.862387	1.673452	**51**	C	-2.247936	-1.260626	2.547811
**5**	N	3.060092	0.044861	-0.240717	**52**	C	3.071279	3.324110	1.492750
**6**	N	0.792337	1.419808	-2.024228	**53**	H	2.401475	4.050871	1.944453
**7**	C	3.375972	1.187184	0.440945	**54**	C	-4.745222	-2.376125	2.112821
**8**	C	-1.427267	0.528690	-1.991629	**55**	H	-5.712448	-2.817144	1.897065
**9**	C	-3.892968	1.878238	1.428622	**56**	C	4.447781	3.590172	1.451245
**10**	C	-5.202336	1.865291	1.919696	**57**	C	4.781454	1.422993	0.423765
**11**	H	-5.520898	1.066863	2.576724	**58**	C	-4.361278	3.994493	0.329359
**12**	C	4.248113	-0.513449	-0.654558	**59**	H	-3.991779	4.822937	-0.264984
**13**	C	5.353520	0.284047	-0.259601	**60**	C	1.933671	-6.331842	2.385915
**14**	C	-0.566744	1.624566	-2.339040	**61**	H	1.113856	-6.193909	3.098920
**15**	C	1.713795	-2.953643	0.564432	**62**	H	2.738507	-6.863254	2.903943
**16**	H	2.725241	-2.573540	0.674839	**63**	H	1.578540	-6.974849	1.573910
**17**	C	-0.042842	3.852066	-3.134595	**64**	C	-0.888642	-3.888997	0.290088
**18**	H	-0.357653	4.797906	-3.561279	**65**	H	-1.905586	-4.262727	0.199662
**19**	C	-3.272873	-0.465983	2.008124	**66**	C	-6.090858	2.873805	1.575579
**20**	C	-2.834853	0.458664	-2.075359	**67**	H	-7.106291	2.836825	1.955228
**21**	H	-3.393705	1.281398	-2.513174	**68**	C	-1.621122	1.186173	1.538643
**22**	C	-3.467091	2.996325	0.689130	**69**	C	5.752287	-2.060766	-1.674669
**23**	C	2.455549	-4.994162	1.859429	**70**	H	5.911833	-2.980826	-2.229538
**24**	C	5.302283	2.606674	0.927993	**71**	C	0.784795	-2.192922	-0.122670
**25**	H	6.373240	2.776056	0.890252	**72**	C	0.708126	0.651290	1.909026
**26**	C	-0.958619	2.850656	-2.890666	**73**	H	1.453920	-0.042186	2.279186
**27**	H	-2.009627	3.002141	-3.119424	**74**	C	2.959443	-4.167153	3.052516
**28**	C	1.329269	3.614092	-2.820576	**75**	H	3.386009	-3.212885	2.729573
**29**	H	2.092025	4.360842	-3.007485	**76**	H	3.737183	-4.715845	3.595649
**30**	C	-0.793042	-0.584621	-1.408036	**77**	H	2.141804	-3.952263	3.748609
**31**	C	0.099764	2.716205	0.783356	**78**	C	-2.844798	-1.755806	-1.041190
**32**	H	0.369311	3.602992	0.221452	**79**	H	-3.407324	-2.572913	-0.601759
**33**	C	-3.532844	-0.650337	-1.591588	**80**	C	-7.145753	-1.795898	-1.442166
**34**	C	0.067876	-4.654023	0.964386	**81**	H	-7.682051	-2.736687	-1.507919
**35**	H	-0.229582	-5.614883	1.371100	**82**	C	4.964280	4.921932	1.994920
**36**	C	-0.551800	-2.655851	-0.253879	**83**	C	-1.455446	-1.714143	-0.933266
**37**	C	1.378195	-4.202548	1.120789	**84**	C	4.449571	-1.692146	-1.380737
**38**	C	1.086309	1.838910	1.258417	**85**	H	3.605760	-2.298887	-1.696350
**39**	C	1.681612	2.404144	-2.281111	**86**	C	6.871821	-1.290870	-1.285923
**40**	H	2.712035	2.180783	-2.022999	**87**	C	-5.764889	-1.807213	-1.618722
**41**	C	-0.627458	0.345154	2.059504	**88**	H	-5.263585	-2.748784	-1.823735
**42**	C	-5.010334	-0.632213	-1.541750	**89**	C	-5.708962	0.562179	-1.312671
**43**	C	-2.479745	-2.579270	2.906260	**90**	H	-5.161366	1.495283	-1.219891
**44**	H	-1.651170	-3.155114	3.302366	**91**	C	-7.085159	0.569329	-1.140909
**45**	C	6.652572	-0.105329	-0.581374	**92**	H	-7.574415	1.518745	-0.940770
**46**	H	7.487485	0.516737	-0.276162	**93**	C	-5.682548	3.923194	0.753742
**47**	C	-4.516507	-1.055035	1.758450	**94**	H	-6.380930	4.703211	0.469943
**95**	C	8.272429	-1.783616	-1.646952	**118**	C	-9.996760	-1.938991	-1.042169

**96**	C	4.305208	6.073415	1.219771	**119**	H	-9.862866	-2.376113	-2.036982
**97**	H	4.546897	6.012424	0.153880	**120**	H	-11.072476	-1.852566	-0.859064
**98**	H	4.660621	7.038354	1.598006	**121**	H	-9.589181	-2.635797	-0.302290
**99**	H	3.215706	6.056648	1.317490	**122**	C	9.365665	-0.831765	-1.158020
**100**	C	-7.839648	-0.610287	-1.186009	**123**	H	9.267317	0.162437	-1.606327
**101**	C	-10.004525	0.365176	-1.974470	**124**	H	10.347515	-1.226376	-1.438183
**102**	H	-9.607102	1.382948	-1.919822	**125**	H	9.350348	-0.722456	-0.068562
**103**	H	-11.085476	0.417786	-1.803378	**126**	C	-9.600669	-0.007542	0.472363
**104**	H	-9.836029	-0.006730	-2.989977	**127**	H	-9.142797	-0.653052	1.229322
**105**	C	3.626419	-5.272638	0.903146	**128**	H	-10.676418	0.045692	0.673492
**106**	H	3.291984	-5.863123	0.043920	**129**	H	-9.186093	0.998140	0.592803
**107**	H	4.413181	-5.834305	1.419467	**130**	C	8.508312	-3.158032	-1.000998
**108**	H	4.069875	-4.347506	0.522429	**131**	H	8.433371	-3.092160	0.089222
**109**	C	4.609719	5.040146	3.485425	**132**	H	9.506955	-3.531201	-1.254633
**110**	H	3.528893	4.997847	3.649543	**133**	H	7.777770	-3.897011	-1.342948
**111**	H	4.972551	5.992631	3.887519	**134**	C	2.509768	2.124067	1.049737
**112**	H	5.068671	4.228827	4.059400	**135**	C	8.393557	-1.909593	-3.173535
**113**	C	-9.345673	-0.558793	-0.939480	**136**	H	7.662934	-2.613592	-3.582220
**114**	C	6.481633	5.054894	1.851646	**137**	H	9.392554	-2.267495	-3.446912
**115**	H	7.012290	4.280609	2.415430	**138**	H	8.232131	-0.940749	-3.656805
**116**	H	6.802071	6.026026	2.241693	**139**	C	-1.229824	2.368907	0.893941
**117**	H	6.796470	4.997258	0.804500					

Table S32. Cartesian coordinates of the optimized T_1 geometry of **3**

**1**	Au	1.133497	-0.390197	-0.985168	**48**	H	-5.321718	-0.542595	1.213830
**2**	O	-1.010005	-0.829242	2.686862	**49**	C	-3.765213	-3.179617	2.670643
**3**	O	-2.217290	3.137860	0.304328	**50**	H	-3.942402	-4.214932	2.942303
**4**	N	-2.996360	0.821311	1.647714	**51**	C	-2.279402	-1.302821	2.513521
**5**	N	3.072249	0.037146	-0.235482	**52**	C	3.003717	3.323709	1.482071
**6**	N	0.824043	1.420448	-2.020625	**53**	H	2.315474	4.042026	1.919465
**7**	C	3.359308	1.186916	0.446821	**54**	C	-4.778896	-2.418844	2.088293
**8**	C	-1.399030	0.538186	-1.989964	**55**	H	-5.747089	-2.860004	1.877297
**9**	C	-3.932026	1.840928	1.424065	**56**	C	4.376539	3.612163	1.461043
**10**	C	-5.235958	1.824050	1.928964	**57**	C	4.760494	1.445769	0.449281
**11**	H	-5.549061	1.017981	2.579252	**58**	C	-4.407507	3.971521	0.356158
**12**	C	4.275205	-0.501677	-0.632006	**59**	H	-4.042930	4.806698	-0.231764
**13**	C	5.360953	0.315537	-0.222838	**60**	C	1.938717	-6.372333	2.325686
**14**	C	-0.535782	1.636294	-2.320761	**61**	H	1.118882	-6.238676	3.039472
**15**	C	1.730863	-2.980063	0.529377	**62**	H	2.741793	-6.910468	2.839464
**16**	H	2.743770	-2.604390	0.641246	**63**	H	1.582397	-7.007056	1.507682
**17**	C	-0.007936	3.877155	-3.074526	**64**	C	-0.876893	-3.901760	0.253979
**18**	H	-0.321245	4.832957	-3.479874	**65**	H	-1.895639	-4.270375	0.162825
**19**	C	-3.307943	-0.508016	1.980901	**66**	C	-6.125644	2.839012	1.607895
**20**	C	-2.807187	0.475486	-2.071535	**67**	H	-7.136742	2.799762	1.998681
**21**	H	-3.362748	1.304701	-2.501086	**68**	C	-1.660329	1.146605	1.507528
**22**	C	-3.511684	2.966693	0.693149	**69**	C	5.820731	-2.022909	-1.629702
**23**	C	2.464613	-5.031419	1.811560	**70**	H	6.004038	-2.940169	-2.182043
**24**	C	5.254200	2.640887	0.956442	**71**	C	0.803860	-2.211093	-0.151103
**25**	H	6.322792	2.827465	0.933907	**72**	C	0.670944	0.619659	1.878952
**26**	C	-0.926618	2.875834	-2.846442	**73**	H	1.420119	-0.069087	2.251064
**27**	H	-1.978210	3.036714	-3.065297	**74**	C	2.968350	-4.215455	3.012330
**28**	C	1.364621	3.628818	-2.772613	**75**	H	3.397075	-3.259313	2.697900
**29**	H	2.129020	4.376393	-2.948914	**76**	H	3.744333	-4.770213	3.551792
**30**	C	-0.767400	-0.587696	-1.425194	**77**	H	2.150170	-4.004972	3.709125
**31**	C	0.054594	2.685993	0.756309	**78**	C	-2.825382	-1.747726	-1.054800
**32**	H	0.320867	3.577674	0.200580	**79**	H	-3.390787	-2.564255	-0.618095
**33**	C	-3.509473	-0.634937	-1.597327	**80**	C	-7.122312	-1.777333	-1.440698
**34**	C	0.077367	-4.675197	0.921522	**81**	H	-7.658703	-2.718408	-1.501307
**35**	H	-0.223665	-5.637233	1.322776	**82**	C	4.861676	4.955891	2.004595
**36**	C	-0.535165	-2.666736	-0.283205	**83**	C	-1.435617	-1.716268	-0.954174
**37**	C	1.390270	-4.230830	1.078300	**84**	C	4.507845	-1.677045	-1.354220
**38**	C	1.045529	1.810198	1.226815	**85**	H	3.679436	-2.298202	-1.682678
**39**	C	1.715349	2.405984	-2.260267	**86**	C	6.921455	-1.233324	-1.226133
**40**	H	2.746167	2.173307	-2.012247	**87**	C	-5.742093	-1.789223	-1.620416
**41**	C	-0.662161	0.307276	2.026657	**88**	H	-5.241728	-2.731799	-1.822641
**42**	C	-4.986758	-0.614030	-1.548580	**89**	C	-5.685462	0.581394	-1.325372
**43**	C	-2.508441	-2.623306	2.868283	**90**	H	-5.138931	1.515526	-1.239080
**44**	H	-1.677537	-3.199590	3.258833	**91**	C	-7.061309	0.589281	-1.150985
**45**	C	6.671582	-0.051941	-0.526303	**92**	H	-7.550184	1.539656	-0.954995
**46**	H	7.490979	0.585182	-0.210011	**93**	C	-5.723832	3.897921	0.794894
**47**	C	-4.553135	-1.096617	1.736631	**94**	H	-6.423337	4.683350	0.529359
**95**	C	8.335323	-1.702043	-1.567694	**118**	C	-9.971411	-1.919286	-1.025625

**96**	C	4.196925	6.091286	1.210831	**119**	H	-9.842493	-2.362154	-2.018547
**97**	H	4.457268	6.027027	0.149505	**120**	H	-11.046193	-1.832233	-0.837487
**98**	H	4.529752	7.064692	1.588054	**121**	H	-9.559702	-2.611585	-0.283787
**99**	H	3.106328	6.056778	1.290434	**122**	C	9.405000	-0.730575	-1.065436
**100**	C	-7.815607	-0.590634	-1.187222	**123**	H	9.294893	0.261278	-1.516218
**101**	C	-9.984409	0.379436	-1.971659	**124**	H	10.397433	-1.107954	-1.331875
**102**	H	-9.586950	1.397587	-1.924906	**125**	H	9.372967	-0.620347	0.023565
**103**	H	-11.064524	0.432836	-1.795684	**126**	C	-9.568667	0.021441	0.475445
**104**	H	-9.820729	0.001526	-2.985728	**127**	H	-9.106514	-0.619210	1.233973
**105**	C	3.636618	-5.304421	0.855103	**128**	H	-10.643402	0.075180	0.681722
**106**	H	3.302224	-5.884941	-0.010895	**129**	H	-9.154067	1.028071	0.587565
**107**	H	4.420006	-5.874108	1.367704	**130**	C	8.586916	-3.071279	-0.916830
**108**	H	4.084610	-4.377096	0.485217	**131**	H	8.496362	-3.005338	0.172215
**109**	C	4.480459	5.078228	3.488113	**132**	H	9.595143	-3.427541	-1.156690
**110**	H	3.397901	5.018404	3.634593	**133**	H	7.873754	-3.823195	-1.267226
**111**	H	4.820149	6.039431	3.889807	**134**	C	2.468168	2.112696	1.036446
**112**	H	4.943401	4.278618	4.075190	**135**	C	8.479683	-1.827671	-3.092256
**113**	C	-9.320449	-0.538250	-0.934340	**136**	H	7.767097	-2.544840	-3.509869
**114**	C	6.378796	5.113070	1.885800	**137**	H	9.488431	-2.168263	-3.351788
**115**	H	6.912400	4.350691	2.462889	**138**	H	8.307702	-0.862344	-3.578955
**116**	H	6.676886	6.091586	2.275222	**139**	C	-1.273562	2.335335	0.868870
**117**	H	6.711955	5.054386	0.844394					

Table S33. Cartesian coordinates of the optimized T_1' (^{3}CT) geometry of **3**

**1**	Au	-0.884832	-1.052430	-0.384872	**48**	C	4.360639	3.992912	-0.944084
**2**	O	1.269750	3.437927	0.868069	**49**	H	4.763796	4.714796	-1.646525
**3**	O	1.272783	-0.914523	2.840952	**50**	C	2.554873	3.234708	0.447995
**4**	N	2.721751	1.244702	1.787548	**51**	C	-3.776201	2.128799	2.207651
**5**	N	-2.939135	-0.872622	0.162690	**52**	H	-3.306449	2.906167	2.799841
**6**	N	-0.357414	-2.815847	0.632739	**53**	C	5.109834	2.881230	-0.561469
**7**	C	-3.533146	0.154891	0.868375	**54**	H	6.093722	2.704485	-0.982836
**8**	C	1.776622	-2.145524	-0.213367	**55**	C	-5.169571	2.139608	2.025551
**9**	C	3.379924	0.247920	2.511919	**56**	C	-4.946860	0.131704	0.701306
**10**	C	4.737292	0.300219	2.850735	**57**	C	3.236698	-1.928273	3.595974
**11**	H	5.314869	1.183047	2.610022	**58**	H	2.614867	-2.771038	3.877386
**12**	C	-3.946064	-1.575510	-0.440268	**59**	C	0.809640	2.177391	-2.527078
**13**	C	-5.219790	-1.012716	-0.145239	**60**	H	1.818649	2.448451	-2.828165
**14**	C	1.023289	-3.092891	0.561672	**61**	C	5.342413	-0.764621	3.502826
**15**	C	-1.778901	1.532390	-1.750021	**62**	H	6.398589	-0.705954	3.743661
**16**	H	-2.785329	1.279448	-1.431067	**63**	C	1.325757	1.265066	1.858845
**17**	C	0.719973	-4.981847	2.048435	**64**	C	-5.016216	-3.259682	-1.762457
**18**	H	1.127076	-5.827282	2.591640	**65**	H	-4.934629	-4.136025	-2.394647
**19**	C	3.319050	2.136789	0.885901	**66**	C	-0.747160	0.651392	-1.480715
**20**	C	3.172665	-2.085318	-0.405640	**67**	C	-0.775708	2.349061	1.343030
**21**	H	3.811597	-2.862185	0.005424	**68**	H	-1.297905	3.172702	0.871995
**22**	C	2.625767	-0.852560	2.968142	**69**	C	2.965282	-0.009390	-1.678976
**23**	C	-5.747721	1.110229	1.270004	**70**	H	3.446480	0.831685	-2.167552
**24**	H	-6.822540	1.083953	1.112037	**71**	C	7.240913	-0.213339	-2.372150
**25**	C	1.535758	-4.182521	1.271758	**72**	H	7.678906	0.224786	-3.262742
**26**	H	2.600731	-4.387145	1.206306	**73**	C	1.583160	-0.037048	-1.502887
**27**	C	-0.671796	-4.673424	2.113808	**74**	C	-3.845610	-2.704963	-1.259919
**28**	H	-1.356167	-5.268848	2.707009	**75**	H	-2.874992	-3.133073	-1.492835
**29**	C	1.032603	-1.103175	-0.795363	**76**	C	-6.286713	-2.724788	-1.481541
**30**	C	-0.768369	0.170199	2.391617	**77**	C	5.859308	-0.380743	-2.327655
**31**	H	-1.280887	-0.706082	2.771383	**78**	H	5.258548	-0.078257	-3.180665
**32**	C	3.758527	-1.035757	-1.116133	**79**	C	6.050062	-1.346459	-0.148509
**33**	C	-0.241317	3.067116	-2.768908	**80**	H	5.595103	-1.758084	0.747802
**34**	H	-0.020436	4.008272	-3.261322	**81**	C	7.426086	-1.177038	-0.197548
**35**	C	0.577167	0.967095	-1.883971	**82**	H	8.018169	-1.489361	0.659008
**36**	C	-1.549263	2.764315	-2.389641	**83**	C	4.602190	-1.896235	3.846579
**37**	C	-1.470905	1.239970	1.831243	**84**	H	5.080128	-2.738361	4.335613
**38**	C	-1.145357	-3.602461	1.402722	**85**	C	8.058482	-0.598824	-1.306800
**39**	H	-2.196385	-3.328922	1.421358	**86**	C	-2.939989	1.162525	1.650748
**40**	C	0.607009	2.353955	1.365062	**87**	C	0.618339	0.183326	2.383483
**41**	C	5.230305	-0.942680	-1.213076	**88**	C	9.573488	-0.405681	-1.305604
**42**	C	3.073537	4.157946	-0.446980	**89**	C	9.962190	0.511782	-0.135889
**43**	H	2.439403	4.982721	-0.753047	**90**	H	9.484478	1.492090	-0.235614
**44**	C	-6.369574	-1.584234	-0.660513	**91**	H	9.661583	0.087907	0.826984
**45**	H	-7.338503	-1.148339	-0.431948	**92**	H	11.047413	0.660919	-0.112607
**46**	C	4.593452	1.958891	0.336587	**93**	C	10.262399	-1.768082	-1.136080
**47**	H	5.161457	1.066699	0.560121	**94**	H	10.001606	-2.440327	-1.959565
**95**	H	11.351060	-1.644715	-1.125632	**118**	H	-7.991781	-4.496313	-0.242855

**96**	H	9.971791	-2.255153	-0.200582
**97**	C	10.076854	0.229943	-2.602608
**98**	H	9.847598	-0.392193	-3.473845
**99**	H	9.641826	1.221900	-2.763613
**100**	H	11.164029	0.348314	-2.555627
**101**	C	-2.729663	3.709096	-2.611905
**102**	C	-2.316118	4.992636	-3.333145
**103**	H	-1.895921	4.782103	-4.322108
**104**	H	-3.190973	5.635973	-3.472344
**105**	H	-1.575513	5.558577	-2.758209
**106**	C	-3.334649	4.091344	-1.250634
**107**	H	-3.667222	3.212775	-0.689600
**108**	H	-2.601126	4.627342	-0.637944
**109**	H	-4.200840	4.748692	-1.390732
**110**	C	-3.798781	2.999590	-3.456866
**111**	H	-3.393016	2.707389	-4.430644
**112**	H	-4.170497	2.097028	-2.963114
**113**	H	-4.652641	3.665435	-3.625945
**114**	C	-7.571770	-3.332614	-2.038759
**115**	C	-8.483124	-3.747956	-0.872636
**116**	H	-8.746774	-2.896071	-0.238891
**117**	H	-9.413885	-4.180121	-1.256395
**119**	C	-7.307713	-4.566692	-2.903252
**120**	H	-6.686775	-4.328252	-3.772797
**121**	H	-6.815869	-5.362736	-2.334883
**122**	H	-8.258521	-4.962530	-3.273655
**123**	C	-8.293267	-2.285119	-2.902055
**124**	H	-8.551319	-1.391793	-2.325700
**125**	H	-7.663935	-1.973444	-3.741574
**126**	H	-9.222042	-2.703307	-3.305646
**127**	C	-6.057247	3.241618	2.599518
**128**	C	-7.108465	2.618740	3.530885
**129**	H	-7.760279	3.398168	3.940670
**130**	H	-7.740397	1.897313	3.004695
**131**	H	-6.628722	2.098975	4.366256
**132**	C	-5.260896	4.276579	3.396269
**133**	H	-4.748348	3.824625	4.251765
**134**	H	-4.515155	4.781179	2.773180
**135**	H	-5.941239	5.041431	3.783446
**136**	C	-6.762893	3.965728	1.441561
**137**	H	-7.387984	3.283194	0.858305
**138**	H	-7.406651	4.763326	1.828704
**139**	H	-6.031138	4.413978	0.761556

Table S34. Cartesian coordinates of the optimized T$_1'$ (^{3}IL) geometry of **3**

**1**	Au	-1.155197	0.414697	-0.945602	**48**	H	5.369038	0.565689	1.148147
**2**	O	1.092132	0.810135	2.751617	**49**	C	3.825294	3.207649	2.609920
**3**	O	2.221704	-3.044183	0.161974	**50**	H	4.000358	4.242204	2.887031
**4**	N	3.054601	-0.802904	1.591288	**51**	C	2.352154	1.307295	2.502442
**5**	N	-3.068806	-0.023101	-0.237057	**52**	C	-3.014850	-3.236587	1.635811
**6**	N	-0.815546	-1.423723	-2.049347	**53**	H	-2.332826	-3.919241	2.136775
**7**	C	-3.351337	-1.183882	0.450746	**54**	C	4.827352	2.452641	2.011479
**8**	C	1.361811	-0.446826	-2.026799	**55**	H	5.795727	2.891556	1.793092
**9**	C	3.964747	-1.839016	1.354923	**56**	C	-4.391847	-3.543369	1.595680
**10**	C	5.269697	-1.863871	1.849824	**57**	C	-4.746789	-1.455816	0.427153
**11**	H	5.612213	-1.059610	2.488770	**58**	C	4.377711	-3.983003	0.287213
**12**	C	-4.269932	0.479722	-0.680564	**59**	H	3.987035	-4.806413	-0.301906
**13**	C	-5.346660	-0.358062	-0.287732	**60**	C	-1.956333	6.313073	2.447207
**14**	C	0.498632	-1.573131	-2.381958	**61**	H	-1.141754	6.168154	3.164647
**15**	C	-1.744051	2.951601	0.574684	**62**	H	-2.764930	6.836945	2.966494
**16**	H	-2.754118	2.567358	0.677924	**63**	H	-1.598201	6.966882	1.645070
**17**	C	0.002544	-3.765028	-3.239584	**64**	C	0.897015	3.922194	0.356142
**18**	H	0.328403	-4.691534	-3.702200	**65**	H	1.913930	4.295284	0.283360
**19**	C	3.365968	0.521371	1.929061	**66**	C	6.135296	-2.904059	1.521104
**20**	C	2.777972	-0.402468	-2.153980	**67**	H	7.151835	-2.884765	1.901304
**21**	H	3.320824	-1.207486	-2.635602	**68**	C	1.694421	-1.126073	1.486756
**22**	C	3.519729	-2.944689	0.608635	**69**	C	-5.832218	1.952909	-1.730768
**23**	C	-2.475747	4.981276	1.902780	**70**	H	-6.023668	2.860824	-2.296775
**24**	C	-5.252945	-2.626325	1.000795	**71**	C	-0.821288	2.209230	-0.110925
**25**	H	-6.321445	-2.813512	0.964437	**72**	C	-0.624361	-0.612390	1.934506
**26**	C	0.924608	-2.757690	-2.982974	**73**	H	-1.357193	0.065089	2.358086
**27**	H	1.970540	-2.887241	-3.234371	**74**	C	-2.979870	4.135138	3.084333
**28**	C	-1.334658	-3.582010	-2.900159	**75**	H	-3.403827	3.184129	2.748948
**29**	H	-2.080374	-4.346242	-3.084631	**76**	H	-3.760793	4.676162	3.630208
**30**	C	0.727771	0.626525	-1.438356	**77**	H	-2.164224	3.914428	3.780501
**31**	C	-0.049861	-2.618003	0.716598	**78**	C	2.849703	1.800171	-1.062942
**32**	H	-0.328277	-3.510033	0.165701	**79**	H	3.421666	2.599068	-0.602860
**33**	C	3.513133	0.724221	-1.629845	**80**	C	7.145749	1.753458	-1.395776
**34**	C	-0.060786	4.653162	1.021023	**81**	H	7.712577	2.678279	-1.404899
**35**	H	0.225487	5.604704	1.456274	**82**	C	-4.876936	-4.851713	2.220893
**36**	C	0.564125	2.663743	-0.244920	**83**	C	1.417927	1.787135	-0.926634
**37**	C	-1.401487	4.202229	1.158146	**84**	C	-4.518786	1.643505	-1.418181
**38**	C	-1.026221	-1.774286	1.259398	**85**	H	-3.700661	2.287915	-1.729880
**39**	C	-1.702695	-2.383895	-2.298252	**86**	C	-6.924969	1.140656	-1.346374
**40**	H	-2.721995	-2.182153	-1.984196	**87**	C	5.771971	1.817472	-1.596016
**41**	C	0.724485	-0.313978	2.062706	**88**	H	5.304041	2.783590	-1.761436
**42**	C	4.980853	0.661111	-1.586182	**89**	C	5.638731	-0.568195	-1.419874
**43**	C	2.577963	2.630623	2.840353	**90**	H	5.066460	-1.489691	-1.376841
**44**	H	1.756983	3.193986	3.269998	**91**	C	7.009058	-0.624279	-1.218871
**45**	C	-6.661830	-0.020171	-0.625612	**92**	H	7.463799	-1.596742	-1.054271
**46**	H	-7.471959	-0.672160	-0.314203	**93**	C	5.702349	-3.955278	0.721994
**47**	C	4.597460	1.124550	1.662280	**94**	H	6.375374	-4.763596	0.455695
**95**	C	-8.340900	1.570088	-1.732432	**118**	C	9.987283	1.785689	-0.911320

**96**	C	-4.188281	-6.035646	1.524736	**119**	H	9.898441	2.278408	-1.885090
**97**	H	-4.431488	-6.051376	0.457147	**120**	H	11.053510	1.656137	-0.700685
**98**	H	-4.517058	-6.983990	1.965066	**121**	H	9.577481	2.454762	-0.147525
**99**	H	-3.099531	-5.982258	1.620084	**122**	C	-9.398935	0.579032	-1.244041
**100**	C	7.797545	0.533264	-1.184197	**123**	H	-9.249966	-0.416374	-1.675516
**101**	C	9.953906	-0.465974	-1.965742	**124**	H	-10.393932	0.925555	-1.542024
**102**	H	9.524576	-1.472081	-1.976466	**125**	H	-9.391926	0.485342	-0.153087
**103**	H	11.026970	-0.561805	-1.766724	**126**	C	9.487732	-0.210043	0.485996
**104**	H	9.828127	-0.035818	-2.964400	**127**	H	9.027049	0.409258	1.262724
**105**	C	-3.647455	5.268618	0.948058	**128**	H	10.554844	-0.308234	0.713996
**106**	H	-3.316735	5.877072	0.100017	**129**	H	9.038261	-1.206205	0.541355
**107**	H	-4.436537	5.816328	1.475188	**130**	C	-8.647022	2.941238	-1.110361
**108**	H	-4.087303	4.348405	0.551799	**131**	H	-8.585218	2.892672	-0.018378
**109**	C	-4.528210	-4.869075	3.717042	**132**	H	-9.656618	3.269865	-1.383112
**110**	H	-3.449939	-4.785566	3.882363	**133**	H	-7.942504	3.705798	-1.451217
**111**	H	-4.866766	-5.803791	4.178737	**134**	C	-2.470053	-2.072798	1.103737
**112**	H	-5.011630	-4.035269	4.236080	**135**	C	-8.449910	1.670037	-3.261661
**113**	C	9.293321	0.422832	-0.901421	**136**	H	-7.741523	2.396172	-3.671370
**114**	C	-6.389651	-5.030724	2.081390	**137**	H	-9.458469	1.984133	-3.554498
**115**	H	-6.939646	-4.231917	2.589588	**138**	H	-8.243478	0.701384	-3.728284
**116**	H	-6.691429	-5.981347	2.533041	**139**	C	1.289198	-2.266886	0.802445
**117**	H	-6.699530	-5.046976	1.031201					

NMR Spectra

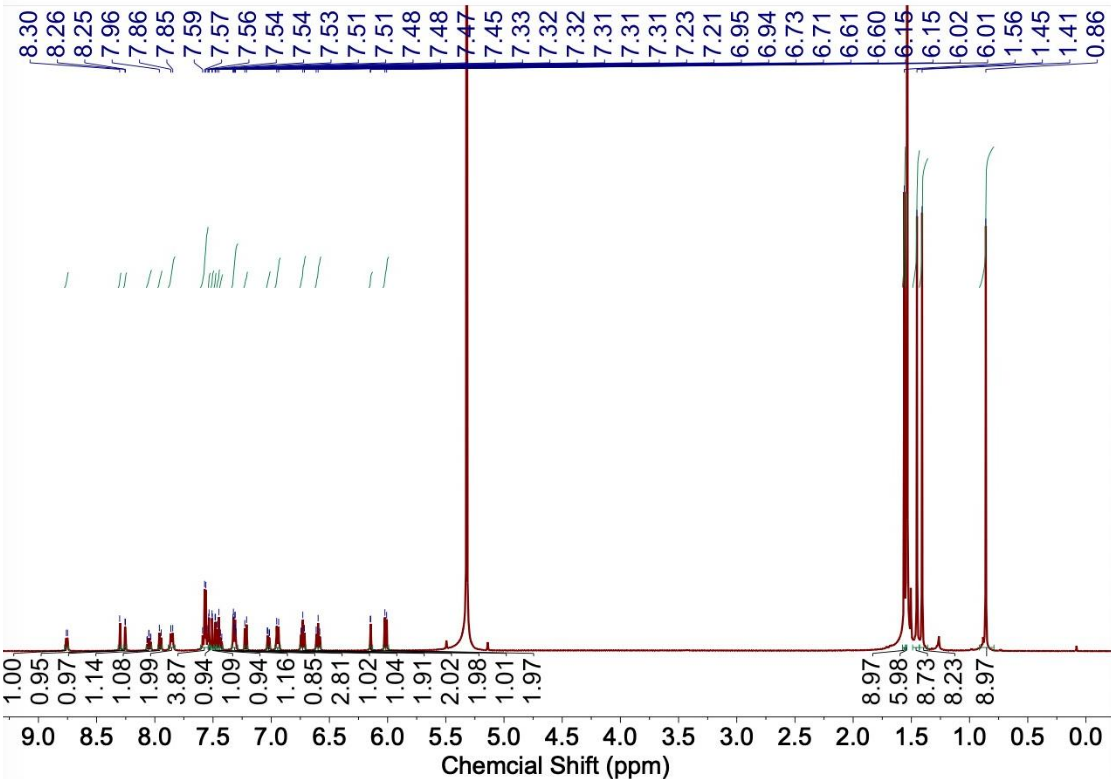

Figure S75. ^{1}H NMR spectrum of complex **1** in CD_2Cl_2.

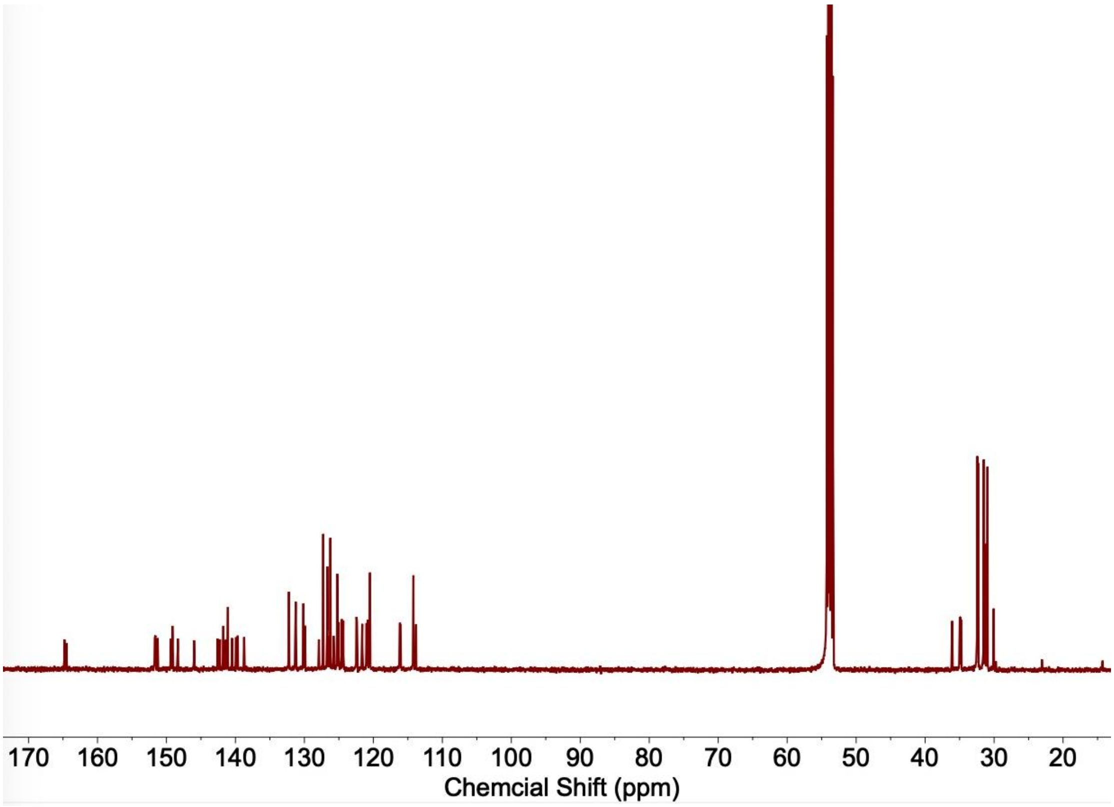

Figure S76. $^{13}C\{^{1}H\}$ NMR spectrum of complex **1** in CD_2Cl_2.

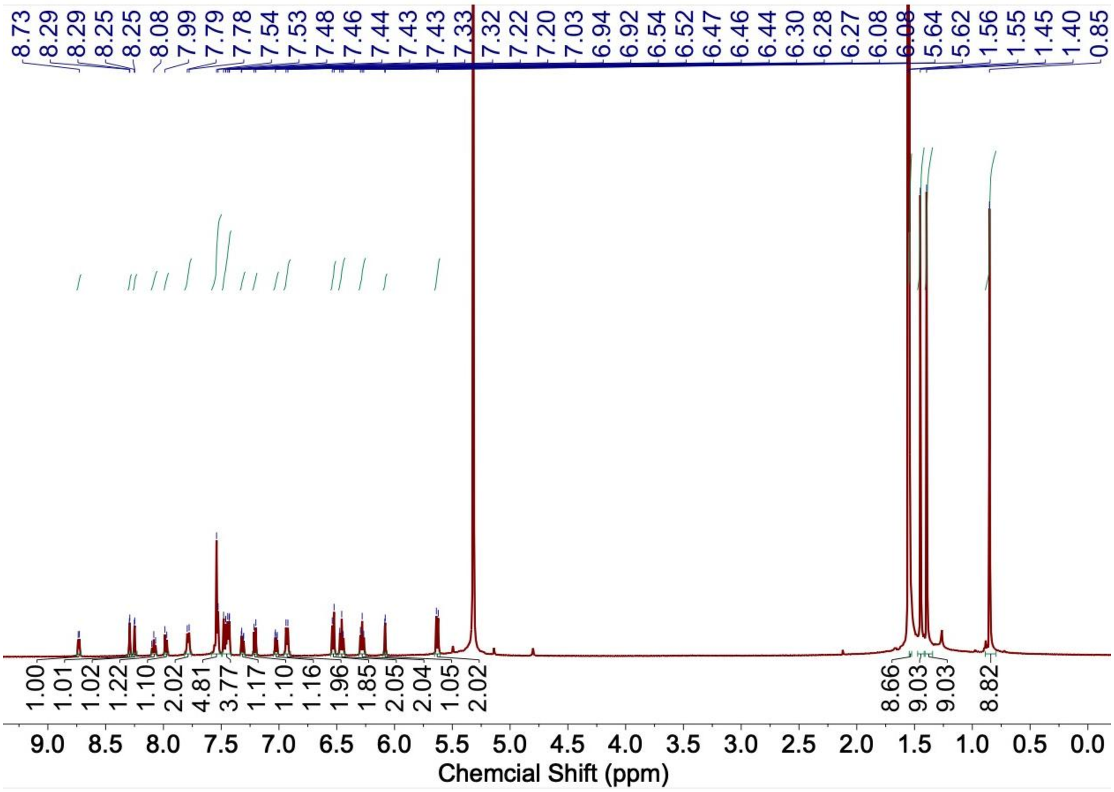

Figure S77. ^{1}H NMR spectrum of complex **2** in CD_2Cl_2.

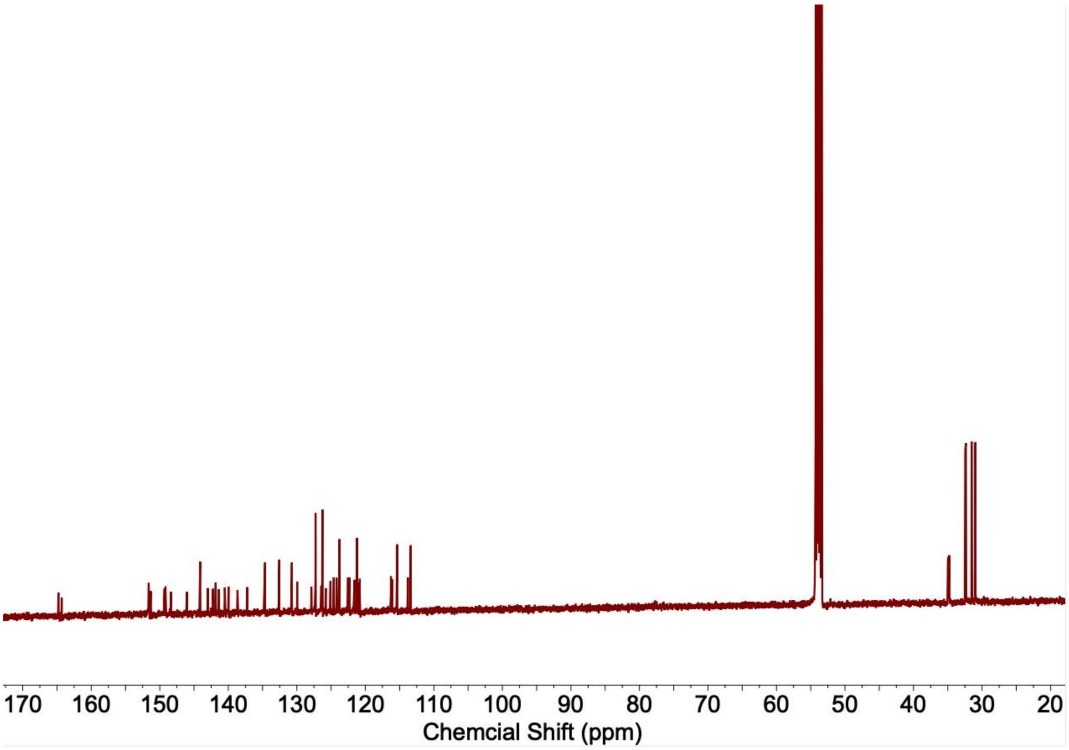

Figure S78. $^{13}C\{^{1}H\}$ NMR spectrum of complex **2** in CD_2Cl_2.

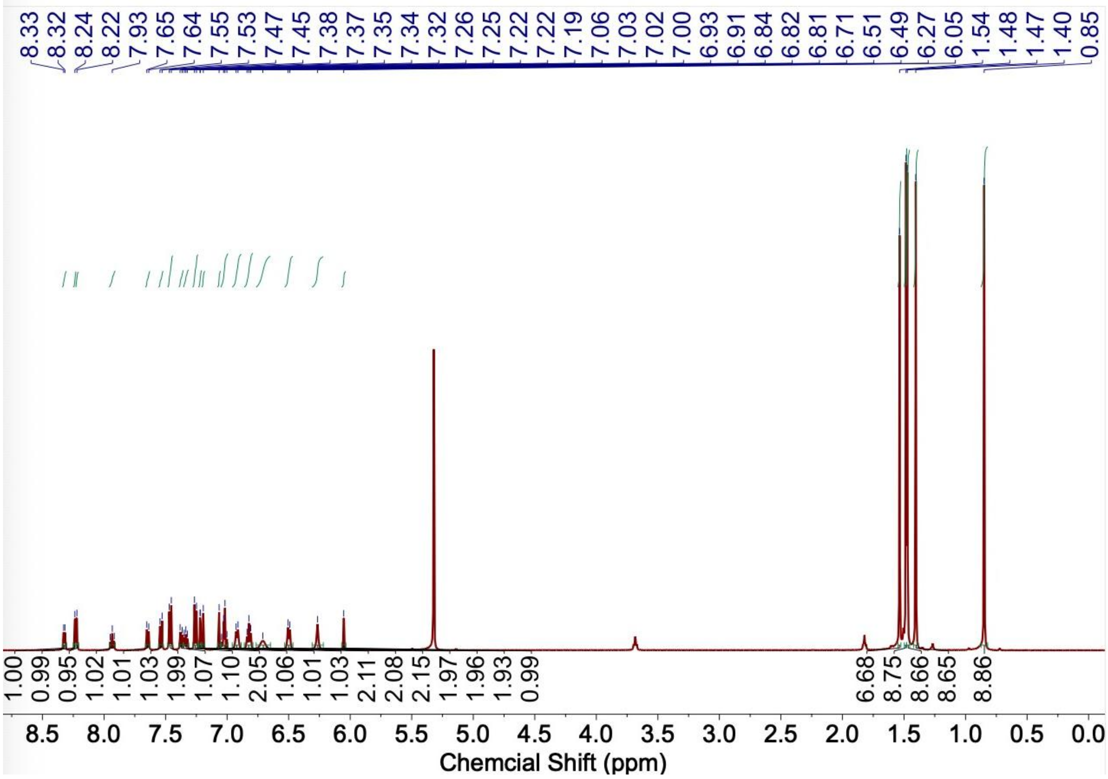

Figure S79. ^{1}H NMR spectrum of complex **3** in CD_2Cl_2.

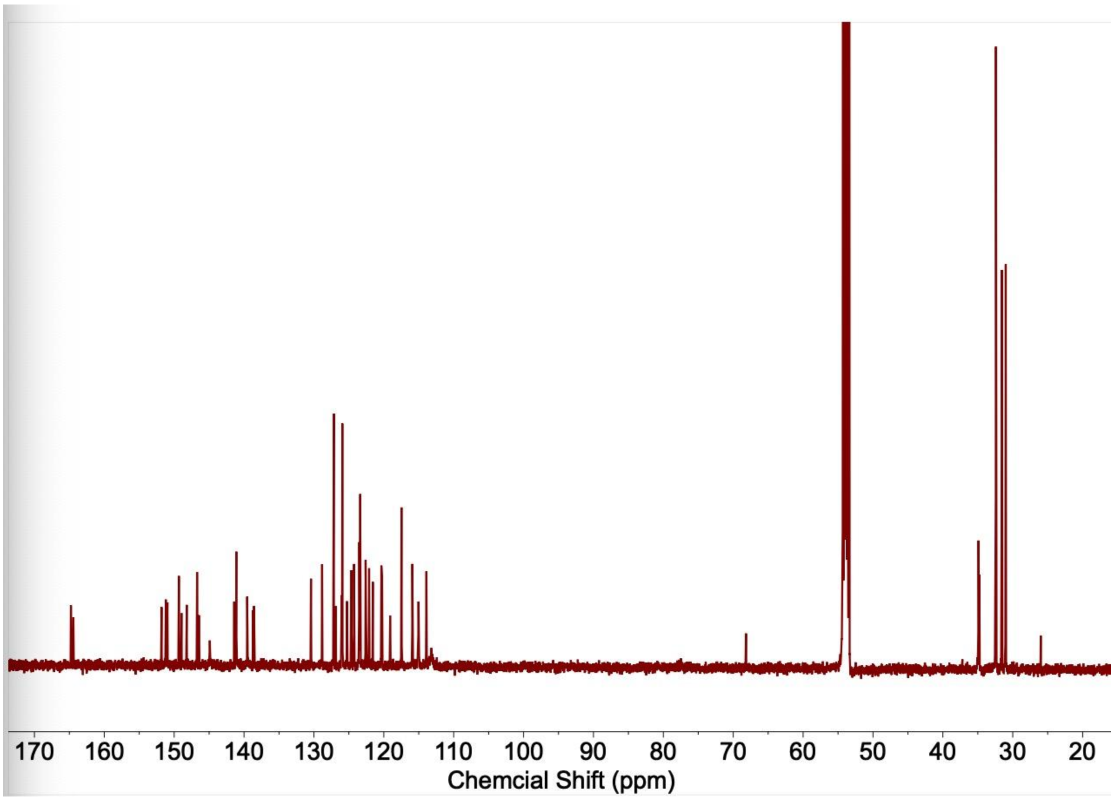

Figure S80. $^{13}C\{^{1}H\}$ NMR spectrum of complex **3** in CD_2Cl_2.

References

1. Crosby, G. A.; Demas, J. N., Measurement of Photoluminescence Quantum Yields. Review. *J. Phys. Chem.* **1971**, *75* (8), 991–1024.
2. Melhuish, W. H., Quantum Efficiencies of Fluorescence of Organic Substances: Effect of Sovent and Concentration of the Fluorescent Solute. *J. Phys. Chem.* **1961**, *65* (2), 229–235.
3. Van Der Sluis, P.; Spek, A. L., BYPASS: An Effective Method for the Refinement of Crystal Structures Containing Disordered Solvent Regions. *Acta Cryst. A* **1990**, *46* (3), 194–201.
4. Kiuchi, H.; Sonoda, Y.; Miyake, Y.; Kobayashi, F.; Tsutsumi, J. y.; Tadokoro, M.; Kanai, K., Mechanism of High Photoluminescence Quantum Yield of Melem. *Phys. Chem. Chem. Phys.* **2022**, *24* (38), 23602–23611.
5. Czerwieniec, R.; Yu, J.; Yersin, H., Blue-Light Emission of Cu(I) Complexes and Singlet Harvesting. *Inorg. Chem.* **2011,** *50* (17), 8293-301.
6. Tang, M.-C.; Leung, M.-Y.; Lai, S.-L.; Ng, M.; Chan, M.-Y.; Yam, V. W.-W., Realization of Thermally Stimulated Delayed Phosphorescence in Arylgold(III) Complexes and Efficient Gold(III) Based Blue-Emitting Organic Light-Emitting Devices. *J. Am. Chem. Soc.* **2018**, *140* (40), 13115-13124.
7. Gaussian 16, Revision A.03, Frisch, M. J.; Trucks, G. W.; Schlegel, H. B.; Scuseria, G. E.; Robb, M. A.; Cheeseman, J. R.; Scalmani, G.; Barone, V.; Petersson, G. A.; Nakatsuji, H.; Li, X.; Caricato, M.; Marenich, A. V.; Bloino, J.; Janesko, B. G.; Gomperts, R.; Mennucci, B.; Hratchian, H. P.; Ortiz, J. V.; Izmaylov, A. F.; Sonnenberg, J. L.; Williams-Young, D.; Ding, F.; Lipparini, F.; Egidi, F.; Goings, J.; Peng, B.; Petrone, A.; Henderson, T.; Ranasinghe, D.; Zakrzewski, V. G.; Gao, J.; Rega, N.; Zheng, G.; Liang, W.; Hada, M.; Ehara, M.; Toyota, K.; Fukuda, R.; Hasegawa, J.; Ishida, M.; Nakajima, T.; Honda, Y.; Kitao, O.; Nakai, H.; Vreven, T.; Throssell, K.; Montgomery, J. A., Jr.; Peralta, J. E.; Ogliaro, F.; Bearpark, M. J.; Heyd, J. J.; Brothers, E. N.; Kudin, K. N.; Staroverov, V. N.; Keith, T. A.; Kobayashi, R.; Normand, J.; Raghavachari, K.; Rendell, A. P.;

Burant, J. C.; Iyengar, S. S.; Tomasi, J.; Cossi, M.; Millam, J. M.; Klene, M.; Adamo, C.; Cammi, R.; Ochterski, J. W.; Martin, R. L.; Morokuma, K.; Farkas, O.; Foresman, J. B.; Fox, D. J. Gaussian, Inc., Wallingford CT, 2016.

8. Perdew, J. P.; Burke, K.; Ernzerhof, M., Generalized Gradient Approximation Made Simple. *Phys. Rev. Lett.* **1996**, *77* (18), 3865–3868.

9. Barone, V.; Cossi, M., Quantum Calculation of Molecular Energies and Energy Gradients in Solution by a Conductor Solvent Model. *J. Phys. Chem. A* **1998**, *102* (11), 1995–2001.

10. Contreras-García, J.; Johnson, E. R.; Keinan, S.; Chaudret, R.; Piquemal, J.-P.; Beratan, D. N; Yang, W., NCIPLOT: A Program for Plotting Noncovalent Interaction Regions. *J. Chem. Theory Comput.* **2011**, *7* (3), 625–632.

11. Lu, T.; Chen, F., Multiwfn: A Multifunctional Wavefunction Analyzer. *J. Comput. Chem.* **2012**, *33* (5), 580–592.

12. Kwok, W.-K.; Li, L.-K.; Lai, S.-L.; Leung, M.-Y.; Tang, W. K.; Cheng, S.-C.; Tang, M.-C.; Cheung, W.-L.; Ko, C.-C.; Chan, M.-Y.; Yam, V. W.-W., Tetradentate C^C^N^N Ligand-Containing Gold(III) Complexes with Orange to Deep-Red Thermally Activated Delayed Fluorescence (TADF) and Their Application in Organic Light-Emitting Devices. *J. Am. Chem. Soc.* **2023**, *145* (17), 9584–9595.

13. Leung, M.-Y.; Tang, M.-C.; Cheng, S.-C.; Chen, Z.; Lai, S.-L.; Tang, W. K.; Chan, M.-Y.; Ko, C.-C.; Yam, V. W.-W., Molecular Design and Synthetic Approaches for the Realization of Multichannel Radiative Decay Pathways in Gold(III) Complexes and Their Applications in Organic Light-Emitting Devices. *J. Am. Chem. Soc.* **2024**, *146*, 30901–30912.

14. Romanov, A. S.; Jones, S. T. E.; Gu, Q.; Conaghan, P. J.; Drummond, B. H.; Feng, J.; Chotard, F.; Buizza, L.; Foley, M.; Linnolahti, M.; Credgington, D.; Bochmann, M., Carbene–Metal–Amide Photoemitters: Tailoring Conformationally Flexible Amides for Full Color Range Emissions Including White-Emitting OLED. *Chem. Sci.* **2020**, *11*, 435.

15. Yang, J.-G.; Song, X.-F.; Cheng, G.; Wu, S.; Feng, X.; Cui, G.; To, W.-P.; Chang,

X.; Chen, Y.; Che, C.-M.; Yang, C.; Li, K., Conformational Engineering of Two-Coordinate Gold(I) Complexes: Regulation of Excited-State Dynamics for Efficient Delayed Fluorescence *ACS Appl. Mater. Interfaces* **2022**, *14*, 13539.

16. Zhan, L.; Ying, A.; Qi, Y.; Wu, K.; Tang, Y.; Tan, Y.; Zou, Y.; Xie, G.; Gong, S.; Yang, C., *Adv. Funct. Mater.* Copper(I) Complex as Sensitizer Enables High-Performance Organic Light-Emitting Diodes with Very Low Efficiency Roll-Off **2021**, *31*, 2106345.

17. Jiang, R.; Wu, X.; Liu, H.; Guo, J.; Zou, D.; Zhao, Z.; Tang, B. Z., High-Performance Orange-Red Organic Light-Emitting Diodes with External Quantum Efficiencies Reaching 33.5% based on Carbonyl-Containing Delayed Fluorescence Molecules. *Adv. Sci.* **2022**, *9*, e2104435.
